# Catalytic arylsulfonyl radical-triggered 1,5-enyne-bicyclizations and hydrosulfonylation of α,β-conjugates†
†Electronic supplementary information (ESI) available. CCDC 1406678 (**3f**). For ESI and crystallographic data in CIF or other electronic formats see DOI: 10.1039/c5sc02343b



**DOI:** 10.1039/c5sc02343b

**Published:** 2015-11-13

**Authors:** Zhen-Zhen Chen, Shuai Liu, Wen-Juan Hao, Ge Xu, Shuo Wu, Jiao-Na Miao, Bo Jiang, Shu-Liang Wang, Shu-Jiang Tu, Guigen Li

**Affiliations:** a Jiangsu Key Laboratory of Green Synthetic Chemistry for Functional Materials , Jiangsu Normal University , Xuzhou , 211116 , P. R. China . Email: jiangchem@jsnu.edu.cn ; Email: laotu@jsnu.edu.cn ; Fax: +8651683500065 ; Tel: +8651683500065; b Institute of Chemistry & BioMedical Sciences , Collaborative Innovation Center of Chemistry for Life Sciences , School of Chem. and Chem. Eng. , Nanjing University , Nanjing 210093 , P. R. China; c Department of Chemistry and Biochemistry , Texas Tech University , Lubbock , TX 79409-1061 , USA . Email: guigen.li@ttu.edu

## Abstract

A catalytic bicyclization reaction of 1,5-enynes anchored by α,β-conjugates with arylsulfonyl radicals generated *in situ* from sulfonyl hydrazides has been established using TBAI (20 mol%) and Cu(OAc)_2_ (5 mol%) as co-catalysts under convenient conditions. In addition, the use of benzoyl peroxide (BPO) as the oxidant and pivalic acid (PivOH) as an additive was proven to be necessary for this reaction. The reactions occurred through 5-*exo*-dig/6-*endo*-trig bicyclizations and homolytic aromatic substitution (HAS) cascade mechanisms to give benzo[*b*]fluorens regioselectively. A similar catalytic process was developed for the synthesis of γ-ketosulfones. These reactions feature readily accessible starting materials and simple one-pot operation.

## Abstract 2

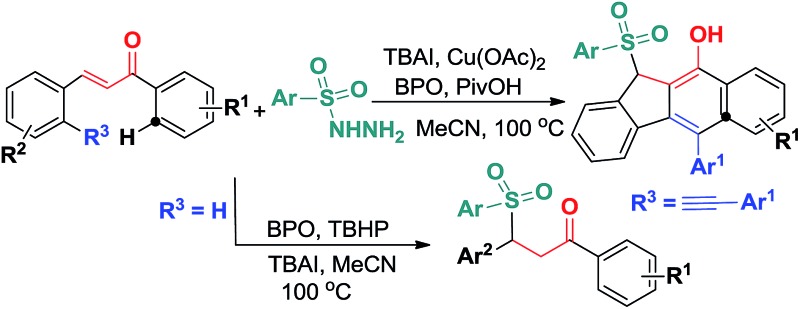
Catalytic bicyclization of 1,5-enynes anchored by α,β-conjugates with arylsulfonyl radicals was established using TBAI and Cu(OAc)_2_ as co-catalysts.

The search for efficient cyclization reactions, particularly for those in radical cascade processes, has been actively pursued in the past several decades because they are extremely useful for the total synthesis of numerous important targets.^1,2^ These reactions enable the rapid, reliable and straightforward creation of multicyclic ring systems using readily available starting materials with features such as unparalleled efficiencies, high functional tolerance and convenient conditions.^2^ Among these cyclization reactions, the majority of efforts have been devoted to conducting radical ene-cyclization cascades, in which terminal alkenes were utilized in most cases *via* either metal-free or transition-metal-mediated radical processes (Scheme 1, eqn (1)).^3^ However, the use of internal alkenes as radical acceptors has been highly challenging (Scheme 1, eqn (2))^4^ owing to their relatively low reactivity and larger steric hindrance as compared with their terminal counterparts.

**Scheme 1:**
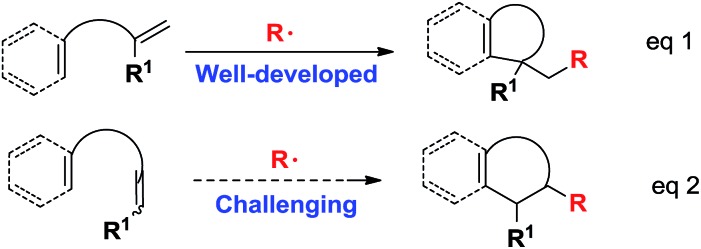
Two modes of radical ene-cyclizations.

1,5-Enynes endowed with extra unsaturated moieties are remarkable building blocks, and have been widely used for direct and selective tandem cyclizations *via* synergistic additions across C=C and C≡C bonds in a one-step operation.^5^ These cyclizations enhance both bond formation and annulation efficiencies with high levels of structural complexity and a reduced generation of waste. So far, two main methods for 1,5-enyne cyclizations have been developed through metal catalysis^6^ or electrophilic cyclization.^7^ However, the radical bicyclization of 1,5-enynes for generating multi-substituted polycycles has not been well documented. The literature survey revealed that sulfonyl radicals can be generated from sulfonyl hydrazides and utilized *in situ* for the radical sulfonylation of alkenes.^8^ Due to the importance of sulfonyl-containing compounds in photovoltaic materials, nonlinear optics and in general synthetic and medicinal areas,^9^ we envisioned that under suitable catalytic radical conditions, the *in situ* generated sulfonyl radicals would be able to be involved in cascade bond-forming events with the internal C=C and C≡C bonds of 1,5-enyne conjugate systems, resulting in 5-*exo*-dig/6-*endo*-trig bicyclizations and homolytic aromatic substitutions (HASs) (Scheme 2). Herein, we would like to report the preliminary results of this endeavour (Scheme 2).

**Scheme 2:**
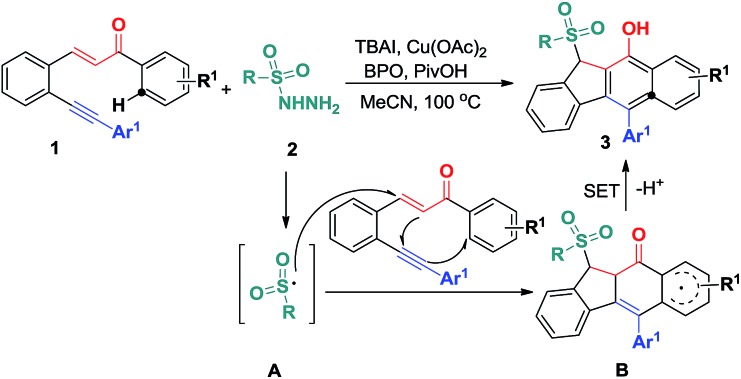
Envisaged new reactivity of 1,5-enynes.

At first, 3-(2-(phenylethynyl)phenyl)-1-(*p*-tolyl)prop-2-en-1-one **1a** was selected as a benchmark substrate to investigate the additions by sulfonyl radicals. With 20 mol% tetrabutylammoniumiodide (TBAI) as the catalyst, the reaction of substrate **1a** with tosylhydrazide **2a** was performed in CH_3_CN in the presence of benzoperoxide (BPO) (4.0 equiv.) as an oxidant at 70 °C under air conditions, affording the expected benzo[*b*]fluorens **3a**, albeit with a low yield of 18% (Table 1, entry 1). Other solvents, such as dichloromethane (DCM), 1,4-dioxane and toluene, were also examined, with CH_3_CN showing the best performance (entries 2–4). Raising the reaction temperature to 100 °C slightly ameliorates the yield of **3a** (entry 5). A subsequent investigation of other catalysts was conducted in CH_3_CN. As illustrated in entries 6–8, different types of catalysts including I_2_, KI, and CuI were employed in the model reaction, and it turned out that I_2_ and KI hardly facilitate the reaction (entries 6 and 7), while CuI as a catalyst only led to a poor yield of 16%. Next, we turned our attention to evaluating different additives (entries 9–11). We found that the addition of PivOH (1.0 equiv.) delivered **3a** in a 35% yield (entry 11). Notably, the reaction of **1a** and **2a** in the presence of 2.0 equiv. of PivOH gave **3a** in a 71% yield using a co-catalyst of TBAI (20 mol%) and Cu(OAc)_2_ (5 mol%) with complete consumption of the starting material **1a** (entry 15). Without PivOH, the yield of the expected product **3a** decreased remarkably (entry 17). Further screening of other oxidants, such as TBHP (64% yield), DTBP (very poor yield) and H_2_O_2_ (no product) for this transformation showed that BPO was the best choice (See ESI†).

**Table 1:** Optimization of the reaction conditions^*a*^

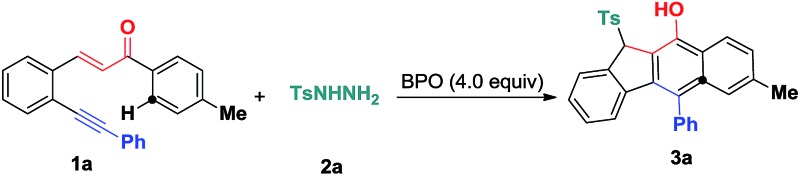					
Entry	Catalyst (mol%)	Additives (equiv.)	Solvent	*T* (°C)	Yield^*b*^ (%)
1	TBAI (20)	—	MeCN	70	18
2	TBAI (20)	—	DCM	70	10
3	TBAI (20)	—	1,4-Dioxane	70	Trace
4	TBAI (20)	—	Toluene	70	0
5	TBAI (20)	—	MeCN	100	25
6	I_2_ (15)	—	MeCN	100	Messy
7	KI (20)	—	MeCN	100	Messy
8	CuI (20)	—	MeCN	100	16
9	TBAI (20)	HOAc (1.0)	MeCN	100	28
10	TBAI (20)	l-Proline (1.0)	MeCN	100	33
11	TBAI (20)	PivOH (1.0)	MeCN	100	35
12	TBAI (20)/CuI (5)	PivOH (1.0)	MeCN	100	49
13	TBAI (30)/Cu(OAc)_2_ (5)	PivOH (1.0)	MeCN	100	53
14	TBAI (20)/Cu(OAc)_2_ (5)	PivOH (1.0)	MeCN	100	61
15	TBAI (20)/Cu(OAc)_2_ (5)	PivOH (2.0)	MeCN	100	71
16	TBAI (20)/Cu(OAc)_2_ (10)	PivOH (2.0)	MeCN	100	63
17	TBAI (20)/Cu(OAc)_2_ (5)	—	MeCN	100	33

^*a*^Reaction conditions: 1,5-conjugated enyne (**1a**, 0.25 mmol), tosylhydrazide (**2a**, 0.50 mmol), BPO (1.0 mmol), solvent (2.5 mL), 12 h.

^*b*^Isolated yields based on **1**.

With the optimized reaction conditions in hand, we examined the substrate scope of the sulfonyl hydrazides **2** by treating them with 1,5-enynes **1a** (Scheme 3). As anticipated, the substituents on the phenyl ring of the arylsulfonyl hydrazides **2** did not hamper the catalytic process, but affected the reaction efficiency. Reactions of methyl- or bromo, *t*-butyl-substituted arylsulfonyl hydrazide **2** with **1a** afforded the desired products in moderate to good yields. Additionally, benzenesulfonohydrazide exhibited a higher reactivity, allowing 1,5-enyne-bicyclization cascades toward the formation of benzo[*b*]fluorens **3c** in an 84% yield. 1,5-Enynes bearing an electron-donating or electron-withdrawing group (methoxy and chloro) at the *para* position of the aromatic ring (Ar^1^) directly bound to the C≡C bond gave the corresponding sulfonated products **3e** and **f** in 55% and 68% yields, respectively. Alternatively, a naphthalen-1-yl substituent linked to the C≡C bond was also well-tolerated, affording the product **3g** in a 60% chemical yield. Similarly, either electron-donating (methyl) or electron-withdrawing (bromo) groups (R^1^) at the *para* position of the phenyl ring tethered to the enone unit were well-suited for these radical 1,5-enyne-bicyclizations (**3a–3k**). 1,5-Enynes **1** carrying electron-neutral groups were also smoothly converted into the corresponding sulfonated benzo[*b*]fluorens **3l–3n** in 39–67% yields. Notably, 2-naphthalenylethanone-derived 1,5-enynes furnished the unprecedented pentacyclic indeno[2,1-*b*]phenanthren-7-ol **3n** in a 67% chemical yield though sulfonyl radicals triggered the 1,5-enyne-bicyclization. Unfortunately, a bulky *ortho*-Br substituent and benzylsulfonyl hydrazide did not work at all (**3o** and **3p**). Besides the NMR and HR-MS spectroscopic analysis for benzo[*b*]fluorens **3**, the X-ray diffraction for product **3f** has been performed as shown in Fig. 1.

**Scheme 3:**
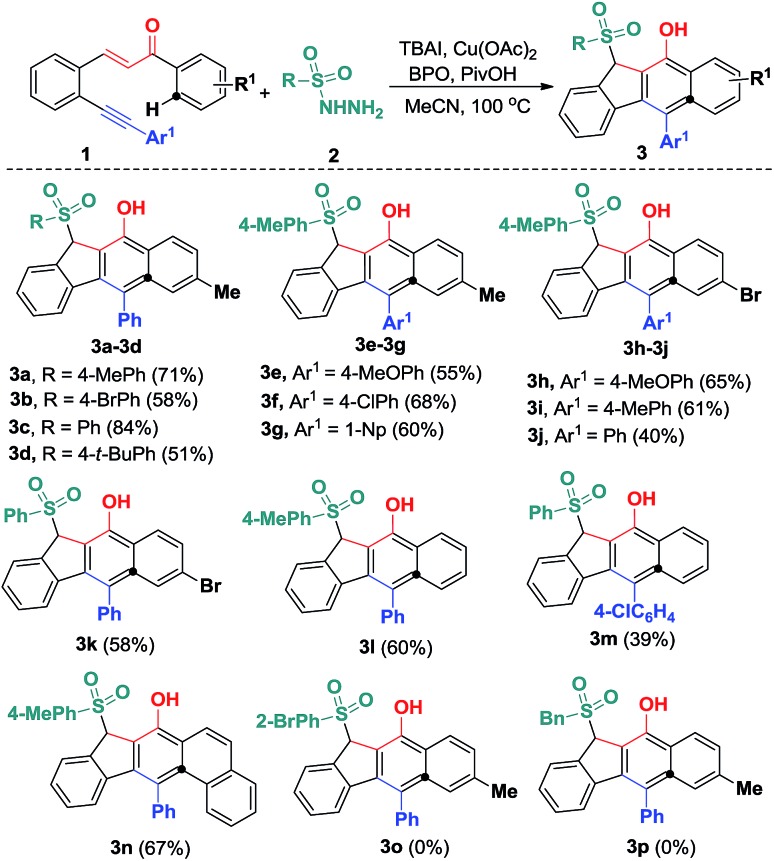
Substrate scope of the hydrosulfonylation reaction. Reaction conditions: 1,5-conjugated enyne (**1**, 0.25 mmol), sulfonyl hydrazide (**2**, 0.50 mmol), TBAI (0.05 mmol), Cu(OAc)_2_ (0.0125 mmol), PivOH (0.50 mmol), BPO (1.0 mmol), CH_3_CN (2.5 mL), 100 °C, 12 h. Isolated yields based on **1**.

**Fig. 1:**
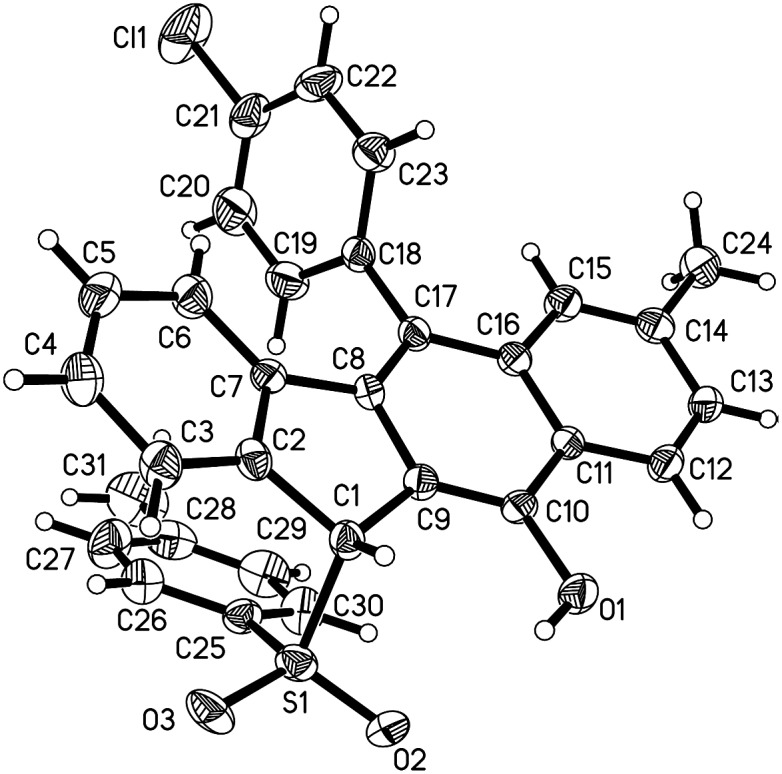
The ORTEP drawing of **3f**.

In view of our success with the synthesis of functional benzo[*b*]fluorens **3**, we reasoned that in the absence of alkyne moieties, chalcones **4** would be able to accept sulfonyl radicals *via* typical 1,4-additions, which would expand their utility for the synthesis of γ-ketosulfones. We thus explored this possibility through a one-pot reaction of (4-chlorophenyl)-3-phenylprop-2-en-1-one (**4a**) with **2a** under the conditions described above. The expected γ-ketosulfone **5a** was obtained but with a lower yield (15%) initially. After careful optimizations were performed, we found that although Cu(OAc)_2_ and PivOH did promote this catalytic process, the use of co-oxidants of BPO (2.0 equiv.) and TBHP (1.0 equiv., 70% in water) in 20 mol% of TBAI proved to be suitable for the current hydrosulfonylation, furnishing product **5a** in a 77% yield. Subsequently, we further studied the reaction scope by reacting arylsulfonyl hydrazides **2** with various chalcones **4** under these conditions (Scheme 4). It turned out that the presence of various substituents, including methoxyl, methyl, chloro and bromo groups, on the aryl rings of the chalcones all worked well, giving access to a wide range of γ-ketosulfones **5a–5o** with yields ranging from 50% to 90%. Alternatively, arylsulfonyl hydrazides **2** carrying either electronically neutral or rich groups can be successfully engaged in this catalysis. Unfortunately, aliphatic sulfonyl hydrazide (**5p**) was proven not to be an adaptable substrate for this reaction, which may be ascribed to the relative instability of the sulfonyl radicals generated *in situ* from aliphatic sulfonyl hydrazides. Joining previously reported work,^10^ this catalytic radical addition provided a new protocol for the formation of γ-ketosulfones, which are important building blocks in organic synthesis.

**Scheme 4:**
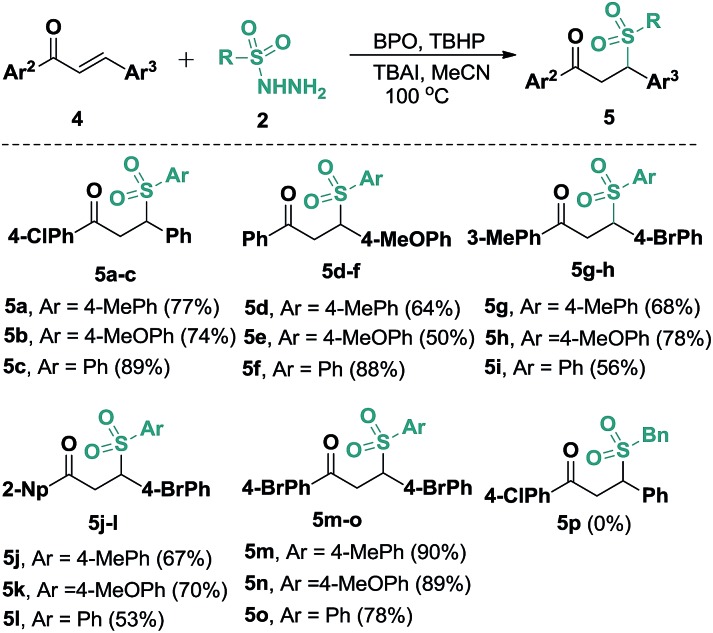
Substrate scope of the synthesis of γ-ketosulfones. Reaction conditions: chalcone (**4**, 0.25 mmol), sulfonyl hydrazide (**2**, 0.50 mmol), TBAI (0.05 mmol), BPO (0.50 mmol), TBHP (0.25 mmol, 70% in water), CH_3_CN (2.5 mL), 100 °C, 6 h. Isolated yields based on **4**.

To understand the mechanism, several control experiments were conducted. The treatment of 1,5-enyne **1h** with tosylhydrazide **2a** in the presence of radical scavenger TEMPO (4.0 equiv.) under standard conditions gave complex mixtures without the observation of the desired product **3l**, confirming the existence of a radical mechanism (Scheme 5, eqn (1)). In the absence of BPO, the reaction did not show the desired product (eqn (2)). To further confirm the sulfonylation sequence, subjecting 1,5-enyne **1h** to the standard conditions in the absence of **2a** failed to generate any desired benzo[*b*]fluoren product **6** (eqn (3)). These control experiments suggest that BPO is essential for the catalytic cycles and the *in situ* generated sulfonyl radical triggers a 5-*exo*-dig/6-*endo*-trig bicyclization cascade.

**Scheme 5:**
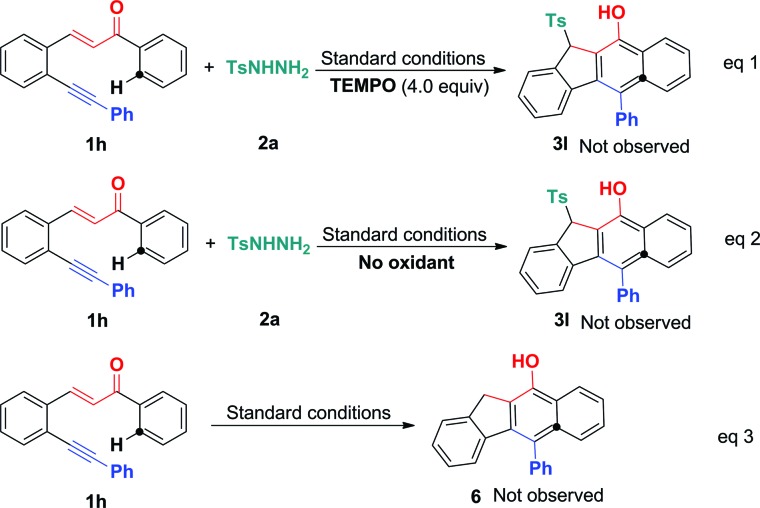
Control reactions. Reaction conditions: 1,5-conjugated enyne (**1h**, 0.25 mmol), tosylhydrazide (**2a**, 0.50 mmol), TBAI (0.05 mmol), Cu(OAc)_2_ (0.0125 mmol), PivOH (0.50 mmol), BPO (1.0 mmol), CH_3_CN (2.5 mL), 100 °C, 12 h.

On the basis of the above observations and those reported in literature,^8,11^ a mechanism is proposed and shown in Scheme 6. The first step is to form the sulfonyl radical **A** from the sulfonyl hydrazide using the benzoyloxy radical generated *in situ* from the I^–^ anion-assisted decomposition of BPO. The intermolecular addition of the resulting sulfonyl radical **A** and the 1,5-conjugated enyne **1** followed by a 5-*exo*-dig cyclization gives intermediate **B**, in which the homolysis of carbon–copper(iii) affords vinyl radical **C**. Intermediate **C** is converted into aryl radical **D** *via* a *6-endo-trig* cyclization. Intermediate **D** undergoes SET (single electron transfer) oxidation and subsequent deprotonation to provide intermediate **F**. The tautomerization of **F** leads to the formation of benzo[*b*]fluorens **3**. Although the generation of sulfonyl radicals triggered by various oxidants has been achieved well,^8^ the bicyclizations^12^ towards fused carbocycles *via* sulfonyl radical initiated bifunctionalization of enynes is very rare in organic chemistry as mentioned earlier.

**Scheme 6:**
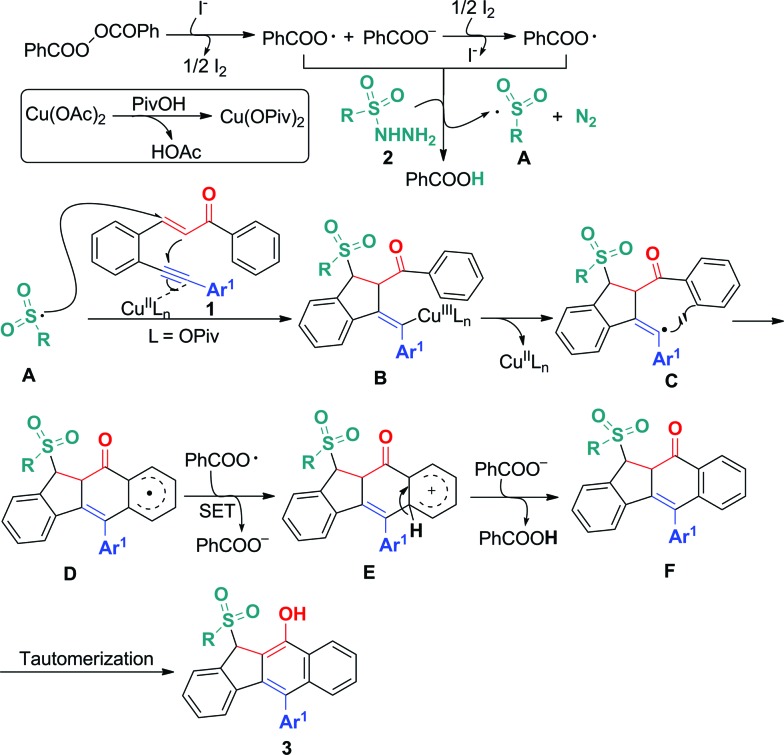
Proposed mechanism for forming products **3**.

## Conclusions

In summary, we have discovered new 1,5-enyne-bicyclization and hydrosulfonylation reactions of α,β-conjugates under convenient co-catalytic conditions. The addition of *in situ* generated sulfonyl radicals across the activated double bond is able to trigger a cascade 5-*exo*-dig/6-*endo*-trig bicyclization and HAS sequence, delivering tetracyclic sulfonylated benzo[*b*]fluorens in a successive C–S and C–C bond-forming process. Using chalcones as replacements for 1,5-conjugated enynes, this reaction enables the hydrosulfonylation of alkenes to form γ-ketosulfones with good to excellent yields. These two methods allow easy access to important functional sulfones for potential applications in organic and medicinal chemistry.
