# Supplementary material for: Catalytic arylsulfonyl radical-triggered 1,5-enyne-bicyclizations and hydrosulfonylation of α,β-conjugates
Source: Chem Sci. 2015 Aug 19;6(11):6654–8. doi: 10.1039/c5sc02343b (PMC4608173; doi:10.1039/c5sc02343b)
Supplement: Supplementary file 1 [file SC-006-C5SC02343B-s001.pdf]

Electronic Supplementary Material (ESI) for Chemical Science.
This journal is © The Royal Society of Chemistry 2015

# Supporting Information

## Catalytic arylsulfonyl radical-triggered 1,5-enyne-bicyclizations and hydrosulfonylation of α,β-conjugates

Zhen-Zhen Chen,[a,1] Shuai Liu,[a,1] Wen-Juan Hao,[a] Ge Xu,[a] Shuo Wu,[a] Jiao-Na Miao,[a] Bo Jiang,[a,]* Shu-Liang Wang,[a] Shu-Jiang Tu[a,]* and Guigen Li [b,c,]*

[a] *School of Chemistry and Chemical Engineering, Jiangsu Normal University, P. R. China; E-mail: jiangchem@jsnu.edu.cn; laotu@jsnu.edu.cn;*
[b]*Institute of Chemistry & BioMedical Sciences, Collaborative Innovation Center of Chemistry for Life Sciences, School of Chem. and Chem. Eng., Nanjing University, Nanjing 210093, P. R. China; and* [c] *Department of Chemistry and Biochemistry, Texas Tech University, Lubbock, TX 79409-1061, USA; United States; email:guigen.li@ttu.edu (G.L.);*[1] These authors are equal to this work

## Table of Contents

General information-------------------------------------------------------------------S2

X-ray Structure of Compounds **3f**---------------------------------------------------S2

**Table 1** Optimization of the reaction conditions for Compound **3a**----------------S3

General Procedure for the Synthesis of Compounds **1a** and **3a**---------------------S5

Characterization Data of Compounds **3a-n**--------------------------------------S5-S9

General Procedure for the Synthesis of Compounds **5a**----------------------------S9

Characterization Data of Compounds **5a-o**------------------------------------S9-S14

Copies of $^{1}$H and $^{13}$C NMR Spectra for Compounds **3a-n**-------------------S14-S27

Copies of $^{1}$H and $^{13}$C NMR Spectra for Compounds **5a-o**------------------S28-S42

## Experimental

## General Information

Unless otherwise noted all starting materials were either known compounds or were obtained from commercial sources and used without purification. Melting points were determined in open capillaries. IR spectra were taken on a FT-IR-Tensor 27 spectrometer. $^1$H NMR ($^{13}$C NMR) spectra were measured on a Bruker DPX 400 MHz spectrometer in $CDCl_3$ (or DMSO-$d_6$) with chemical shift (δ) given in ppm relative to TMS as internal standard [(*s* = singlet, *d* = doublet, *t* = triplet, brs = broad singlet, m = multiplet), coupling constant (Hz)]. HRMS (APCI-TOF) was determined by using microTOF-QⅡ HRMS/MS instrument (BRUKER). X-Ray crystallographic analysis was performed with a Siemens SMART CCD and a Siemens P4 diffractometer.

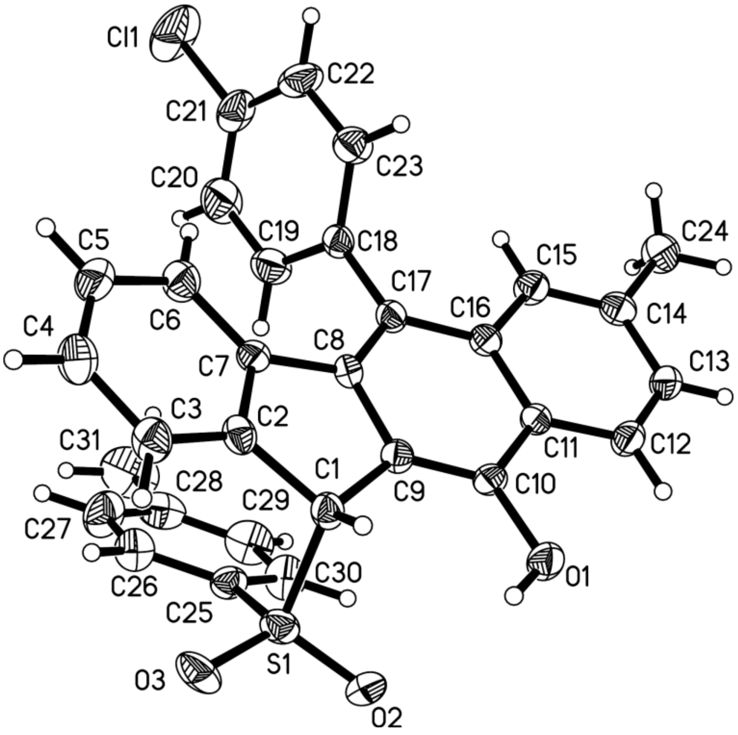


**Fig 1**, X-ray Structure of **3f**

**Table 1** Optimization of the reaction conditions[a]

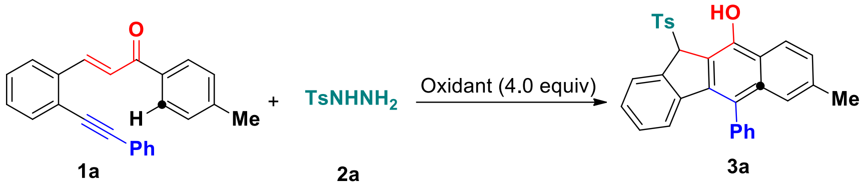


| Entry | Oxidant (equiv) | Catalyst (mol%) | Additives (equiv) | Solvent | T (°C) | Yield[b] (%) |
|---|---|---|---|---|---|---|
| 1 | BPO(4.0) | TBAI (20) | - | MeCN | 70 | 18 |
| 2 | BPO(4.0) | TBAI (20) | - | DCM | 70 | 10 |
| 3 | BPO(4.0) | TBAI (20) | - | 1,4-Dioxane | 70 | trace |
| 4 | BPO(4.0) | TBAI (20) | - | Toluene | 70 | 0 |
| 5 | BPO(4.0) | TBAI (20) | - | MeCN | 100 | 25 |
| 6 | BPO(4.0) | $I_2$ (15) | - | MeCN | 100 | messy |
| 7 | BPO(4.0) | KI (20) | - | MeCN | 100 | messy |
| 8 | BPO(4.0) | CuI (20) | - | MeCN | 100 | 16 |
| 9 | BPO(4.0) | TBAI (20) | HOAc (1.0) | MeCN | 100 | 28 |
| 10 | BPO(4.0) | TBAI (20) | L-proline (1.0) | MeCN | 100 | 33 |
| 11 | BPO(4.0) | TBAI (20) | PivOH (1.0) | MeCN | 100 | 35 |
| 12 | BPO(4.0) | TBAI (20)/ CuI (5) | PivOH (1.0) | MeCN | 100 | 49 |
| 13 | BPO(4.0) | TBAI (30)/ $Cu(OAc)_2$ (5) | PivOH (1.0) | MeCN | 100 | 53 |
| 14 | BPO(4.0) | TBAI (20)/ $Cu(OAc)_2$ (5) | PivOH (1.0) | MeCN | 100 | 61 |
| 15 | BPO(4.0) | TBAI (20)/ $Cu(OAc)_2$ (5) | PivOH (2.0) | MeCN | 100 | 71 |
| 16 | BPO(4.0) | TBAI (20)/ $Cu(OAc)_2$ (10) | PivOH (2.0) | MeCN | 100 | 63 |
| 17 | TBHP (4.0) | TBAI (20/ /$Cu(OAc)_2$ (5) | PivOH (2.0) | MeCN | 100 | 64 |
| 18 | DTBP(4.0) | TBAI (20/ /$Cu(OAc)_2$ (5) | PivOH (2.0) | MeCN | 100 | trace |
| 19 | $H_2O_2$(4.0) | TBAI (20/ /$Cu(OAc)_2$ (5) | PivOH (2.0) | MeCN | 100 | trace |
| 20 | BPO(4.0) | $Cu(OAc)_2$ (5) | PivOH (2.0) | MeCN | 100 | 20 |
| 21 | BPO(4.0) | TBAI (20)/ $Cu(OAc)_2$ (5) | - | MeCN | 100 | 33 |

[a]Reaction conditions: 1,5-conjugated enyne (**1a**, 0.25 mmol), tosylhydrazide (**2a**, 0.50 mmol), Oxidant (1.0 mmol), solvent (2.5 mL), 12 h. [b]Isolated yields based on **1**.

## Experimental Section

**Preparation of Compound 1a:**

A mixture of 2-(phenylethynyl)benzaldehyde (5 mmol), *p*-tolylethanone (0.67 g, 5 mmol), NaOH ( 0.8g, 20 mmol) and EtOH (20.0 mL) were added in a 50-mL reaction vial. Then, the mixture was stirred at 0 °C for 20 min, and the followed reaction system was stirred at room temperature. After completion of the reaction monitored by TLC, the reaction system was poured into the cold water, and the solid product was collected by Büchner filtration. The desired pure 3-(2-(phenylethynyl)phenyl)-1-(*p*-tolyl)prop-2-en-1-one (**1a**) was obtained in 80% yield by recrystallization from 95% EtOH.

**Preparation of Compound 3a: 7-Methyl-5-phenyl-11-tosyl-11*H*-benzo[*b*]fluoren-10-ol**

A mixture of 3-(2-(phenylethynyl)phenyl)-1-(*p*-tolyl)prop-2-en-1-one (**1a**, 0.25 mmol, 1.0 equiv.), tosylhydrazide (**2a**, 0.5 mmol, 2.0 equiv.), BPO (1.0 mmol, 4.0 equiv.), PivOH (0.5 mmol, 2.0 equiv.), TBAI (0.05 mmol, 0.20 equiv.), $Cu(OAc)_2$ (0.0125 mmol, 0.05 equiv), MeCN (2.0 mL) were added in a sealed 10-mL reaction vial. Then, the mixture was stirred at 100 °C for 12 h until complete consumption of the starting material **1a** as detected by TLC. The mixture was cooled to room temperature and evaporated under vacuum. The crude mixture was purified by flash column chromatography (petroleum ether /ethyl acetate) to afford the desired product 7-methyl-5-phenyl-11-tosyl-11H-benzo[*b*]fluoren-10-ol (**3a**) in 71% yield.

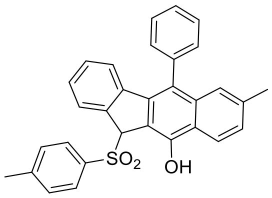


Red solid, mp 188-189 °C; IR (KBr, v, $cm^{-1}$): 3447, 2917, 1625, 1598, 1586, 1507, 1472, 1301. $^1$HNMR (400 MHz, DMSO-$d_6$; $\delta$, ppm) 8.29 (d, $J$ = 8.4 Hz, 1H, ArH), 7.68 (d, $J$ = 7.6 Hz, 1H, ArH), 7.61-7.07 (m, 7H, ArH), 7.07-6.90 (m, 4H, ArH), 6.74 (d, $J$ = 8.4 Hz, 2H, ArH), 6.68-6.56 (m, 1H, ArH), 6.44 (s, 1H, CH), 5.85 (d, $J$ = 8.0 Hz, 1H, OH), 2.33 (s, 3H, $CH_3$), 2.28 (s, 3H, $CH_3$). $^{13}$CNMR (100 MHz, $CDCl_3$; $\delta$, ppm) 150.3, 144.9, 142.2, 138.2, 137.3, 136.4, 135.5, 135.3, 130.3, 130.2, 129.9, 129.4, 129.2, 129.1, 128.9, 128.5, 127.7, 127.5, 127.4, 126.8, 126.7, 125.1, 123.7, 123.5, 122.9, 112.9, 70.9, 22.0, 21.5. HRMS (APCI): m/z Calcd. For: $C_{31}H_{24}O_3S$, 475.1367 $[M-H]^-$, found: 475.1397.

**11-((4-Bromophenyl)sulfonyl)-7-methyl-5-phenyl-11*H*-benzo[*b*]fluoren-10-ol (3b)**

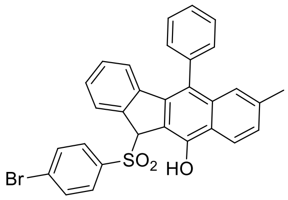


Red solid, mp 233-234 °C; IR (KBr, v, $cm^{-1}$): 3466, 2936, 1689, 1565, 1543, 1511, 1488, 1321. $^1$HNMR (400 MHz, DMSO-$d_6$; $\delta$, ppm) 7.94 (d, $J$ = 7.6 Hz, 1H, ArH), 7.53-7.50 (m, 3H, ArH), 7.39-7.37 (m, 1H, ArH), 7.29 (d, $J$ = 7.6 Hz, 1H, ArH), 7.25- 7.11 (m, 5H, ArH), 7.06-7.02 (m, 1H,

ArH), 6.88 (d, *J* = 8.4 Hz, 2H, ArH), 6.79-6.75 (m, 1H, ArH), 6.01 (d, *J* = 8.0 Hz, 1H, CH), 5.78 (s, 1H, OH), 2.39 (s, 3H, $CH_3$). HRMS (APCI): m/z Calcd. For: $C_{31}H_{24}O_3S$, 541.0296 $[M-H]^-$, found: 541.0281.

**7-Methyl-5-phenyl-11-(phenylsulfonyl)-11*H*-benzo[*b*]fluoren-10-ol (3c)**

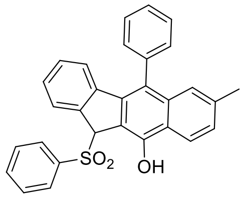


Yellow solid, mp 188-189 °C; IR (KBr, v, $cm^{-1}$): 3448, 2918, 1627, 1613, 1510, 1448, 1302, 1262. $^1$HNMR (400 MHz, $CDCl_3$; $\delta$, ppm) 8.78 (s, 1H, ArH), 8.34 (d, *J* = 8.4 Hz, 1H, ArH), 7.88 (d, *J* = 7.6 Hz, 1H, ArH), 7.51-7.36 (m, 3H, ArH), 7.33-7.29 (m, 2H, ArH), 7.20-7.07 (m, 2H, ArH), 7.06-6.89 (m, 6H, ArH), 6.67 (d, *J* = 7.2 Hz, 1H, ArH), 5.93 (d, *J* = 8.0 Hz, 1H, CH), 5.73 (s, 1H, OH), 2.32 (s, 3H, $CH_3$). $^{13}$CNMR (100 MHz, $CDCl_3$; $\delta$, ppm) 149.2, 141.2, 137.1, 136.3, 135.3, 134.5, 134.1, 132.7, 131.8, 129.2, 129.2, 129.1, 128.4, 128.1, 128.0, 128.0, 126.9, 126.7, 126.5, 126.4, 125.8, 125.7, 124.1, 122.6, 122.4, 121.9, 111.7, 69.8, 20.9. HRMS (APCI): m/z Calcd. For: $C_{30}H_{22}O_3S$, 461.1211 $[M-H]^-$, found: 461.1216.

**11-((4-(Tert-butyl)phenyl)sulfonyl)-7-methyl-5-phenyl-11*H*-benzo[*b*]fluoren-10-ol (3d)**

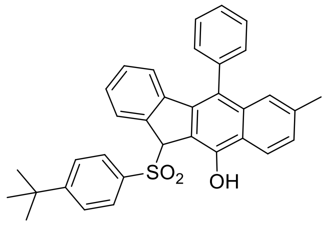


Yellow solid, mp 208-209 °C; IR (KBr, v, $cm^{-1}$): 3436, 2964, 1628, 1593, 1507, 1463, 1398, 1308. $^1$HNMR (400 MHz, $CDCl_3$; $\delta$, ppm) 8.29 (d, *J* = 8.4 Hz, 1H, ArH), 7.72 (d, *J* = 7.6 Hz, 1H, ArH), 7.69-7.29 (m, 5H, ArH), 7.28-7.24 (m, 1H, ArH), 7.22-7.12 (m, 3H, ArH), 7.03-6.94 (m, 2H, ArH), 6.80-6.72 (m, 2H, ArH), 6.66-6.64 (m, 1H, ArH), 6.44 (s, 1H, CH), 5.81 (d, *J* = 8.0 Hz, 1H, OH), 2.33 (s, 3H, CH3), 1.17 (s, 9H, $CH_3$). $^{13}$CNMR (100 MHz, $CDCl_3$; $\delta$, ppm) 157.9, 150.3, 142.2, 138.2, 137.2, 136.5, 135.4, 135.4, 130.3, 130.1, 129.6, 129.4, 129.1, 129.0, 128.8, 127.7, 127.5, 127.4, 126.8, 126.7, 125.2, 124.8, 123.7, 123.4, 122.9, 113.1, 70.8, 35.1, 30.9, 22.0. HRMS (APCI): m/z Calcd. For: $C_{34}H_{30}O_3S$, 517.1837 $[M-H]^-$, found: 517.1855.

**5-(4-Methoxyphenyl)-7-methyl-11-tosyl-11*H*-benzo[*b*]fluoren-10-ol (3e)**

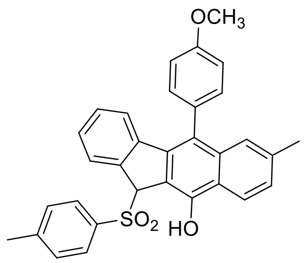


Red solid, mp 209-210 °C; IR (KBr, v, $cm^{-1}$): 3483, 2919, 1646, 1577, 1547, 1518, 1457, 1328. $^1$HNMR (400 MHz, DMSO-*d*$_6$; $\delta$, ppm) 8.33 (d, *J* = 8.4 Hz, 1H, ArH), 7.86 (d, *J* = 7.6 Hz, 1H, ArH), 7.29 (d, *J* = 8.4 Hz, 1H, ArH), 7.19 (s, 1H, ArH), 7.09-7.04 (m, 2H, ArH), 7.00-6.96 (m, 2H, ArH), 6.93-6.88 (m, 3H, ArH), 6.76 (d, *J* = 8.1 Hz, 2H, ArH), 6.59-6.56 (m, 1H, ArH), 6.07 (d, *J* =

7.8 Hz, 1H, CH), 5.71 (s, 1H, OH), 3.86 (s, 3H,$OCH_3$), 2.33 (s, 3H, $CH_3$), 2.18 (s, 3H, $CH_3$). HRMS (APCI): m/z Calcd. For: $C_{32}H_{26}O_4S$, 505.1474 $[M-H]^-$, found: 505.1482.

**5-(4-Chlorophenyl)-7-methyl-11-tosyl-11*H*-benzo[*b*]fluoren-10-ol (3f)**

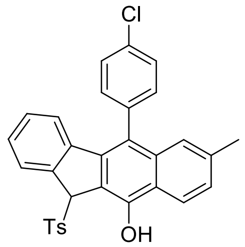


Red solid, mp 194-195 $^oC$; IR (KBr, ν, $cm^{-1}$): 3441, 2972, 1627, 1576, 1560, 1446, 1398, 1321. $^1$HNMR (400 MHz, DMSO-$d_6$; $\delta$, ppm) 8.29 (d, $J$ = 8.4 Hz, 1H, ArH), 7.69 (d, $J$ = 7.6 Hz, 1H, ArH), 7.65-7.56 (m, 2H, ArH), 7.53-7.20 (m, 4H, ArH), 7.11-7.07 (m, 1H, ArH), 7.04-6.86 (m, 3H, ArH), 6.98 (d, $J$ = 8.4 Hz, 2H, ArH), 6.66-6.56 (m, 1H, ArH), 6.45 (s, 1H, CH), 5.94 (d, $J$ = 8.0 Hz, 1H, OH), 2.35 (s, 3H, $CH_3$), 2.27 (s, 3H, $CH_3$). $^{13}$CNMR (100 MHz, $CDCl_3$; $\delta$, ppm) 150.6, 144.9, 141.9, 137.5, 136.7, 136.5, 135.4, 135.3, 133.7, 131.8, 131.7, 129.9, 129.5, 129.4, 129.3, 129.2, 128.6, 127.6, 127.6, 126.9, 125.2, 124.8, 123.7, 123.3, 123.1, 112.9, 70.8, 22.0, 21.5. HRMS (APCI): m/z Calcd. For: $C_{31}H_{23}ClO_3S$, 509.0977 $[M-H]^-$, found: 509.0991.

**7-Methyl-5-(naphthalen-1-yl)-11-tosyl-11*H*-benzo[*b*]fluoren-10-ol (3g)**

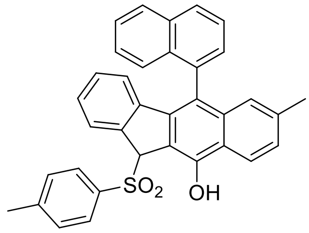


Yellow solid, mp 210-211 $^oC$; IR (KBr, ν, $cm^{-1}$): 3442, 2933, 1663, 1586, 1534, 1478, 1397, 1202. $^1$HNMR (400 MHz, $CDCl_3$; $\delta$, ppm) 9.09 (s, 1H, ArH), 8.46 (d, $J$ = 8.4 Hz, 1H, ArH), 8.00-7.94(m, 3H, ArH), 7.68-7.56 (m, 1H, ArH), 7.46-7.42 (m, 1H, ArH), 7.37 (d, $J$ = 8.4 Hz, 1H, ArH), 7.31 (d, $J$ = 6.8 Hz, 1H, ArH), 7.22-7.19 (m, 1H, ArH), 7.05-7.00 (m, 3H, ArH), 6.95-6.75 (m, 5H, ArH), 6.54 (d, $J$ = 8.4 Hz, 1H, ArH), 5.85 (s, 1H, CH), 5.63 (d, $J$ = 8.0 Hz, 1H, OH), 2.29 (s, 3H, $CH_3$), 2.27 (s, 3H, $CH_3$). $^{13}$CNMR (100 MHz, $CDCl_3$; $\delta$, ppm) 150.6, 144.8, 141.8, 137.4, 136.1, 135.7, 135.2, 133.8, 132.8, 130.2, 129.5, 129.2, 128.7, 128.3, 128.2, 128.2, 127. 7, 127.4, 126.7, 126.1, 125.9, 125.7, 125.3, 125.2, 124.0, 123.8, 123.4, 122.9, 112.6, 70.8, 21.9, 21.8. HRMS (APCI): m/z Calcd. For: $C_{35}H_{25}O_3S$, 525.1524 $[M-H]^-$, found: 525.1542.

**7-Bromo-5-(4-methoxyphenyl)-11-tosyl-11*H*-benzo[*b*]fluoren-10-ol (3h)**

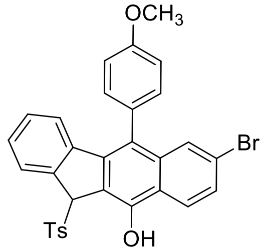


Yellow solid, mp 218-219 $^oC$; IR (KBr, ν, $cm^{-1}$): 3446, 2943, 622, 581, 1511, 1426, 1363, 1243. $^1$HNMR (400 MHz, $CDCl_3$; $\delta$, ppm) 8.36 (d, $J$ = 8.8 Hz, 1H, ArH), 7.93 (d, $J$ = 7.6 Hz, 1H, ArH), 7.60-7.57 (m, 1H, ArH), 7.52 (d, $J$ = 1.6 Hz, 1H, ArH), 7.32-7.28 (m, 1H, ArH), 7.16-7.07 (m, 2H,

ArH), 7.07-7.05 (m, 2H, ArH), 7.02-6.92 (m, 3H, ArH), 6.84 (d, *J* = 8.0 Hz, 2H) , ArH, 6.63-6.60 (m, 1H, ArH), 6.16 (d, *J* = 8.0 Hz, 1H,CH), 5.76 (s, 1H,OH), 3.93 (s, 3H,$OCH_3$), 2.26 (s, 3H,$CH_3$). $^{13}$CNMR (100 MHz, $CDCl_3$; $\delta$, ppm) 159.4, 150.2, 145.1, 141.9, 137.9, 136.9, 135.3, 131.3, 131.1, 129.7, 129.6, 129.2, 129.1, 128.6, 128.2, 127.9, 126.8, 126.0, 124.9, 124.0, 123.8, 122.2, 114.7, 114.6, 114.2, 70.9, 55.4, 21.5. HRMS (APCI): m/z Calcd. For: $C_{31}H_{23}BrO_4S$, 569.0421 $[M-H]^-$, found: 569.0428.

**7-Bromo-5-(p-tolyl)-11-tosyl-11*H*-benzo[*b*]fluoren-10-ol (3i)**

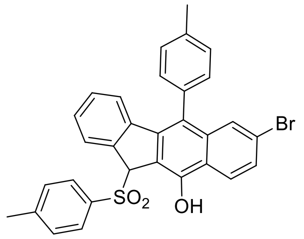


Red solid, mp 176-177 °C; IR (KBr, v, $cm^{-1}$): 3469, 2920, 1621, 1577, 1418, 1354, 1289, 1232. $^1$HNMR (400 MHz, $CDCl_3$; $\delta$, ppm) 8.36 (d, *J* = 8.8 Hz, 1H, ArH), 7.93 (d, *J* = 7.6 Hz, 1H, ArH), 7.60-7.57 (m, 1H, ArH), 7.51 (s, 1H, ArH), 7.37-7.26 (m, 3H, ArH), 7.24 (d, *J* = 6.4 Hz, 1H, ArH), 7.10-7.04 (m, 2H, ArH), 6.94 (d, *J* = 8.0 Hz, 2H, ArH), 6.84 (d, *J* = 8.0 Hz, 2H, ArH), 6.57 (d, *J* = 8.0 Hz, 1H, ArH), 6.11 (d, *J* = 8.0 Hz, 1H, CH), 5.77 (s, 1H, OH), 2.49 (s, 3H, $CH_3$), 2.26 (s, 3H, $CH_3$). $^{13}$CNMR (100 MHz, $CDCl_3$; $\delta$, ppm) 150.3, 144.9, 142.2, 138.2, 137.2, 136.4, 135.4, 135.3, 130.3, 130.2, 129.8, 129.4, 129.1, 129.0, 128.9, 128.5, 127.7, 127.5, 127.4, 126.8, 126.7, 125.1, 123.6, 123.5, 122.9, 70.9, 21.3, 21.5. HRMS (APCI): m/z Calcd. For: $C_{31}H_{23}BrO_3S$, 553.0472 $[M-H]^-$, found: 553.0490.

**7-Bromo-5-phenyl-11-tosyl-11*H*-benzo[*b*]fluoren-10-ol (3j)**

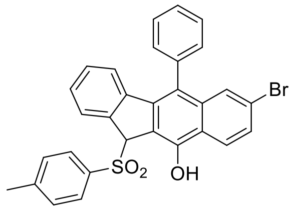


Red solid, mp 200-201 °C; IR (KBr, v, $cm^{-1}$): 3443, 3024, 1623, 1581, 1490, 1447, 1415, 1305, 1215. $^1$HNMR (400 MHz, $CDCl_3$; $\delta$, ppm) 8.37 (d, *J* = 8.8 Hz, 1H, ArH), 7.93 (d, *J* = 7.6 Hz, 1H, ArH), 7.65-7.40 (m, 5H, ArH), 7.30 (d, *J* = 7.6 Hz, 1H, ArH), 7.23-7.16 (m, 1H, ArH), 7.03 (m, 1H, ArH), 6.95 (d, *J* = 8.4 Hz, 2H, ArH), 6.85 (d, *J* = 8.0 Hz, 2H, ArH), 6.70 (d, *J* = 7.2 Hz, 1H, ArH), 6.03 (d, *J* = 8.0 Hz, 1H, CH), 5.77 (s, 1H, OH), 2.27 (s, 3H, $CH_3$). $^{13}$CNMR (100 MHz, $CDCl_3$; $\delta$, ppm) 150.3, 145.2, 141.7, 137.6, 137.18, 136.5, 135.4, 130.2, 130.1, 129.7, 129.6, 129.3, 129.1, 129.1, 128.7, 128.6, 128.2, 127.9, 126.8, 126.4, 124.9, 123.9, 123.7, 122.3, 114.2, 70.8, 21.5. HRMS (APCI): m/z Calcd. For: $C_{30}H_{21}BrO_3S$, 539.0316 $[M-H]^-$, found: 539.0343.

**5-Phenyl-11-tosyl-11*H*-benzo[*b*]fluoren-10-ol (3k)**

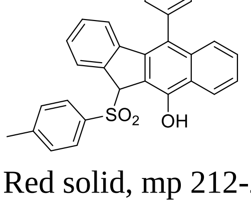


Red solid, mp 212-213 °C; IR (KBr, v, $cm^{-1}$): 3457, 3011, 1624, 1596, 1443, 1422, 1380, 1283.

$^{1}$HNMR (400 MHz, $CDCl_3$; $\delta$, ppm) 8.52 (d, $J$ = 8.4 Hz, 1H, ArH), 7.93 (d, $J$ = 7.6 Hz, 1H, ArH), 7.57-7.39 (m, 6H, ArH), 7.36 (d, $J$ = 8.4 Hz, 1H, ArH), 7.28 (d, $J$ = 7.6 Hz, 1H, ArH), 7.24-7.20 (m, 1H, ArH), 7.07-6.92 (m, 3H, ArH), 6.83 (d, $J$ = 8.0 Hz, 2H, ArH), 6.75 (d, $J$ = 7.2 Hz, 1H, ArH), 6.07 (d, $J$ = 8.0 Hz, 1H, CH), 5.80 (s, 1H, OH), 2.25 (s, 3H, $CH_3$). $^{13}$CNMR (100 MHz, $CDCl_3$; $\delta$, ppm) 150.3, 145.0, 142.2, 138.1, 136.3, 135.3, 135.2, 130.3, 130.2, 129.9, 129.5, 129.2, 129.1, 129.0, 128.6, 127.8, 127.5, 127.3, 127.3, 126.8, 126.1, 125.5, 125.3, 123.5, 123.1, 113.7, 70.9, 21.5. HRMS (APCI): m/z Calcd. For: $C_{30}H_{22}O_3S$, 461.1211 $[M-H]^-$, found: 461.1219.

**7-Bromo-5-phenyl-11-(phenylsulfonyl)-11*H*-benzo[*b*]fluoren-10-ol (3l)**

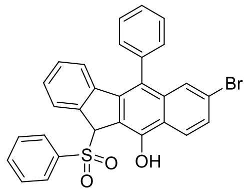


Yellow solid, mp 194-195 °C; IR (KBr, v, cm$^{-1}$): 3449, 2953, 1644, 1578, 1545, 1483, 1324, 1298, 1206. $^{1}$HNMR (400 MHz, $CDCl_3$;$\delta$, ppm) 8.38 (d, $J$ = 8.8 Hz, 1H, ArH), 7.95 (d, $J$ = 7.6 Hz, 1H, ArH), 7.61-7.58 (m, 1H, ArH), 7.55-7.50 (m, 2H, ArH), 7.49-7.28 (m, 5H, ArH), 7.20-7.18 (m, 1H, ArH), 7.11-7.00 (m, 5H, ArH), 6.72 (d, $J$ = 7.2 Hz, 1H, ArH), 6.02 (d, $J$ = 8.0 Hz, 1H, CH), 5.79 (s, 1H, OH). $^{13}$CNMR (100 MHz, $CDCl_3$;$\delta$, ppm) 150.3, 141.7, 137.5, 137.1, 136.5, 135.2, 133.9, 132.7, 130.2, 130.0, 129.6, 129.3, 129.2, 129.1, 128.7, 128.2, 128.2, 128.0, 128.0, 127.0, 126.5, 124.9, 124.0, 123.7, 122.3, 114.0, 70.8. HRMS (APCI): m/z Calcd. For: $C_{29}H_{19}BrO_3S$, 525.0159 $[M-H]^-$, found: 525.0163.

**5-(4-Chlorophenyl)-11-(phenylsulfonyl)-11*H*-benzo[*b*]fluoren-10-ol (3m)**

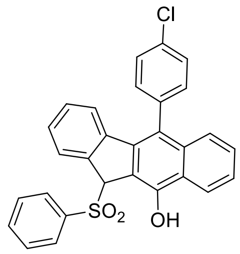


Yellow solid, mp 192-193 °C; IR (KBr, v, cm$^{-1}$): 3444, 2978, 1682, 1598, 1455, 1378, 1289, 1211. $^{1}$HNMR (400 MHz, $CDCl_3$;$\delta$, ppm) 8.51 (d, $J$ = 8.0 Hz, 1H, ArH), 7.88 (d, $J$ = 1.2 Hz, 1H, ArH), 7.56-7.48 (m, 3H, ArH), 7.47-7.40 (m, 2H, ArH), 7.37-7.34 (m, 1H, ArH), 7.21-7.19 (m, 1H, ArH), 7.05-6.94 (m, 3H, ArH), 6.88 (d, $J$ = 8.0 Hz, 2H, ArH), 6.76-6.67 (m, 1H, ArH), 5.96 (d, $J$ = 8.4 Hz, 1H, CH), 5.74 (s, 1H, OH), 2.27 (s, 3H, $CH_3$). $^{13}$CNMR (100 MHz, $CDCl_3$; $\delta$, ppm) 150.3, 145.3, 140.6, 137.7, 137.0, 135.2, 133.3, 130.1, 130.0, 129.7, 129.7, 129.2, 129.1, 129.0, 128.7, 128.0, 127.5, 127.4, 126.7, 126.1, 125.6, 124.3, 123.1, 113.4, 70.6, 21.5. HRMS (APCI): m/z Calcd. For: $C_{29}H_{19}ClO_3S$, 481.0664 $[M-H]^-$, found: 481.0681.

**13-Phenyl-8-tosyl-8*H*-indeno[2,1-*b*]phenanthren-7-ol (3n)**

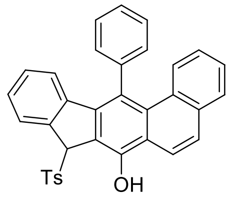


Yellow solid, mp 156-157 °C; IR (KBr, v, cm$^{-1}$): 3445, 2911, 1668, 1599, 1538, 1505, 1373, 1302.

$^{1}$HNMR (400 MHz, CDCl$_3$; $\delta$, ppm) 8.77 (s, 1H, ArH), 8.47 (d, $J$ = 8.8 Hz, 1H, ArH), 7.87 (s, 1H, ArH), 7.81-7.65 (m, 2H, ArH), 7.56-7.40 (m, 4H, ArH), 7.40-7.34 (m, 1H, ArH), 7.18-7.10 (m, 2H, ArH), 6.97 (m 1H, ArH), 6.89 (m, 3H, ArH), 6.77 (m, 3H, ArH), 5.71 (s, 1H, CH), 5.55 (d, $J$ = 8.0 Hz, 1H, OH), 2.17 (s, 3H, CH$_3$). $^{13}$CNMR (100 MHz, CDCl$_3$; $\delta$, ppm) 149.4, 144.3, 141.1, 137.0, 136.9, 135.9, 134.6, 133.2, 129.3, 129.3, 129.2, 129.2, 129.1, 129.1, 128.9, 128.8, 128.3, 127.8, 127.8, 127.4, 127.1, 126.0, 125.7, 124.1, 123.2, 123.0, 121.4, 113.5, 70.1, 20.7. HRMS (APCI):m/z Calcd. For: $C_{34}H_{24}O_3S$, 511.1367 [M-H]$^-$, found: 511.1375.

**Preparation of Compound 5a: 1-(4-Chlorophenyl)-3-phenyl-3-tosylpropan-1-one**

A mixture of 1-(4-chlorophenyl)-3-phenylprop-2-en-1-one (**4a**, 0.25 mmol, 1.0 equiv.), tosylhydrazide (**2a**, 0.5 mmol, 2.0 equiv.), BPO (0.50 mmol, 2.0 equiv.), TBHP (0.25 mol, 1.0 equiv., 70% in water), TBAI (0.05 mmol, 0.2 equiv.), MeCN (2.5 mL) were added in a sealed 10-mL reaction vial. Then, the mixture was stirred in oil bath at 100 °C for 6 h until complete consumption of the starting material **4a** as detected by TLC. The mixture was cooled to room temperature and evaporated under vacuum. The crude mixture was purified by flash column chromatography (petroleum ether /ethyl acetate) to afford the desired product **5a** in 77% yield.

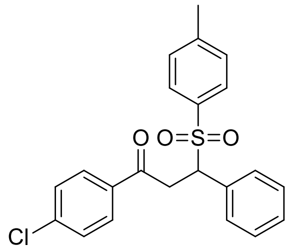


White solid, mp 199-201 °C; IR (KBr, ν, cm$^{-1}$): 2949, 2921, 1689, 1589, 1489, 1311, 1229, 1142, 1086. $^{1}$HNMR (400 MHz, CDCl$_3$; $\delta$, ppm) 7.81 (d, $J$ = 8.4 Hz, 2H, ArH), 7.42-7.28 (m, 4H, ArH), 7.19-7.05 (m, 7H, ArH), 4.83-4.77 (m, 1H, CH), 4.01 (d, $J$ = 3.6 Hz, 1H, CH$_2$), 3.82 (d, $J$ = 9.6 Hz, 1H, CH$_2$), 2.32 (s, 3H, CH$_3$). $^{13}$CNMR (100 MHz, CDCl$_3$; $\delta$, ppm) 193.9, 144. 8, 140.2, 134.5, 133.9, 132.6, 129.7, 129.5, 129.4, 129.1, 128.8, 128.5, 66.4, 37.0, 21.6. HRMS (ESI): m/z Calcd. For: $C_{22}H_{19}ClO_3S$, 399.0821 [M+H]$^+$, found: 399.0813.

**1-(4-Chlorophenyl)-3-((4-methoxyphenyl)sulfonyl)-3-phenylpropan-1-one (5b)**

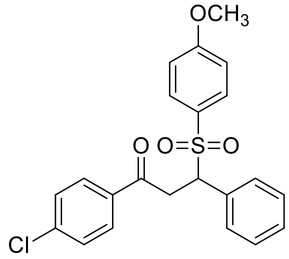


White solid, mp 182-183 °C; IR (KBr, ν, cm$^{-1}$): 3060, 2920, 1689, 1595, 1497, 1314, 1258, 1139, 1089. $^{1}$HNMR (400 MHz, CDCl$_3$; $\delta$, ppm) 7.95-7.83 (m, 2H, ArH), 7.43 (d, $J$ = 8.4 Hz, 4H, ArH), 7.25-7.14 (m, 5H, ArH), 6.83 (d, $J$ = 8.8 Hz, 2H, ArH), 4.88-4.85 (m, 1H, CH), 4.15-4.03 (m, 1H, CH$_2$), 3.94-3.86 (m, 1H, CH$_2$), 3.83 (s, 3H, OCH$_3$). $^{13}$CNMR (100 MHz, CDCl$_3$; $\delta$, ppm) 193.9, 163.8, 140.2, 134.5, 133.9, 132.7, 131.2, 129.7, 129.6, 129.1, 128.8, 128.5, 113.9, 66.6, 55.6, 37.0. HRMS (ESI): m/z Calcd. For: $C_{22}H_{19}ClO_4S$, 415.0771 [M+H]$^+$, found: 415.0774.

**1-(4-Chlorophenyl)-3-phenyl-3-(phenylsulfonyl)propan-1-one (5c)**

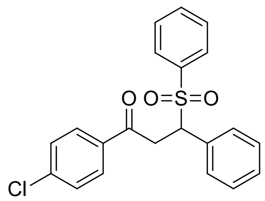


White solid, mp 190-192 °C; IR (KBr, ν, cm$^{-1}$): 3063, 2921, 1690, 1588, 1447, 1306, 1226, 1139, 1085. $^{1}$HNMR (400 MHz, $CDCl_3$; *δ*, ppm) 7.95-7.85 (m, 2H, ArH), 7.58-7.53 (m, 3H, ArH), 7.45-7.37 (m, 4H, ArH), 7.26-7.16 (m, 5H, ArH), 4.93-4.89 (m, 1H, CH), 4.15-4.09 (m, 1H, $CH_2$), 3.92-3.85 (m, 1H, $CH_2$). $^{13}$CNMR (100 MHz, $CDCl_3$; *δ*, ppm) 193.8, 140.3, 136.8, 134.4, 133.8, 132.4, 129.7, 129.6, 129.1, 129.0, 128.9, 128.7, 128.5, 66.4, 36.9. HRMS (ESI): m/z Calcd. For: $C_{21}H_{17}ClO_3S$, 385.0665 [M+H]$^{+}$, found: 385.0650.

**3-(4-Methoxyphenyl)-1-phenyl-3-tosylpropan-1-one (5d)**

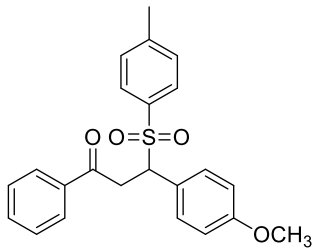


White solid, mp 186-187 °C; IR (KBr, ν, cm$^{-1}$): 3071, 2938, 1670, 1601, 1498, 1310, 1243, 1145, 1087. $^{1}$HNMR (400 MHz, $CDCl_3$; *δ*, ppm) 7.95-7.87 (m, 2H, ArH), 7.42 (d, *J* = 8.4 Hz, 2H, ArH), 7.24-7.20 (m, 7H, ArH), 6.98-6.88 (m, 2H, ArH), 4.92-4.89 (m, 1H, CH), 4.09-4.01 (m, 1H, $CH_2$), 3.91 (d, *J* = 9.6 Hz, 1H, $CH_2$), 3.86 (s, 3H, $OCH_3$), 2.38 (s, 3H, $CH_3$). $^{13}$CNMR (100 MHz, $CDCl_3$; *δ*, ppm) 193.4, 163.9, 144.7, 134.1, 132.7, 130.5, 129.8, 129.4, 129.3, 129.0, 128.7, 128.4, 113.9, 66.6, 55.5, 36.6, 21.6. HRMS (ESI): m/z Clcd. For: $C_{23}H_{22}O_4S$, 395.1317 [M+H]$^{+}$, found: 395.1318.

**3-(4-Methoxyphenyl)-3-((4-methoxyphenyl)sulfonyl)-1-phenylpropan-1-one (5e)**

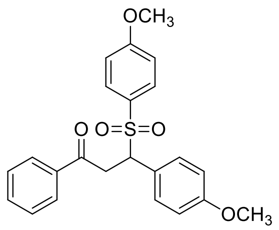


White solid, mp 181-182 °C; IR (KBr, ν, cm$^{-1}$): 3061, 2920, 1671, 1598, 1499, 1305, 1238, 1138, 1088. $^{1}$HNMR (400 MHz, $CDCl_3$; *δ*, ppm) 7.92 (d, *J* = 8.8 Hz, 2H, ArH), 7.48-7.36 (m, 2H, ArH), 7.24-7.11 (m, 5H, ArH), 6.92 (d, *J* = 8.8 Hz, 2H, ArH), 6.82 (d, *J* = 8.8 Hz, 2H, ArH), 4.93 (s, 1H, CH), 4.08-4.00 (m, 1H, $CH_2$), 3.90 (d, *J* = 9.6 Hz, 1H, $CH_2$), 3.86 (s, 3H, $OCH_3$), 3.83 (s, 3H, $OCH_3$). $^{13}$CNMR (100 MHz, $CDCl_3$; *δ*, ppm) 193.4, 163.9, 163.7, 132.9, 131.2, 130.5, 129. 8, 129.3, 128.7, 128.5, 128.4, 113.9, 113.9, 66.8, 55.6, 55.5, 36.6. HRMS (ESI): m/z Calcd. For: $C_{23}H_{22}O_5S$, 411.1266 [M+H]$^{+}$, found: 411.1262.

**3-(4-Methoxyphenyl)-1-phenyl-3-(phenylsulfonyl)propan-1-one (5f)**

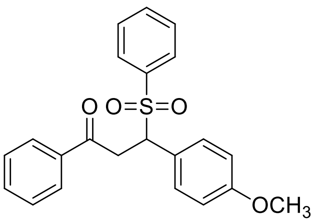


White solid, mp 178-179 °C; IR (KBr, ν, cm$^{-1}$): 3060, 2939, 1671, 1598, 1499, 1306, 1256, 1138, 1089. $^{1}$HNMR (400 MHz, $CDCl_3$; *δ*, ppm) 7.95 (d, *J* = 8.8 Hz, 2H, ArH), 7.61-7.52 (m, 3H, ArH), 7.41 (d, *J* = 8.0 Hz, 2H, ArH), 7.26-7.20 (m, 5H, ArH), 6.94 (d, *J* = 8.8 Hz, 2H, ArH ), 4.97-4.94 (m, 1H, CH), 4.12-4.06 (m, 1H, $CH_2$), 3.98-3.90 (m, 1H, $CH_2$), 3.88 (s, 3H, $CH_3$). $^{13}$CNMR (100 MHz, $CDCl_3$; *δ*, ppm) 193.3, 163.9, 137.0, 133.7, 132.6, 130.5, 129.8, 129.2, 129.0, 128.758, 128.7, 128.4, 113.9, 66.6, 55.5, 36.4. HRMS (ESI): m/z Calcd. For: $C_{22}H_{20}O_4S$, 381.1160 [M+H]$^+$, found: 381.1161.

**3-(4-Bromophenyl)-1-(m-tolyl)-3-tosylpropan-1-one (5g)**

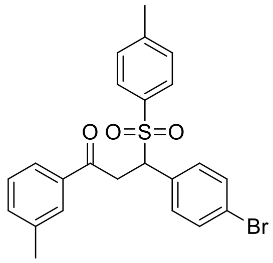


White solid, mp 181-182 °C; IR (KBr, ν, cm$^{-1}$): 3073, 2939, 1688, 1595, 1486, 1302, 1286, 1084. $^{1}$HNMR (400 MHz, $CDCl_3$; *δ*, ppm) 7.74-7.70 (m, 2H, ArH), 7.45 (d, *J* = 8.4 Hz, 2H, ArH), 7.40-7.32 (m, 4H, ArH), 7.22 (d, *J* = 8.0 Hz, 2H, ArH), 7.11-7.07 (m, 2H, ArH), 4.87-4.84 (m, 1H, CH), 4.10-4.05 (m, 1H, $CH_2$), 3.91-3.84 (m, 1H, $CH_2$), 2.40 (d, *J* = 2.8 Hz, 6H, $CH_3$). $^{13}$CNMR (100 MHz, $CDCl_3$; *δ*, ppm) 194.9, 145.0, 138.6, 136.0, 134.5, 133.8, 131.8, 131.6, 131.3, 129.6, 129.0, 128.6, 128.6, 125.3, 123.0, 65.9, 37.0, 21.7, 21.3. HRMS (ESI): m/z Calcd. For: $C_{23}H_{21}BrO_3S$, 457.0473 [M+H]$^+$, found: 457.0465.

**3-(4-Bromophenyl)-3-((4-methoxyphenyl)sulfonyl)-1-(m-tolyl)propan-1-one (5h)**

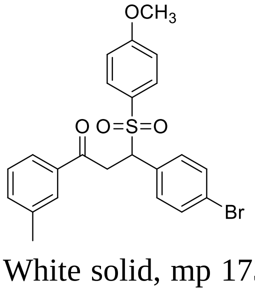


White solid, mp 173-175 °C; IR (KBr, ν, cm$^{-1}$): 3079, 2941, 1685, 1596, 1498, 1298, 1261, 1147, 1086, 1008. $^{1}$HNMR (400 MHz, $CDCl_3$; *δ*, ppm) 7.72 (s, 2H, ArH), 7.48 (d, *J* = 8.8 Hz, 2H, ArH), 7.40-7.32 (m, 4H, ArH), 7.09 (d, *J* = 8.4 Hz, 2H, ArH), 6.87 (d, *J* = 8.8 Hz, 2H, ArH), 4.86-4.81 (m, 1H, CH), 4.06 (d, *J* = 3.2 Hz, 1H, $CH_2$), 3.94-3.87 (m, 1H, $CH_2$), 3.85 (s, 3H, $OCH_3$), 2.40 (s, 3H, $CH_3$). $^{13}$CNMR (100 MHz, $CDCl_3$; *δ*, ppm) 195.0, 163.9, 138.7, 136.0, 134.6, 133.9, 132.0, 131.7, 131.3, 131.2, 128.7, 128.2, 125.3, 123.0, 114.1, 66.1, 55.7, 37.1, 21.3. HRMS (ESI): m/z Calcd. For: $C_{23}H_{21}BrO_4S$, 473.0422 [M+H]$^+$, found: 473.0440.

**3-(4-Bromophenyl)-3-(phenylsulfonyl)-1-(*m*-tolyl)propan-1-one (5i)**

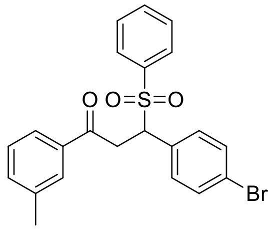


White solid, mp 174-175 °C; IR (KBr, ν, $cm^{-1}$): 3064, 2949, 1684, 1585, 1490, 1307, 1255, 1142, 1085. $^{1}$HNMR (400 MHz, $CDCl_3$; $\delta$, ppm) 7.75-7.70 (m, 2H, ArH), 7.62-7.56 (m, 3H, ArH), 7.46-7.31 (m, 6H, ArH), 7.07 (d, $J$ = 8.4 Hz, 2H, ArH), 4.90-4.86 (m, 1H, CH), 4.12-4.07 (m, 1H, $CH_2$), 3.94-3.83 (m, 1H, $CH_2$), 2.40 (s, 3H, $CH_3$). $^{13}$CNMR (100 MHz, $CDCl_3$; $\delta$, ppm) 194.8, 138.7, 136.8, 136.0, 134.6, 133.9, 131.7, 131.7, 131.3, 129.0, 128.9, 128.7, 128.7, 125.3, 123.1, 65.9, 36.9, 21.3. HRMS (ESI): m/z Calcd. For: $C_{22}H_{19}BrO_3S$, 443.0316 $[M+H]^+$, found: 443.0327.

**3-(4-Bromophenyl)-1-(naphthalen-2-yl)-3-tosylpropan-1-one (5j)**

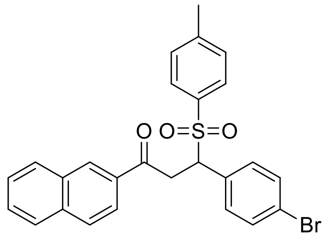


White solid, mp 179-180 °C; IR (KBr, ν, $cm^{-1}$): 3068, 2924, 1679, 1598, 1490, 1303, 1256, 1142, 1089. $^{1}$HNMR (400 MHz, $CDCl_3$; $\delta$, ppm) 8.49 (s, 1H, ArH), 7.99 (d, $J$ = 8.0 Hz, 1H, ArH), 7.93 (d, $J$ = 8.4 Hz, 1H, ArH), 7.87 (d, $J$ = 8.8 Hz, 2H, ArH), 7.65-7.58 (m, 2H, ArH), 7.48 (d, $J$ = 8.0 Hz, 2H, ArH), 7.35 (d, $J$ = 8.4 Hz, 2H, ArH), 7.23 (d, $J$ = 8.0 Hz, 2H, ArH), 7.13 (d, $J$ = 8.4 Hz, 2H, ArH), 4.94-4.89 (m, 1H, CH), 4.26-7.21 (m, 1H, $CH_2$), 4.07-4.00 (m, 1H, $CH_2$), 2.41 (s, 3H, $CH_3$). $^{13}$CNMR (100 MHz, $CDCl_3$; $\delta$, ppm) 194.6, 145.1, 135.8, 133.8, 133.3, 132.4, 131.8, 131.7, 131.4, 130.2, 129. 7, 129.6, 129.0, 128.9, 128.7, 127.8, 127.1, 123.5, 123.1, 66.0, 37.0, 21.7. HRMS (ESI): m/z Calcd. For: $C_{25}H_{19}BrO_3S$, 493.0473 $[M+H]^+$, found: 493.0482.

**3-(4-Bromophenyl)-3-((4-methoxyphenyl)sulfonyl)-1-(naphthalen-2-yl)propan-1-one (5k)**

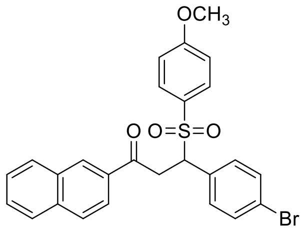


White solid, mp 196-197 °C; IR (KBr, ν, $cm^{-1}$): 3059, 2923, 1682, 1594, 1497, 1315, 1259, 1141, 1090. $^{1}$HNMR (400 MHz, $CDCl_3$; $\delta$, ppm) 8.49 (s, 1H, ArH), 7.99 (d, $J$ = 8.0 Hz, 1H, ArH), 7.93 (d, $J$ = 8.8 Hz, 1H, ArH), 7.87 (d, $J$ = 8.4 Hz, 2H, ArH), 7.64-7.56 (m, 2H, ArH), 7.51 (d, $J$ = 8.4 Hz, 2H, ArH), 7.36 (d, $J$ = 8.0 Hz, 2H, ArH), 7.13 (d, $J$ = 8.4 Hz, 2H, ArH), 6.88 (d, $J$ = 8.8 Hz, 2H, ArH), 4.92-4.87 (m, 1H, CH), 4.22 (d, $J$ = 3.2 Hz, 1H, $CH_2$), 4.05 (d, $J$ = 10.0 Hz, 1H, $CH_2$), 3.85 (s, 3H, $OCH_3$). $^{13}$CNMR (100 MHz, $CDCl_3$; $\delta$, ppm) 194.5, 144.5, 137.4, 137.1, 136.7, 133.7, 133.6, 131.0, 130.0, 129.5, 129.4, 129.1, 128.7, 128.3, 127.0, 66.2, 36.8, 21.7, 19.7, 19.5. HRMS (ESI): m/z Calcd. For: $C_{26}H_{21}BrO_4S$, 509.0422 $[M+H]^+$, found: 509.0426.

**3-(4-Bromophenyl)-1-(naphthalen-2-yl)-3-(phenylsulfonyl)propan-1-one (5l)**

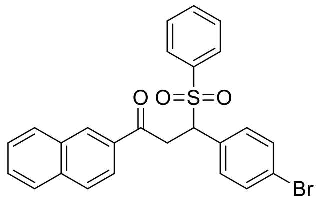


White solid, mp 191-192 °C; IR (KBr, ν, cm$^{-1}$): 3065, 2923, 1675, 1589, 1490, 1307, 1225, 1123, 1086. $^1$HNMR (400 MHz, $CDCl_3$; *δ*, ppm) 8.50 (s, 1H, ArH), 7.99 (d, *J* = 7.6 Hz, 1H, ArH), 7.95-7.92 (m, 1H, ArH), 7.88 (d, *J* = 8.8Hz, 2H, ArH), 7.65-7.57 (m, 5H, ArH), 7.46-7.42 (m, 2H, ArH), 7.35 (d, *J* = 8.4 Hz, 2H, ArH), 7.11 (d, *J* = 8.4 Hz, 2H, ArH), 4.96-4.92 (m, 1H, CH), 4.29-4.23(m, 1H, $CH_2$), 4.09-4.02 (m, 1H, $CH_2$). $^{13}$CNMR (100 MHz, $CDCl_3$; *δ*, ppm) 194.5, 136.8, 135.9, 134.0, 133.3, 132.4, 131.7, 131.3, 130.2, 129.7, 129.0, 128.7, 127.8, 127.1, 123.5, 123.2, 66.1, 36.8. HRMS (ESI): m/z Calcd. For: $C_{25}H_{19}BrO_3S$, 481.0290 $[M+H]^+$, found: 481.0302.

**1,3-Bis(4-bromophenyl)-3-tosylpropan-1-one (5m)**

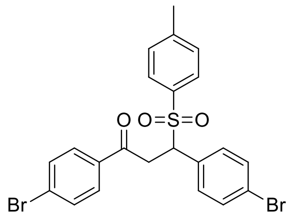


White solid, mp 203-204 °C; IR (KBr, ν, cm$^{-1}$): 3068, 2922, 1689, 1584, 1491, 1299, 1227, 1137, 1010. $^1$HNMR (400 MHz, $CDCl_3$; *δ*, ppm) 7.79 (d, *J* = 8.4 Hz, 2H, ArH), 7.61 (d, *J* = 8.4 Hz, 2H, ArH), 7.44 (d, *J* = 8.4 Hz, 2H, ArH), 7.35 (d, *J* = 8.4 Hz, 2H, ArH), 7.22 (d, *J* = 8.0 Hz, 2H, ArH), 7.07 (d, *J* = 8.4 Hz, 2H, ArH), 4.84-4.81(m, 1H, CH), 4.05 (d, *J* = 3.6 Hz, 1H, $CH_2$), 3.83 (d, *J* = 10.0 Hz, 1H, $CH_2$), 2.41 (s, 3H, $CH_3$). $^{13}$CNMR (100 MHz, $CDCl_3$; *δ*, ppm) 193.8, 145.1, 134.7, 133.6, 132.2, 131.7, 131.3, 129.6, 129.2, 129.0, 123.2, 65.8, 37.0, 21.7. HRMS (ESI): m/z Calcd. For: $C_{22}H_{18}Br_2O_3S$, 522.9396 $[M+H]^+$, found: 522.9390.

**1,3-Bis(4-bromophenyl)-3-((4-methoxyphenyl)sulfonyl)propan-1-one (5n)**

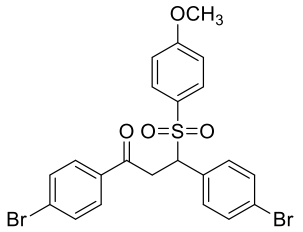


White solid, mp 210-211 °C; IR (KBr, ν, cm$^{-1}$): 3093, 2921, 1687, 1595, 1495, 1261, 1226, 1136, 1087. $^1$HNMR (400 MHz, $CDCl_3$; *δ*, ppm) 7.79 (d, *J* = 8.4 Hz, 2H, ArH), 7.61 (d, *J* = 8.4 Hz, 2H, ArH), 7.47 (d, *J* = 8.8 Hz, 2H, ArH), 7.35 (d, *J* = 8.4 Hz, 2H, ArH), 7.07 (d, *J* = 8.4 Hz, 2H, ArH), 6.87 (d, *J* = 8.8 Hz, 2H, ArH), 4.81 (d, *J* = 6.0 Hz, 1H, CH), 4.10-4.05 (m, 1H, $CH_2$), 3.85 (s, 3H, $OCH_3$), 3.82 (d, *J* = 10,0 Hz, 1H, $CH_2$). $^{13}$CNMR (100 MHz, $CDCl_3$; *δ*, ppm) 193.9, 163.9, 134.7, 132.2, 131.9, 131.7, 131.3, 131.2, 129.6, 129.2, 128.0, 123.2, 114.2, 66.0, 55.7, 37.0. HRMS (ESI): m/z Calcd. For: $C_{22}H_{18}Br_2O_3S$, 538.9345 $[M+H]^+$, found: 538.9329.

**1,3-Bis(4-bromophenyl)-3-(phenylsulfonyl)propan-1-one (5o)**

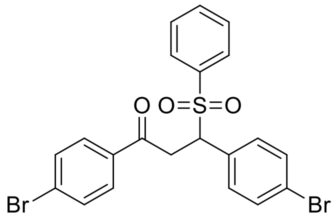


White solid, mp 180-181 $^{o}$C; IR (KBr, ν, cm$^{-1}$): 3059, 2919, 1686, 1584, 1489, 1307, 1227, 1142, 1082. $^{1}$HNMR (400 MHz, $CDCl_3$; *δ*, ppm) 7.79 (d, *J* = 8.4 Hz, 2H, ArH), 7.63-7.55 (m, 5H, ArH), 7.45-7.41 (m, 2H, ArH), 7.34 (d, *J* = 8.4 Hz, 2H, ArH), 7.06 (d, *J* = 8.4 Hz, 2H, ArH), 4.88-4.83 (m, 1H, CH), 4.13-4.06 (m, 1H, $CH_2$), 3.85 (d, *J* = 10.0 Hz, 1H, $CH_2$). $^{13}$CNMR (100 MHz, $CDCl_3$; *δ*, ppm) 193.7, 136.6, 134.7, 134.0, 132.2, 131.7, 131.6, 131.2, 129.6, 129.2, 129.0, 123.3, 65.8, 36.8. HRMS (ESI): m/z Calcd. For: $C_{21}H_{16}Br_2O_3S$, 508.9240 $[M+H]^+$, found: 508.9246.

## Copies of ¹H NMR and ¹³C NMR of Compounds 3 and 5

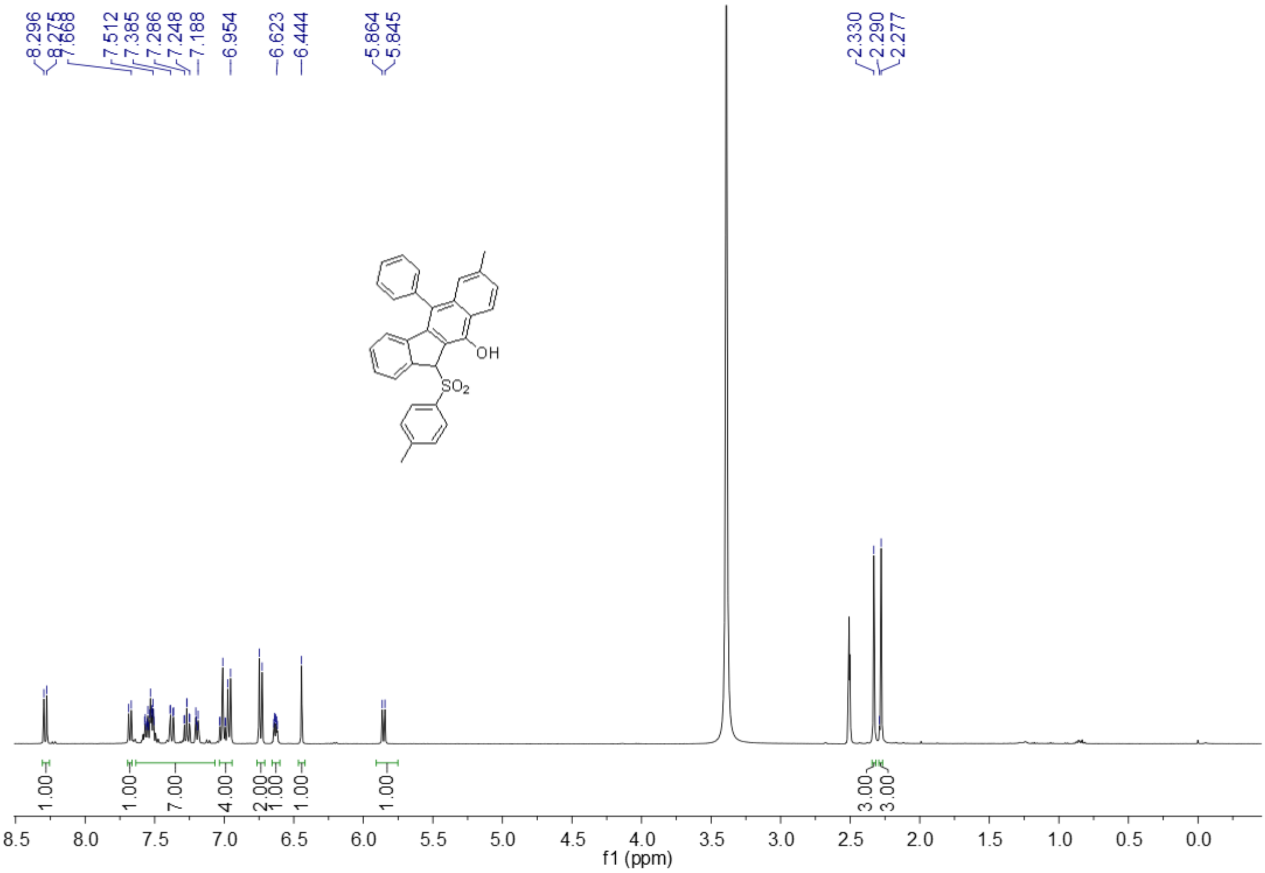


**¹H NMR Spectrum of Compound 3a**

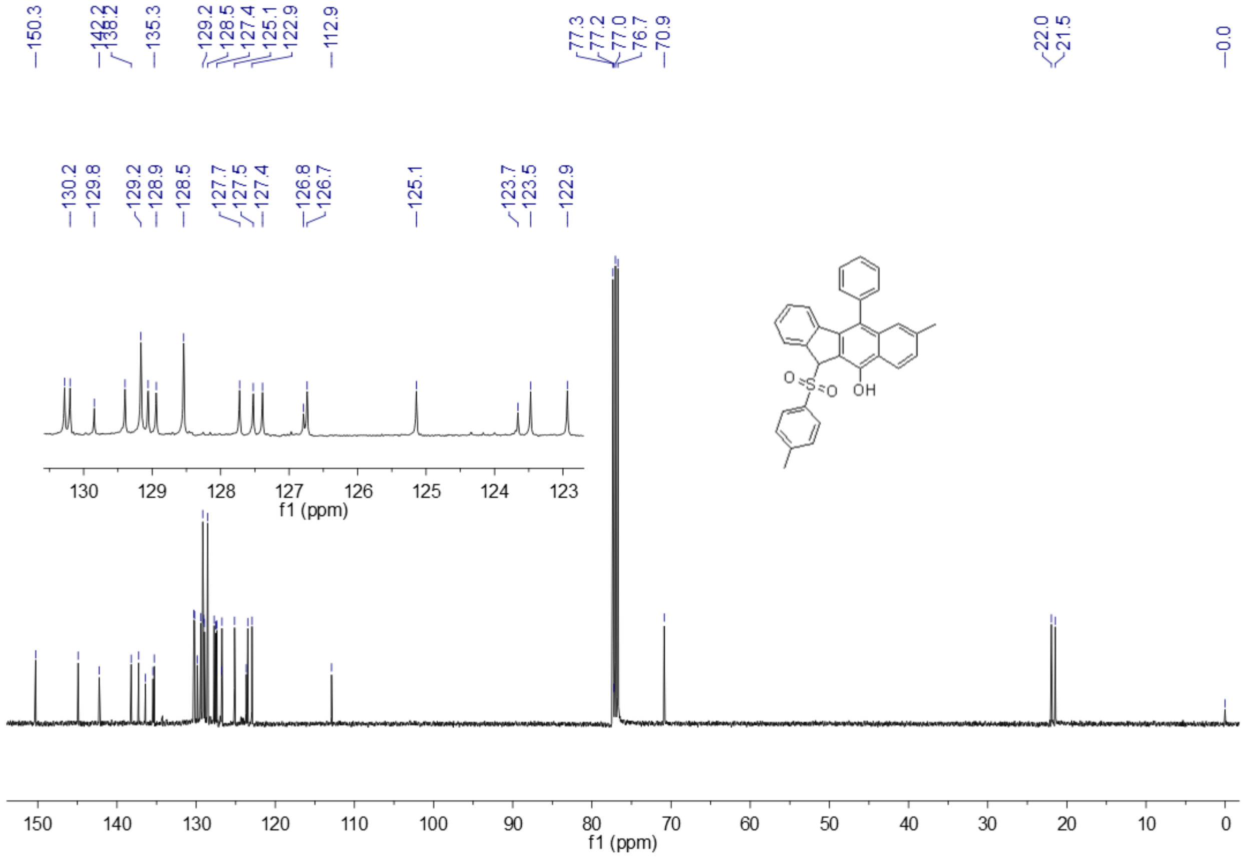


**¹³C NMR Spectrum of Compound 3a**

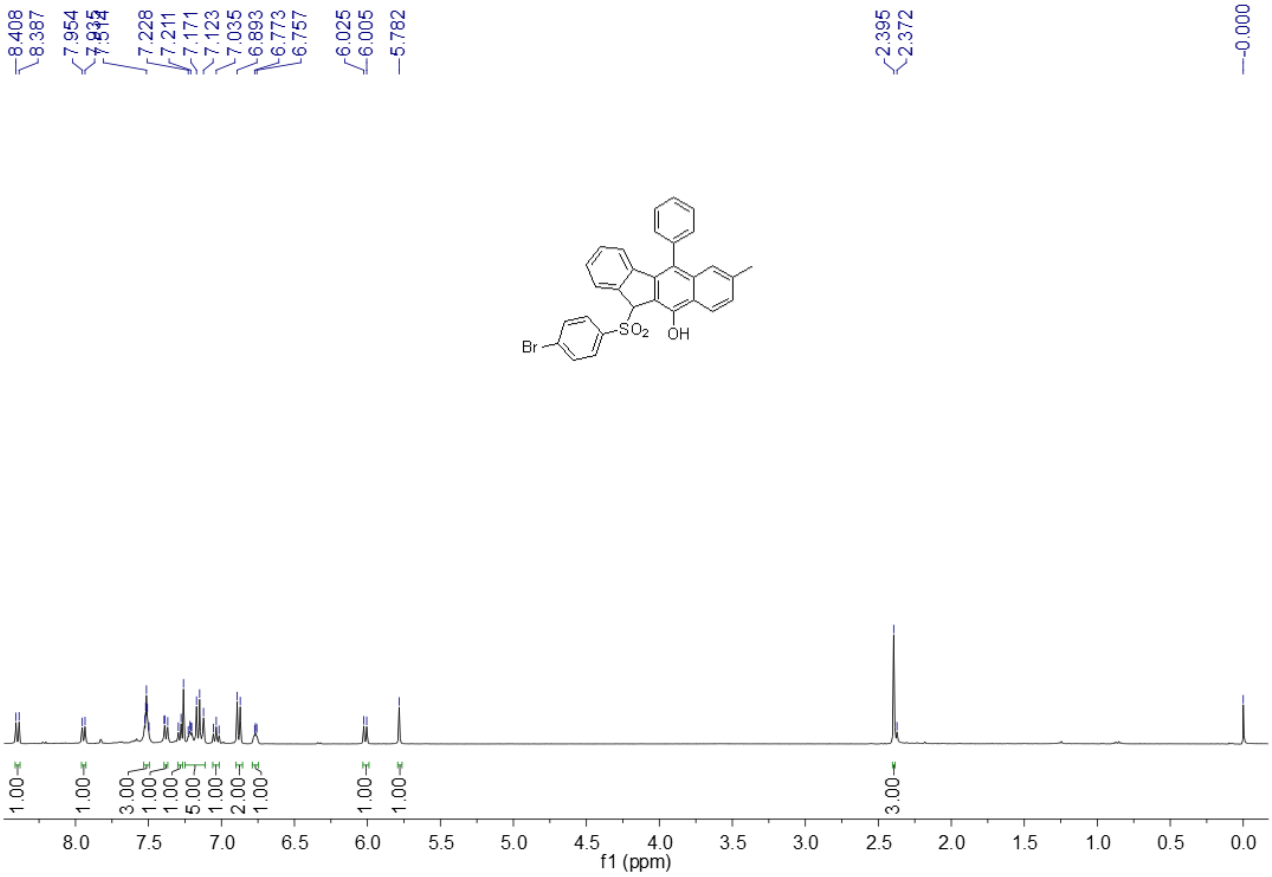


**$^{1}$H NMR Spectrum of Compound 3b**

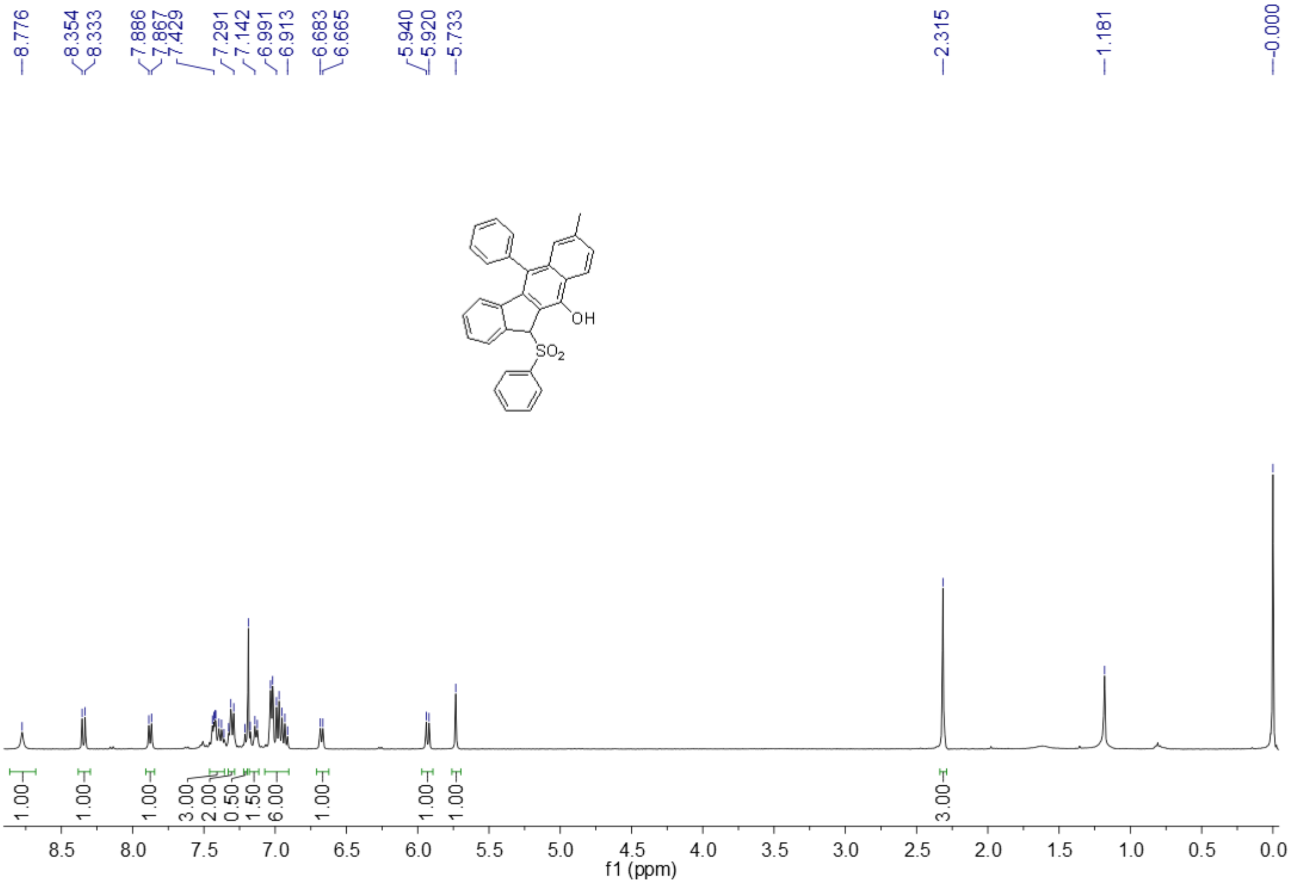


**$^{1}$H NMR Spectrum of Compound 3c**

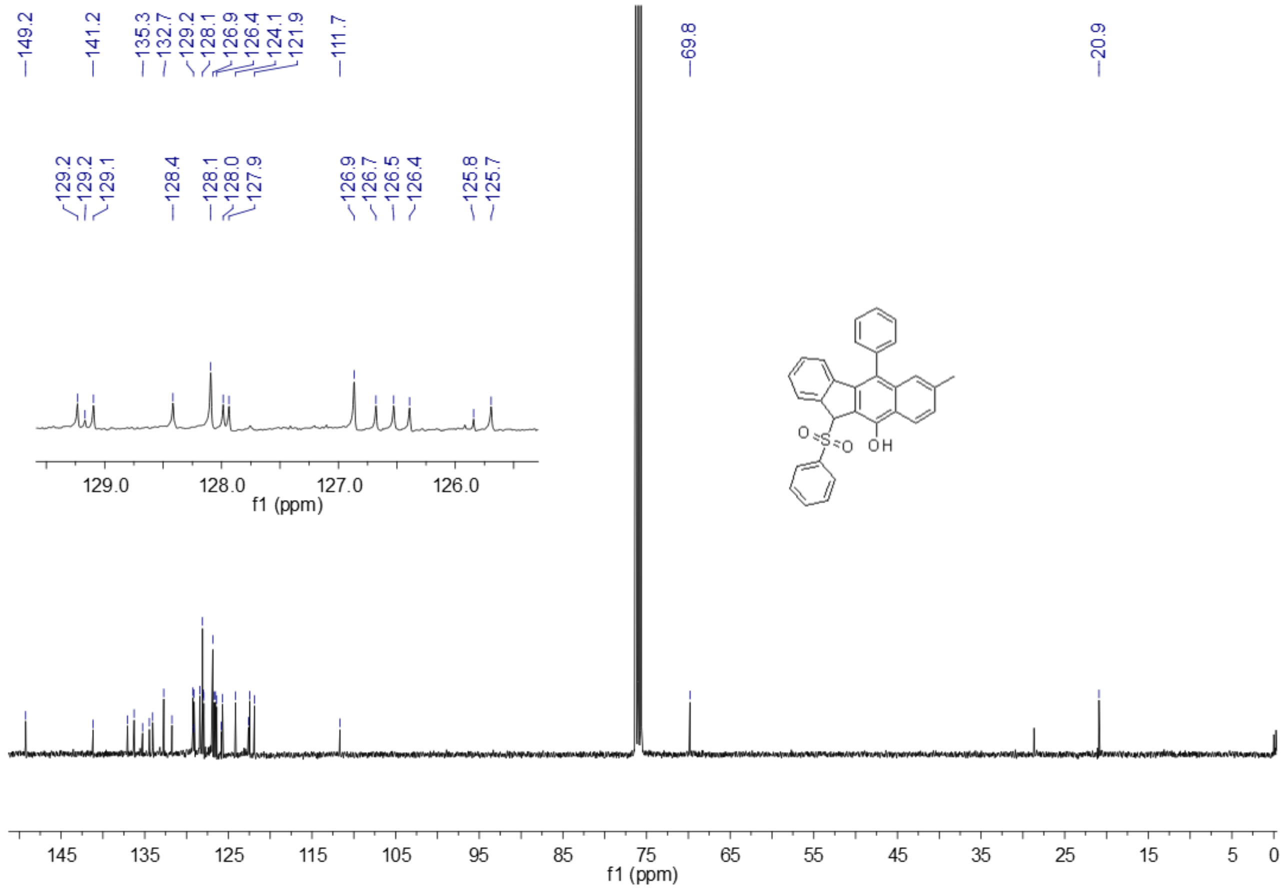


**$^{13}$C NMR Spectrum of Compound 3c**

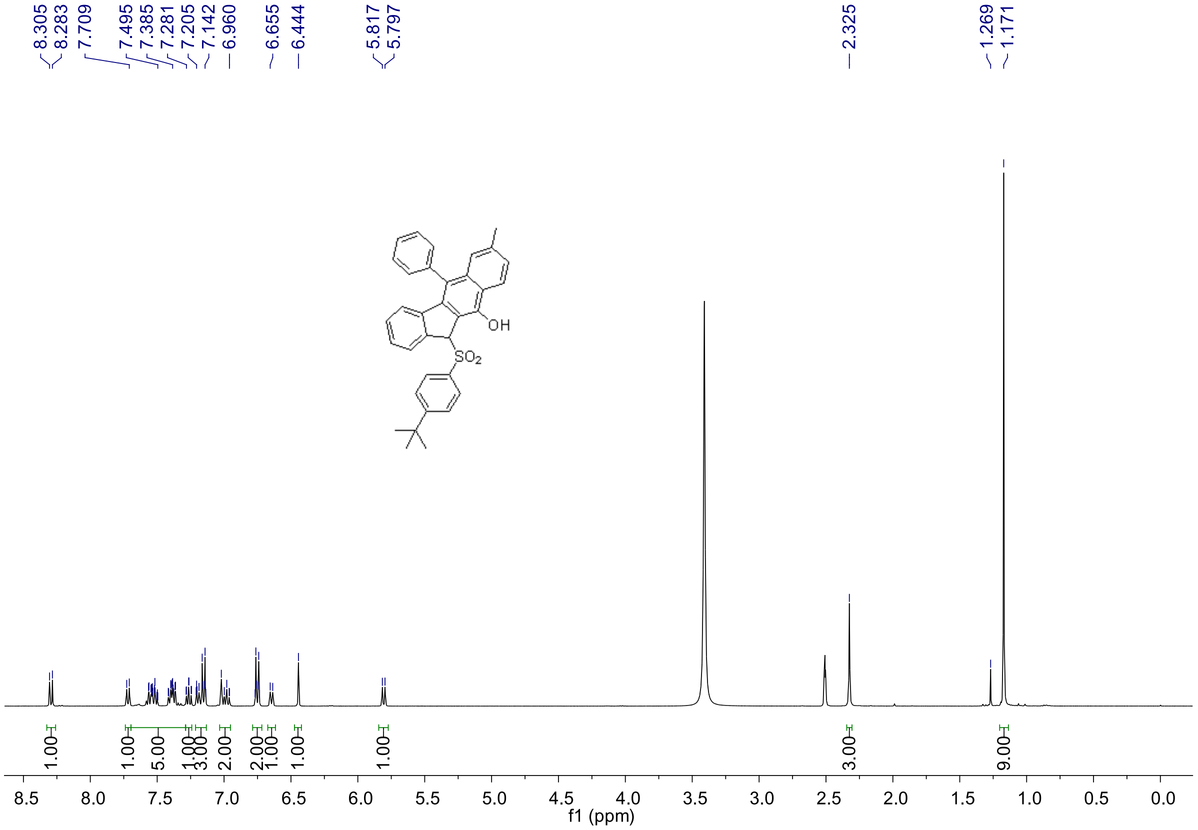


**$^{1}$H NMR Spectrum of Compound 3d**

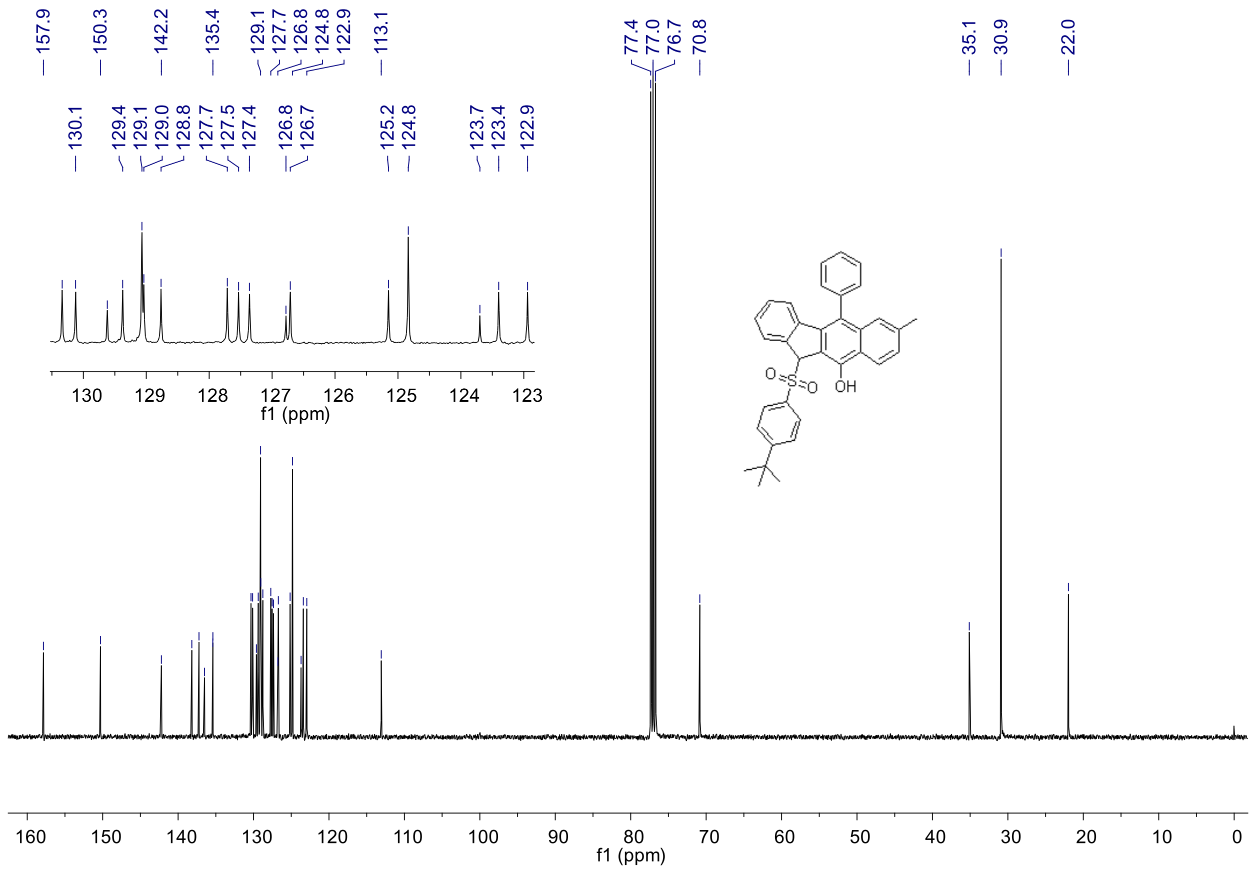


**$^{13}$C NMR Spectrum of Compound 3d**

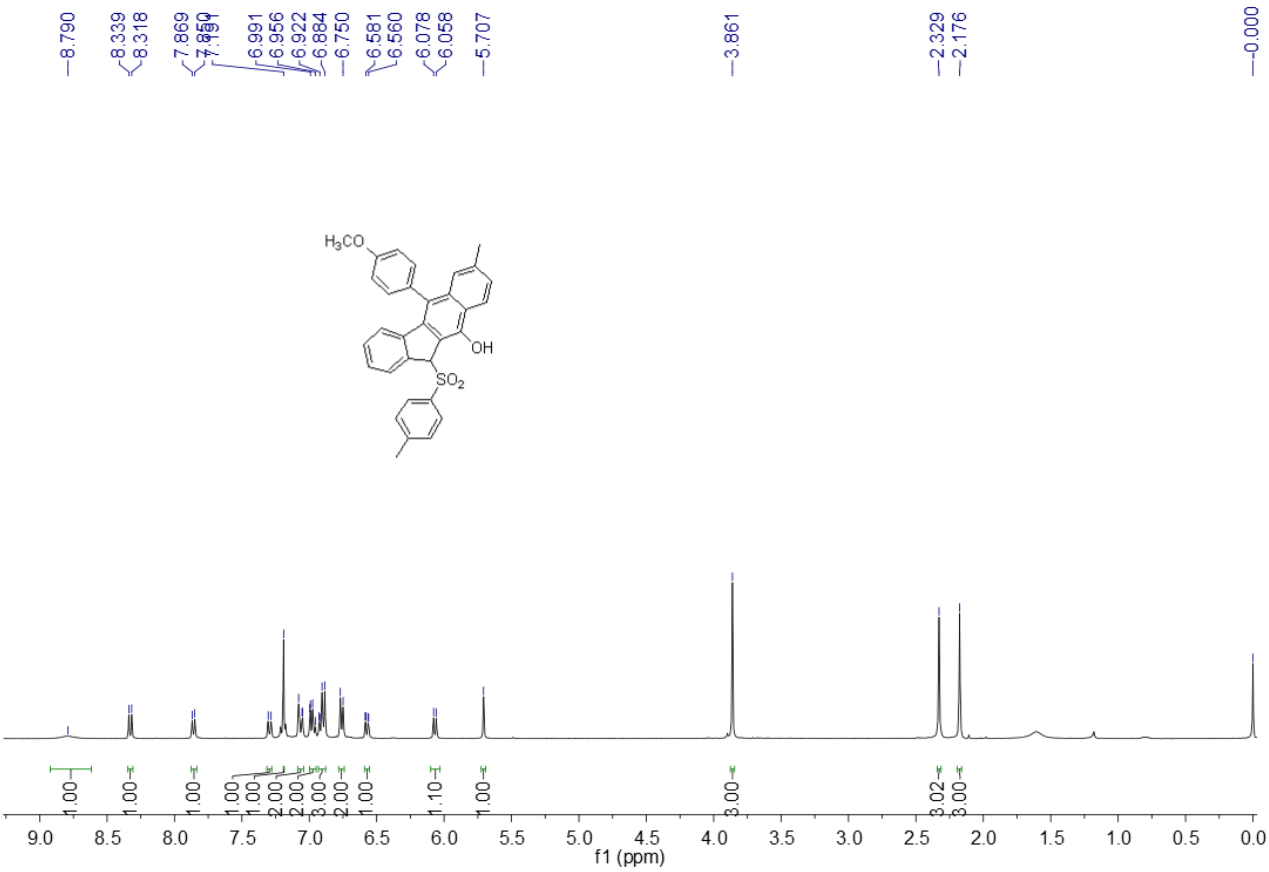


**$^{1}$H NMR Spectrum of Compound 3e**

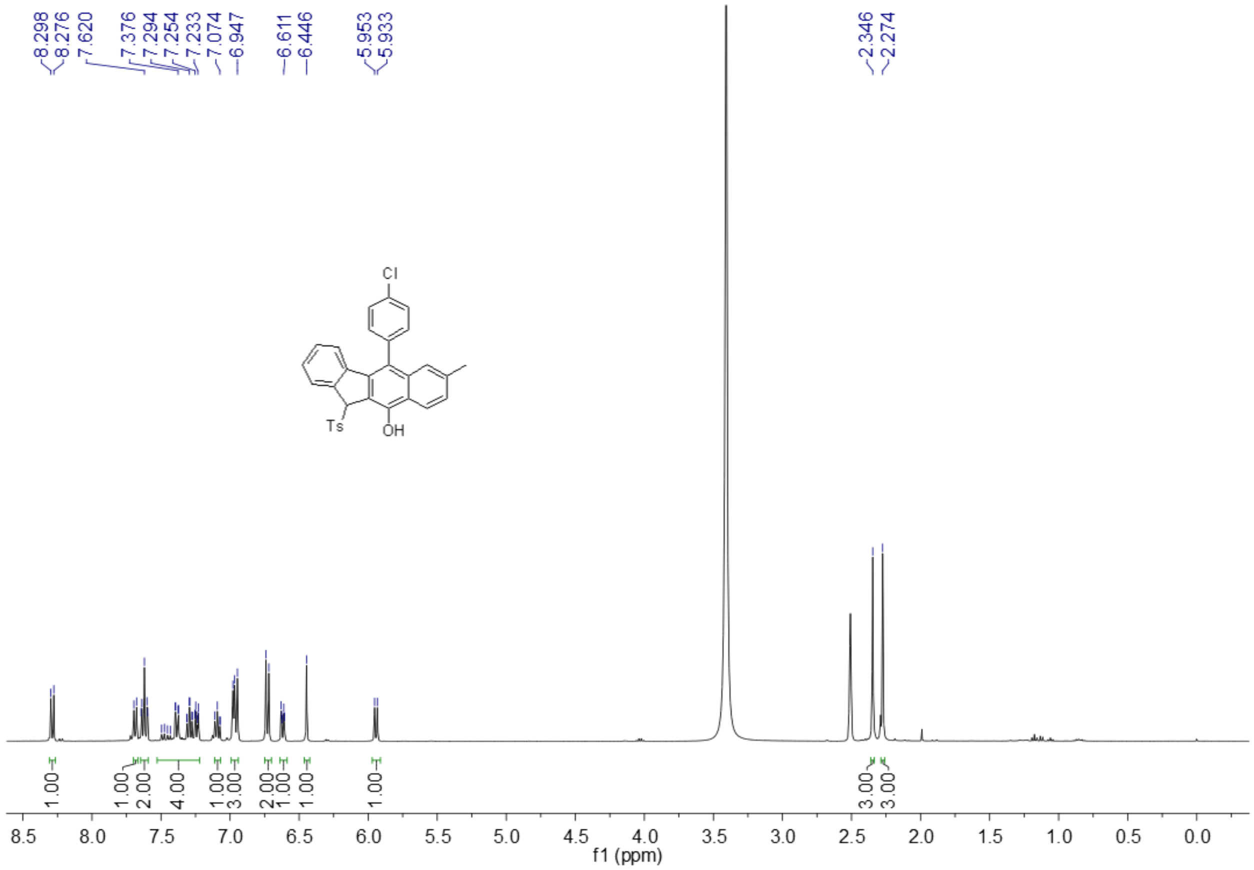


**$^{1}$H NMR Spectrum of Compound 3f**

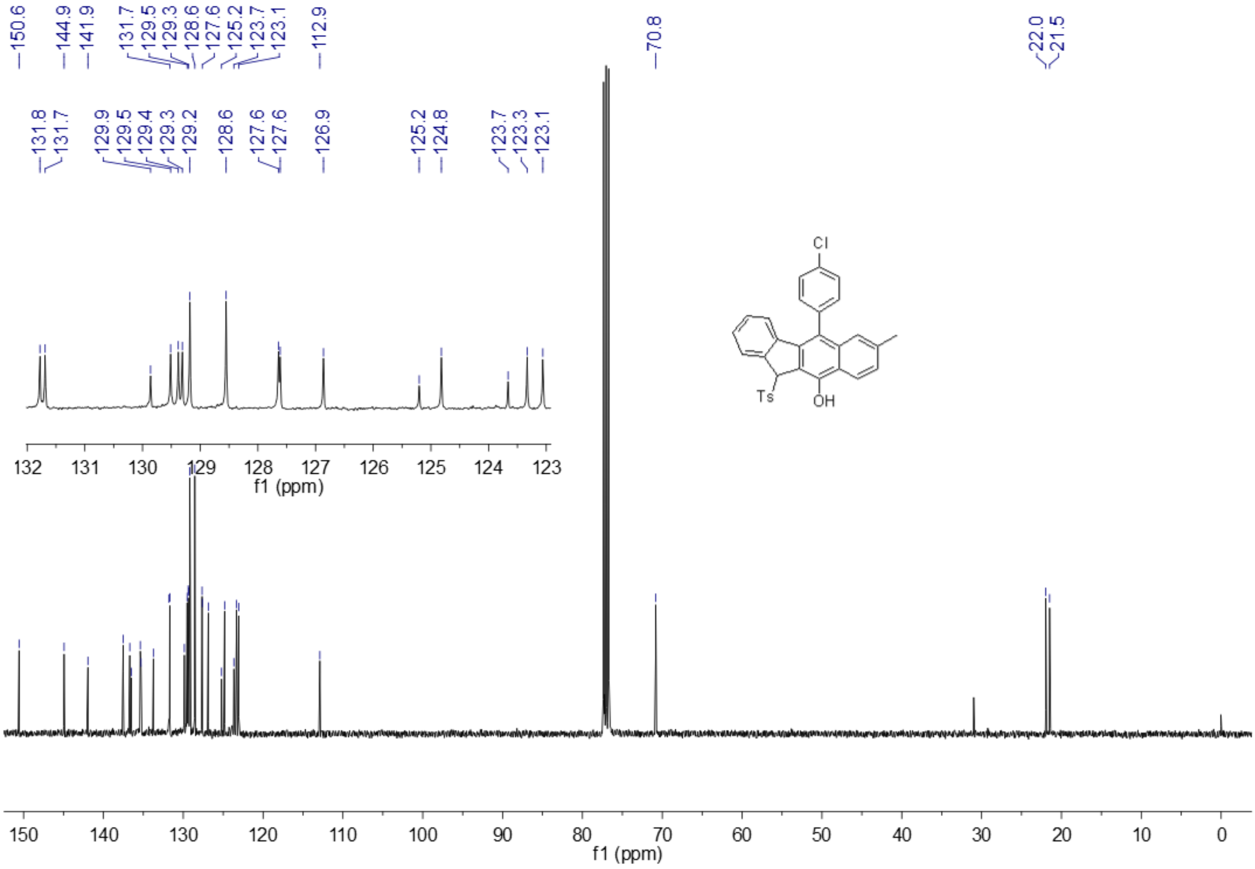


**$^{13}$C NMR Spectrum of Compound 3f**

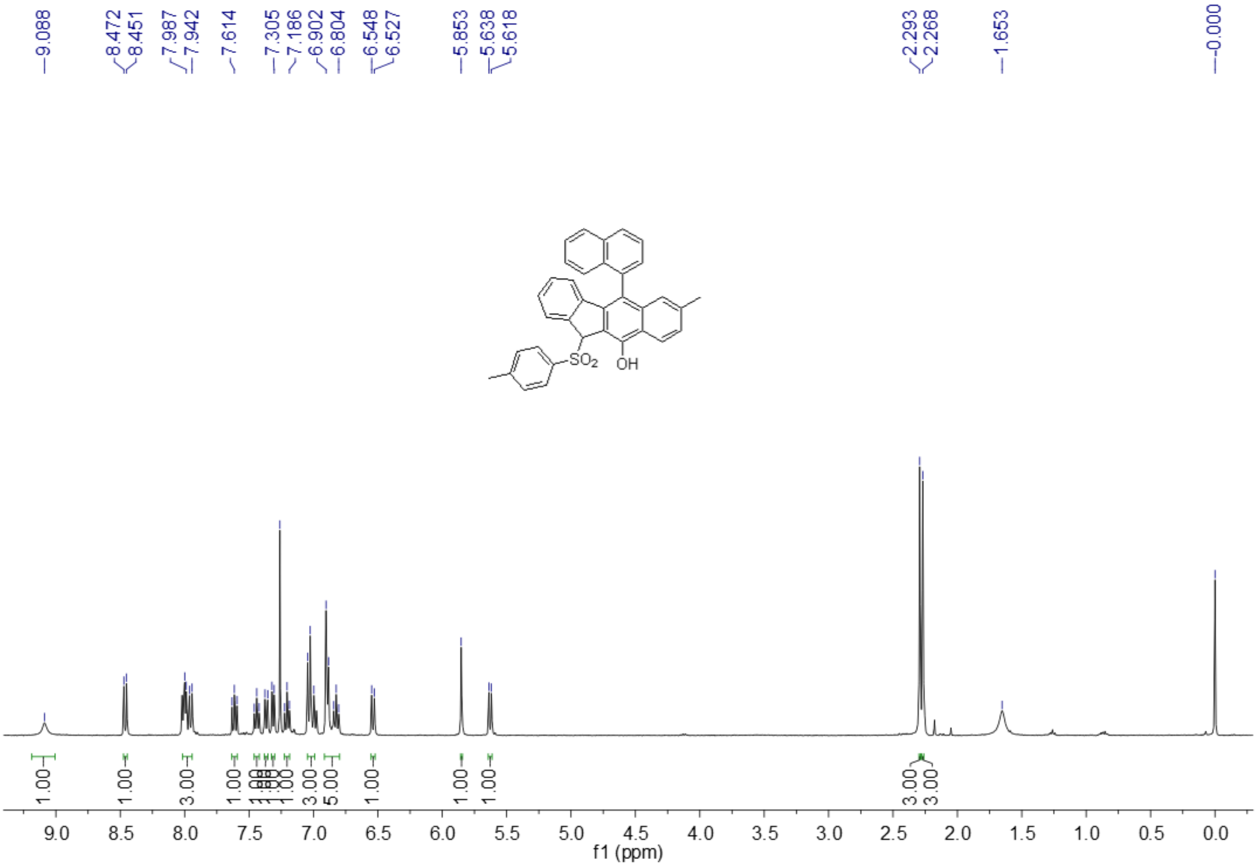

**$^{1}$H NMR Spectrum of Compound 3g**

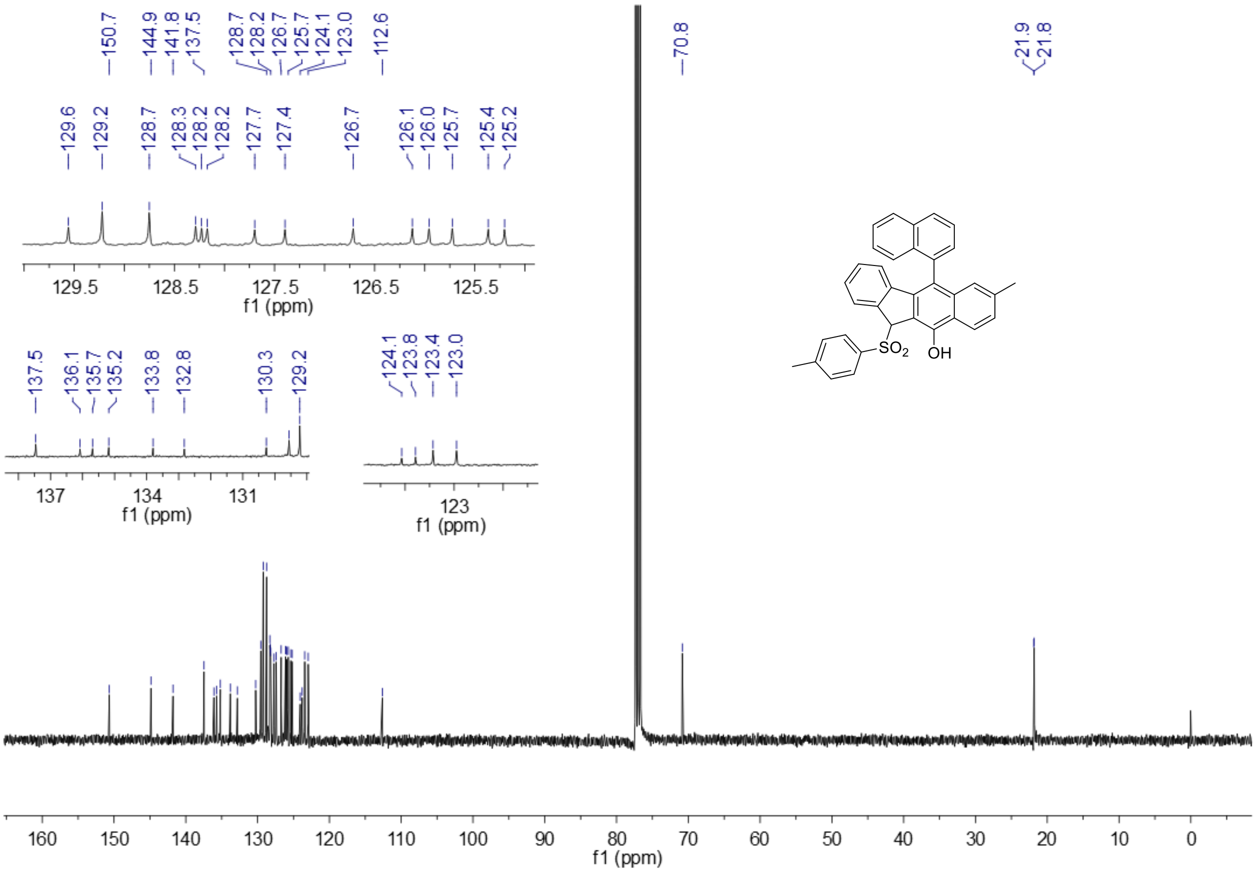

**$^{13}$C NMR Spectrum of Compound 3g**

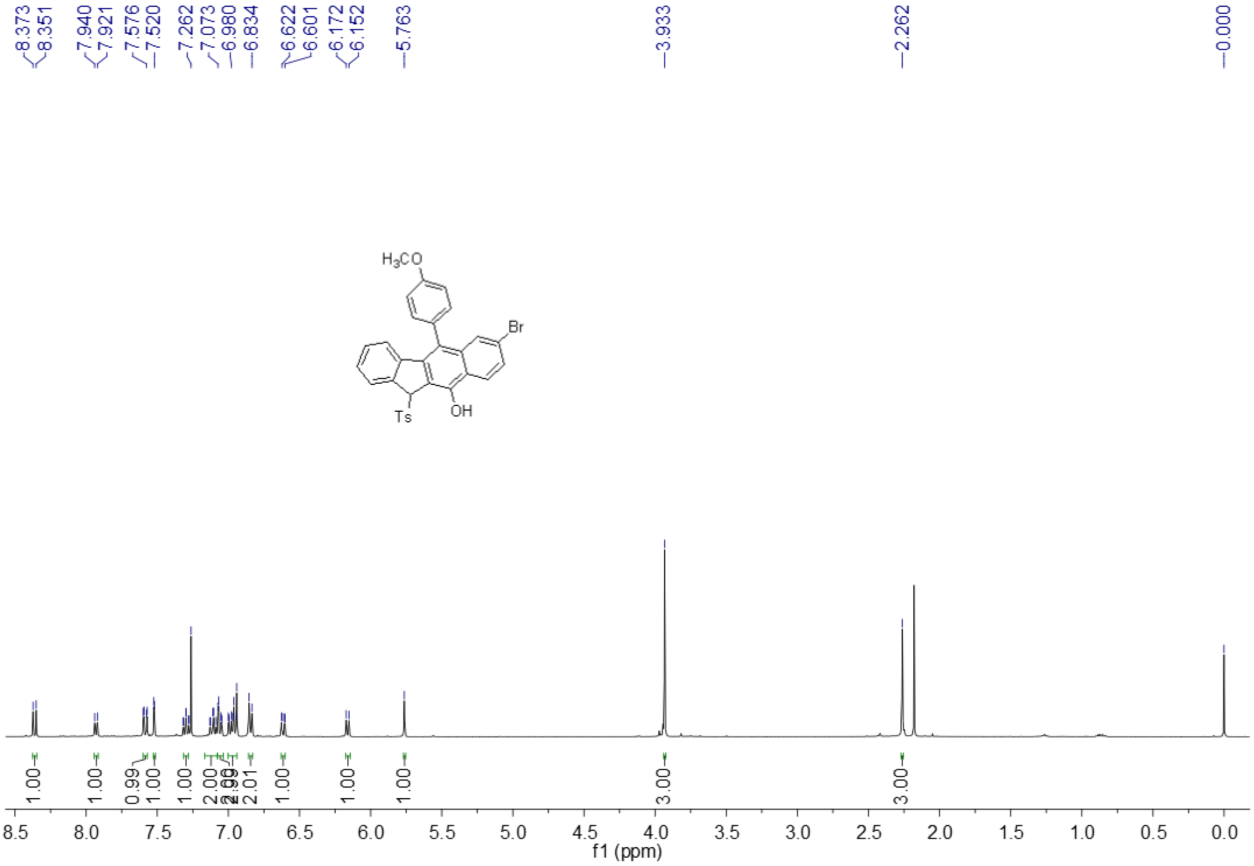


**$^{1}$H NMR Spectrum of Compound 3h**

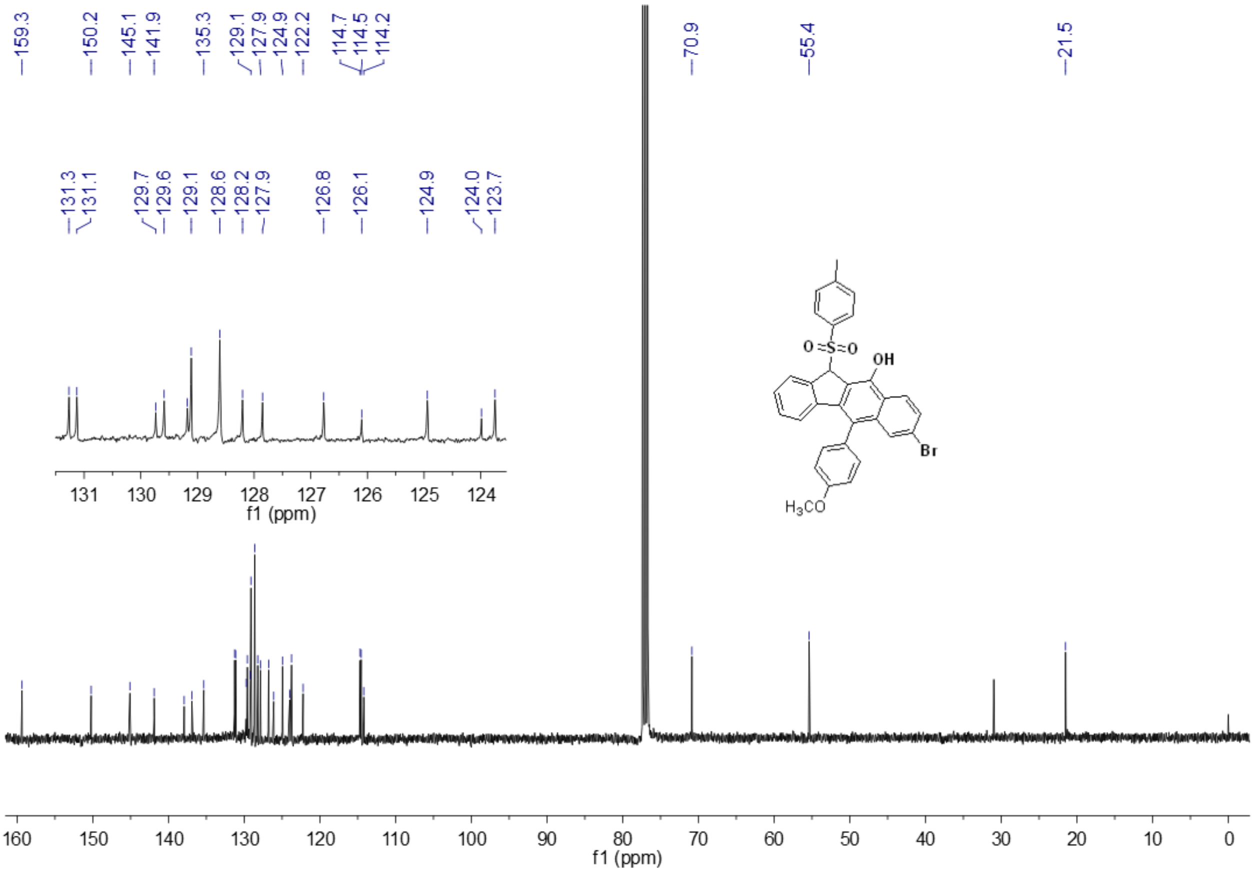


**$^{13}$C NMR Spectrum of Compound 3h**

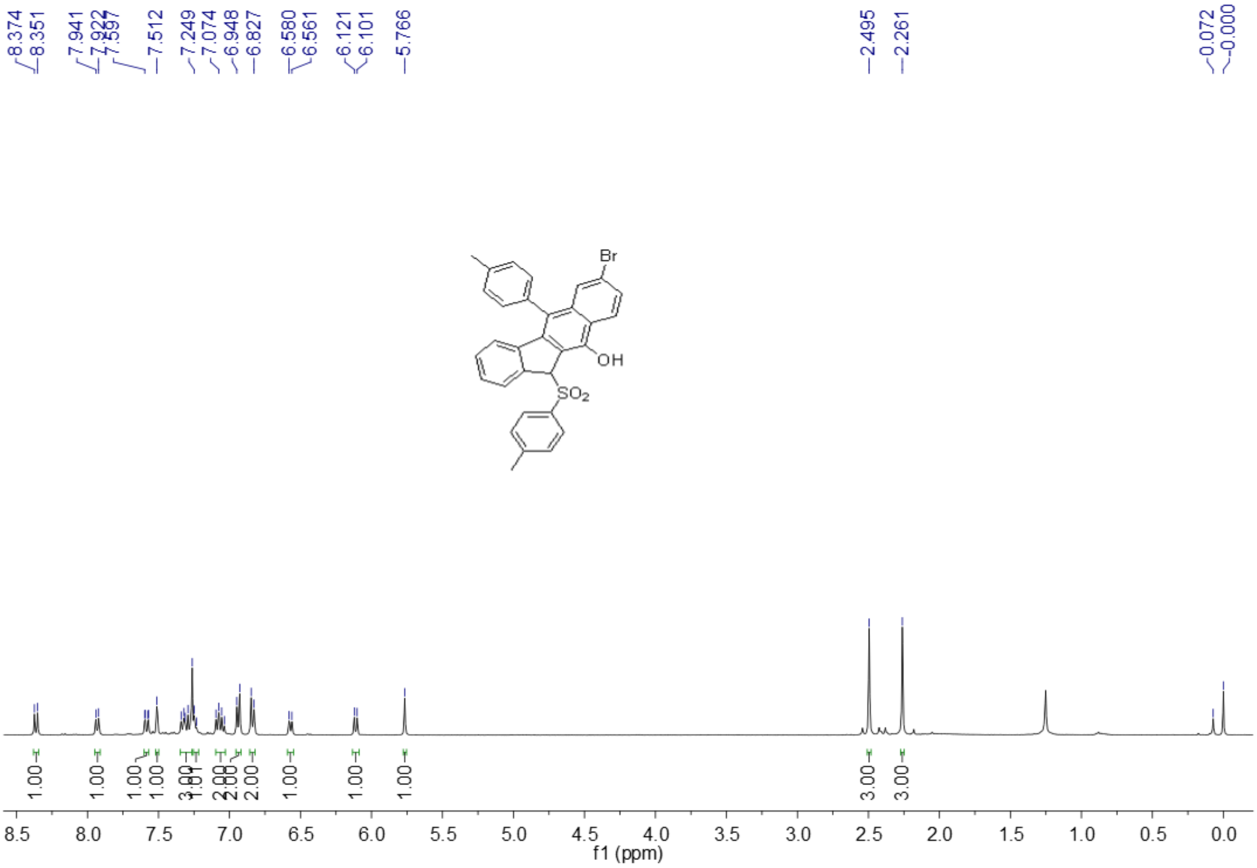


$^{1}$H NMR Spectrum of Compound 3i

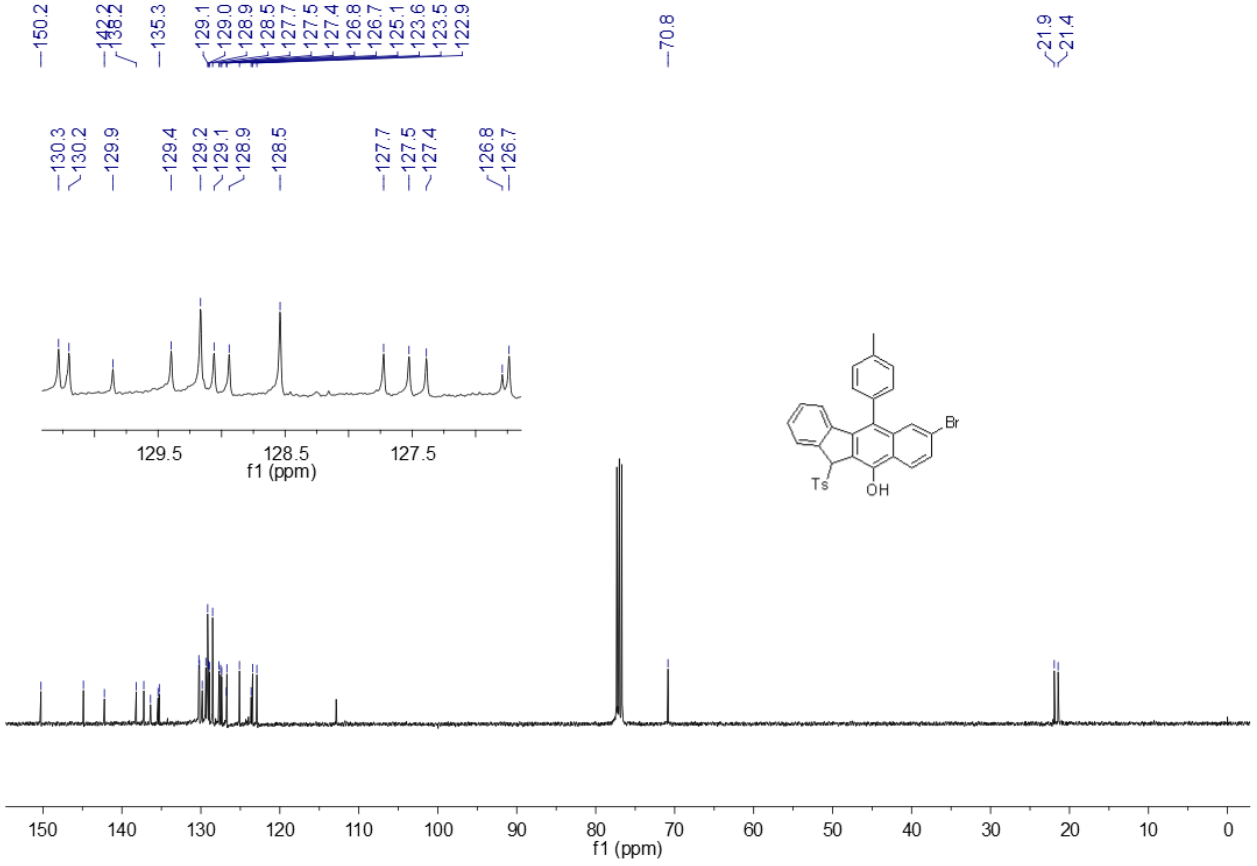


$^{13}$C NMR Spectrum of Compound 3i

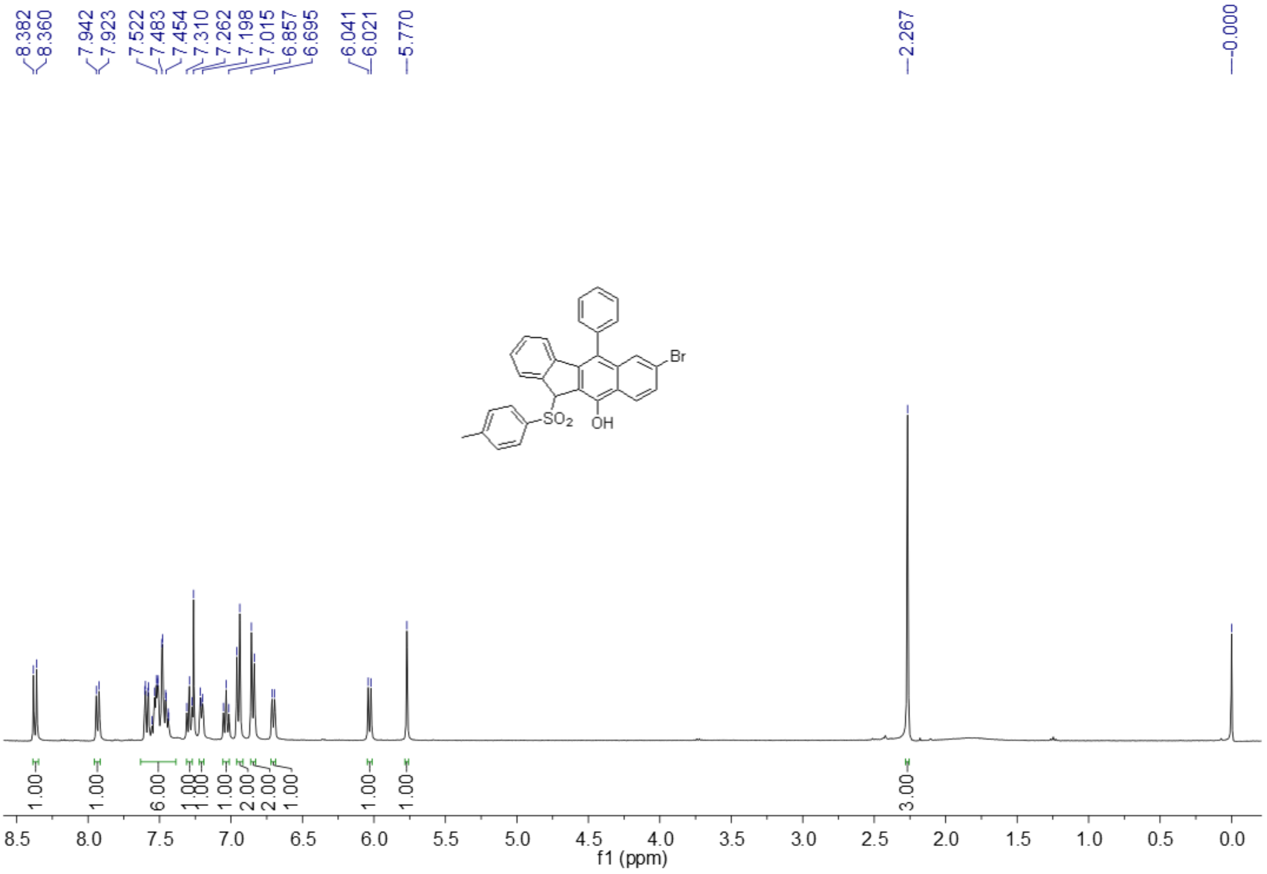


**$^{1}$H NMR Spectrum of Compound 3j**

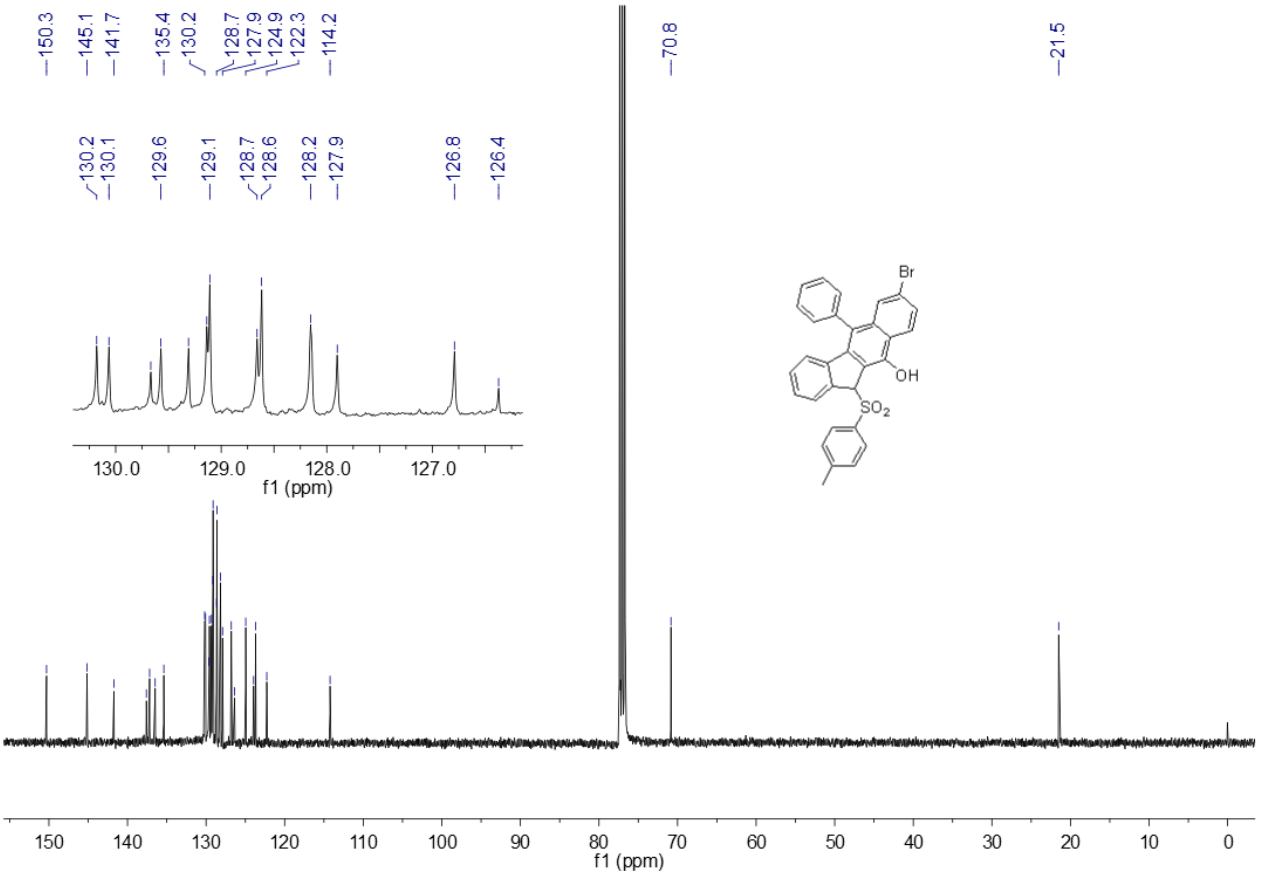


**$^{13}$C NMR Spectrum of Compound 3j**

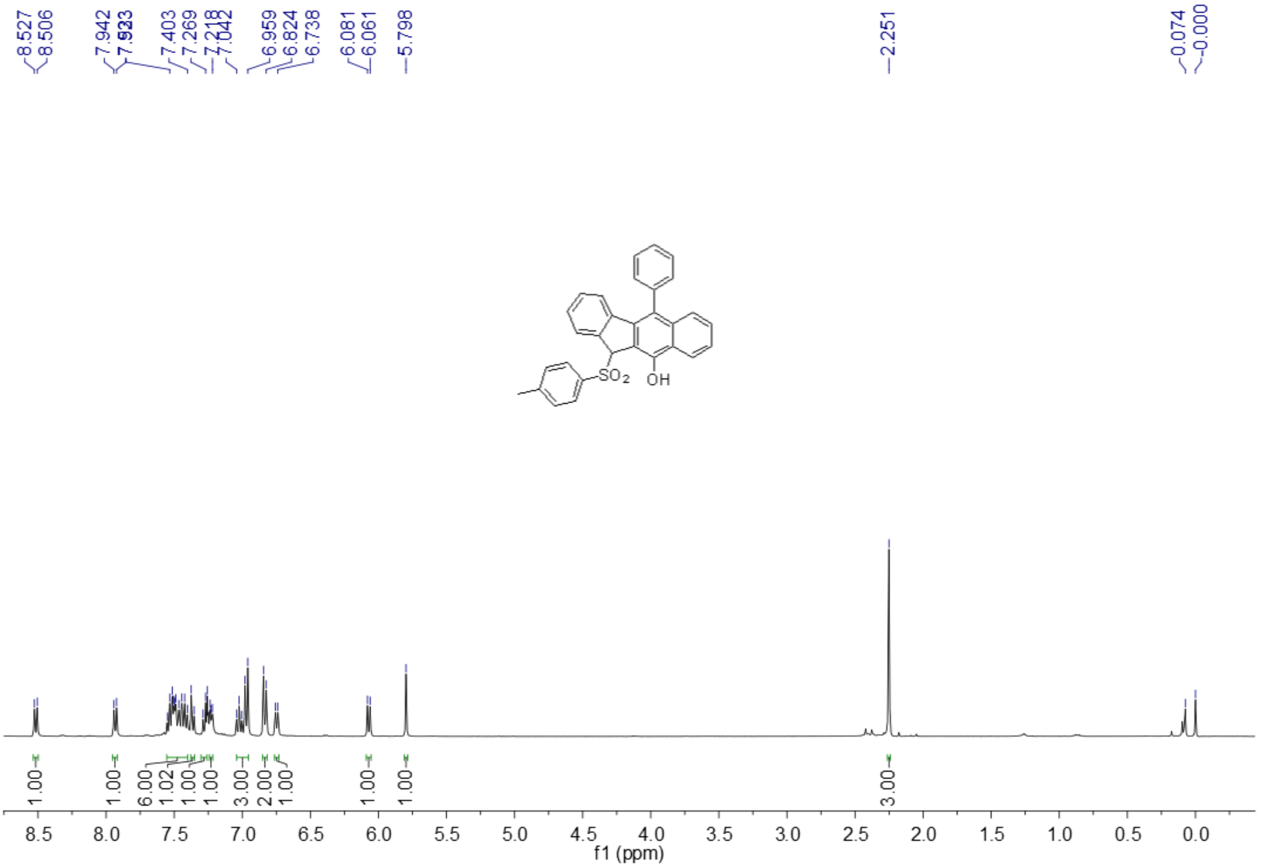


**$^{1}$H NMR Spectrum of Compound 3k**

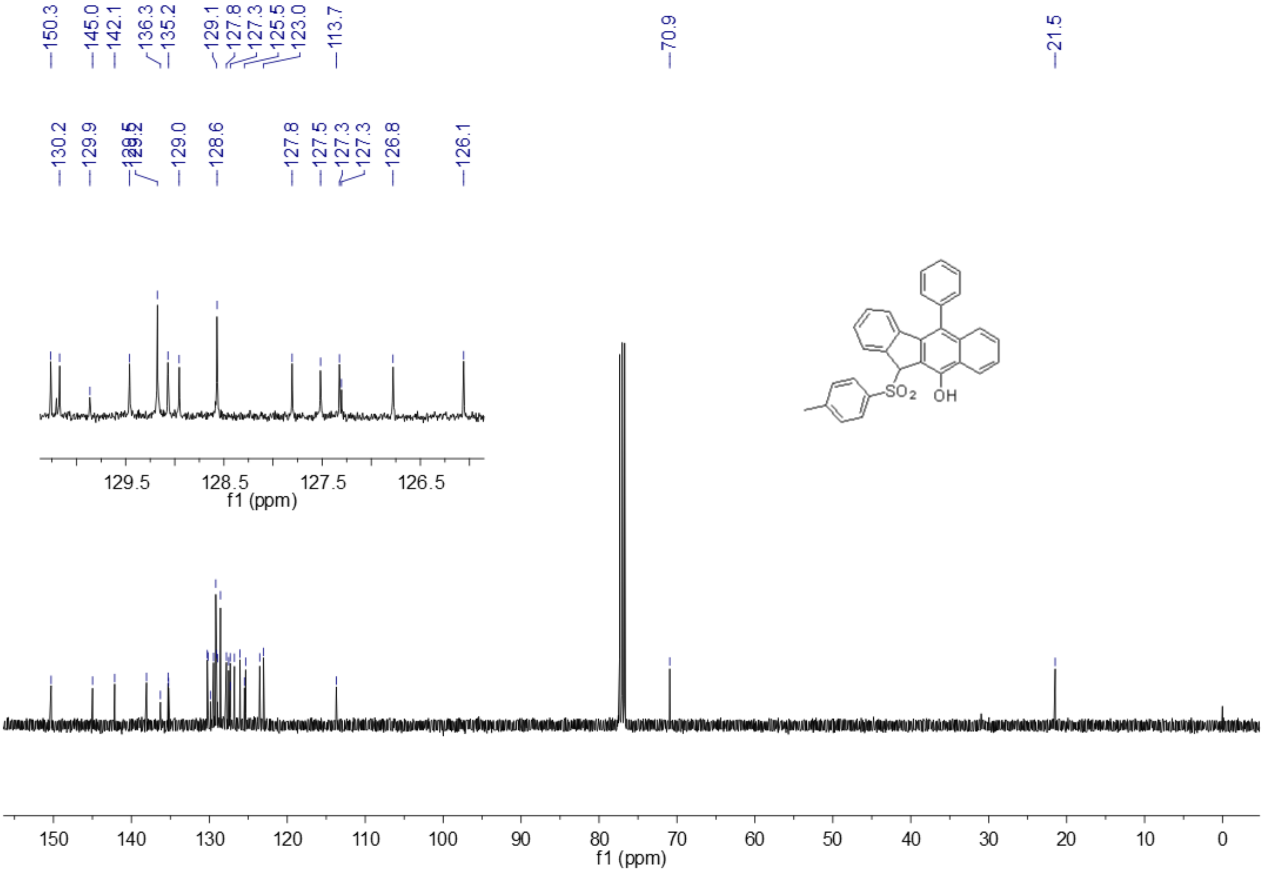


**$^{13}$C NMR Spectrum of Compound 3k**

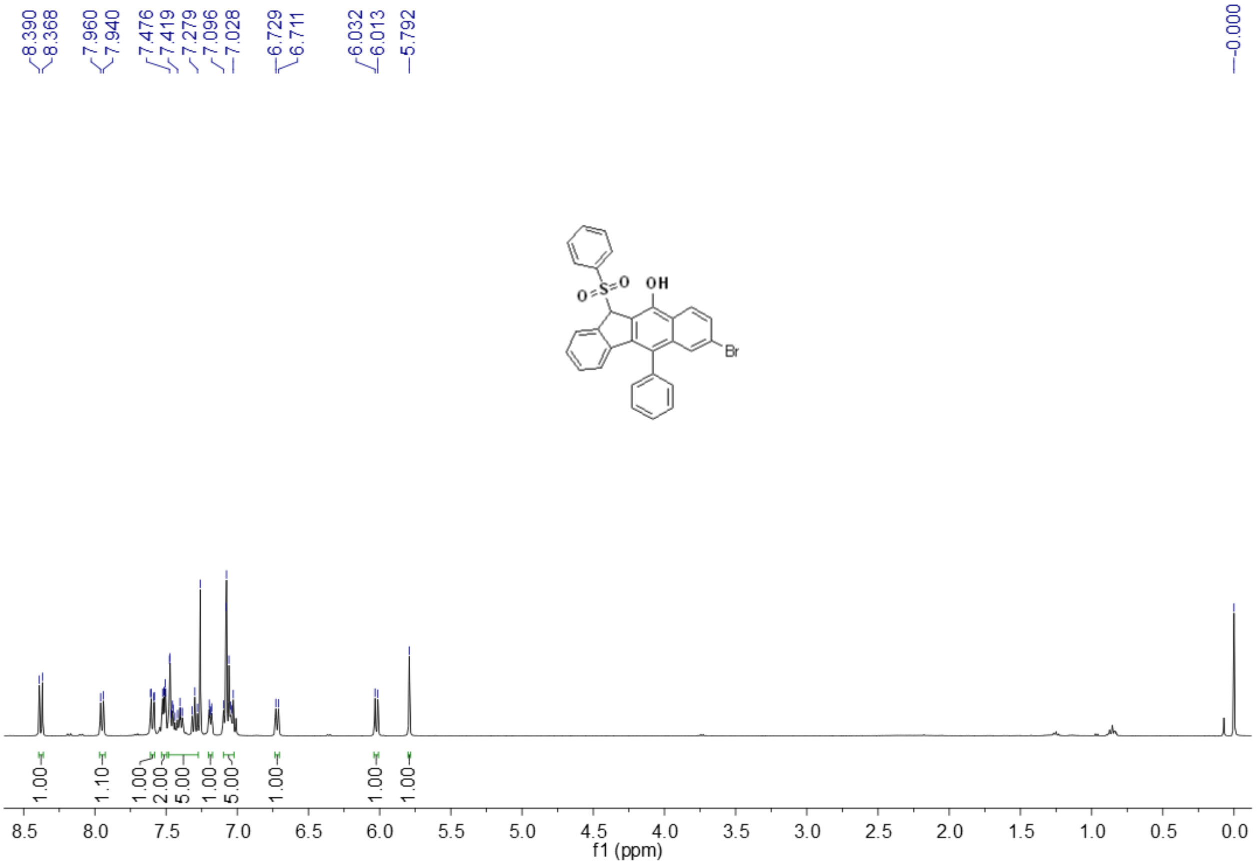


**$^{1}$H NMR Spectrum of Compound 3l**

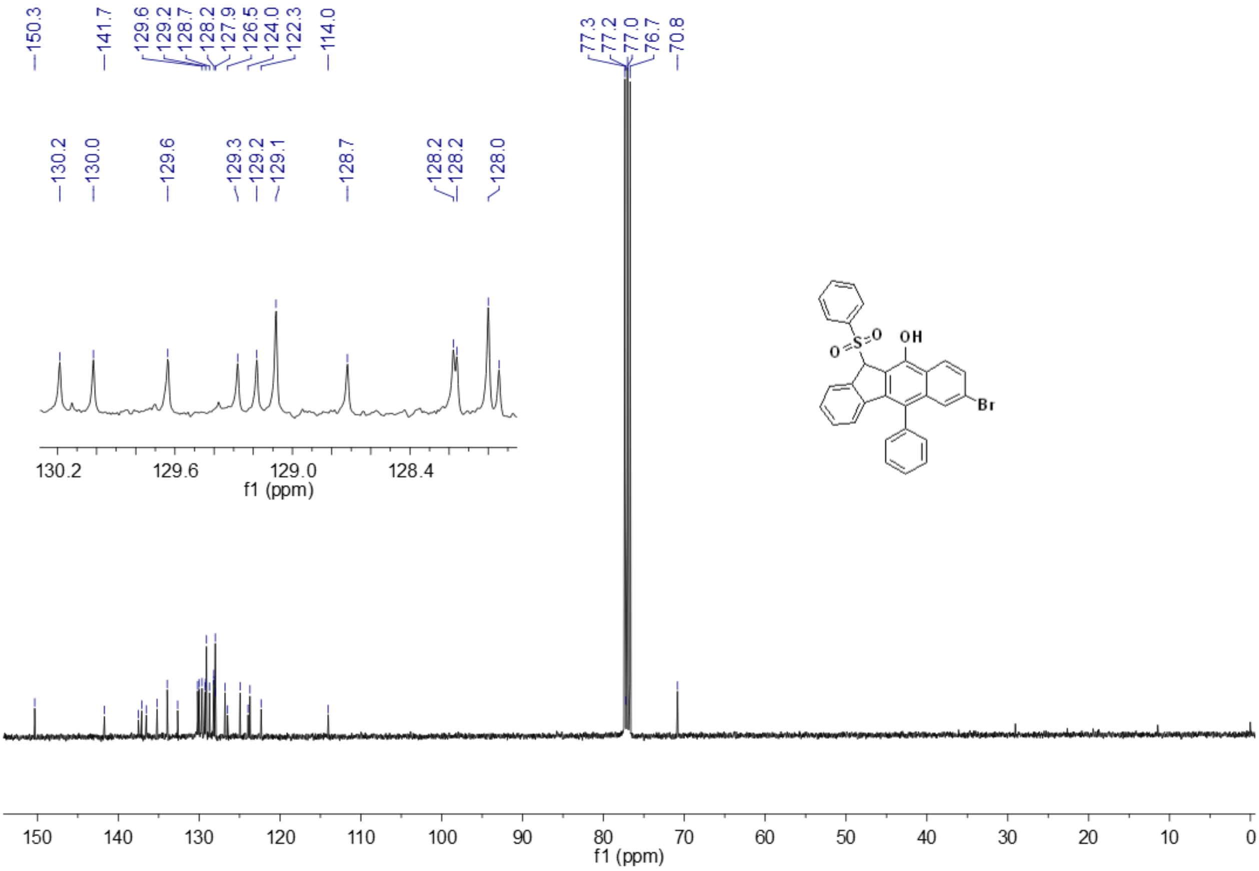


**$^{13}$C NMR Spectrum of Compound 3l**

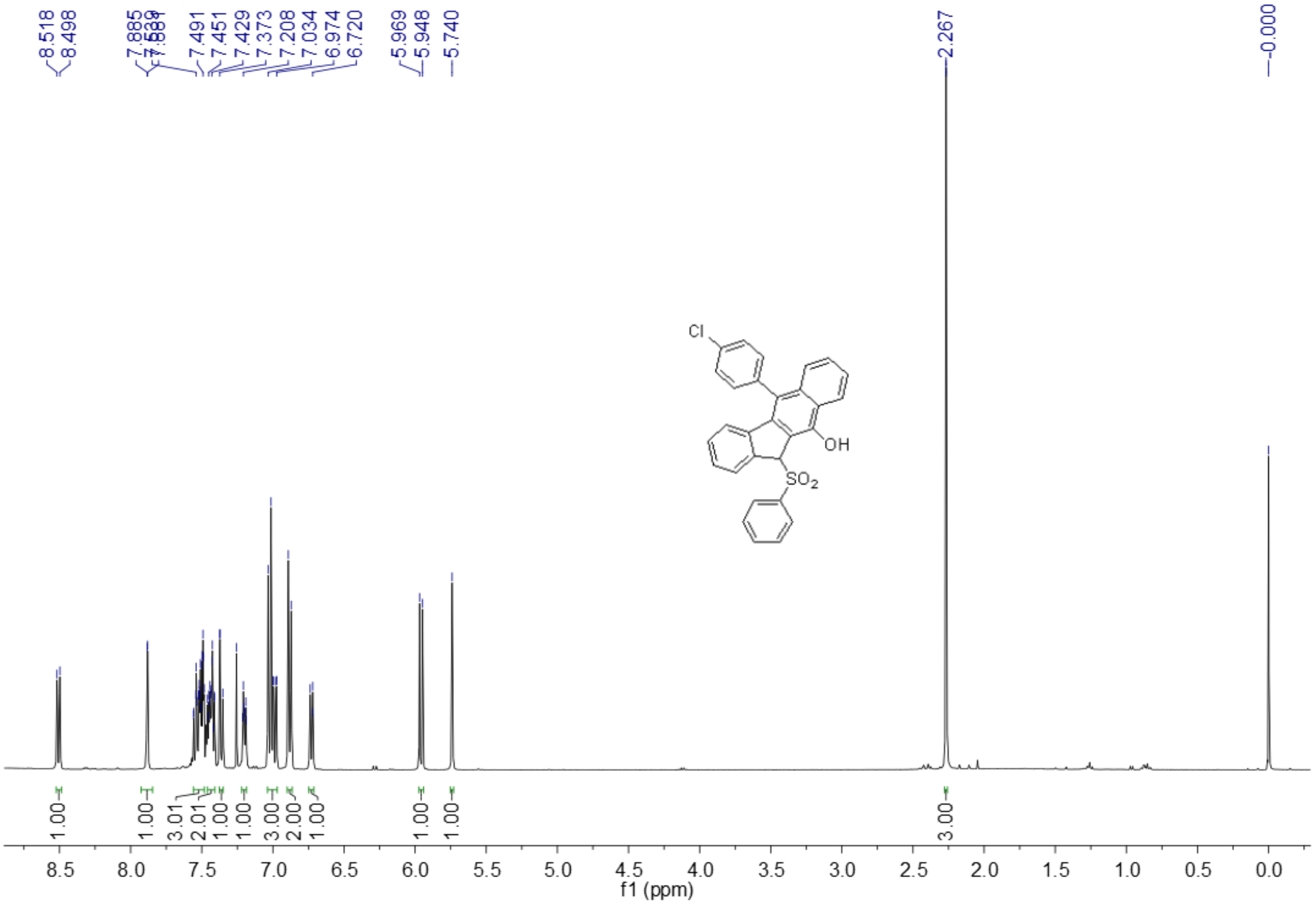

# $^{1}$H NMR Spectrum of Compound 3m

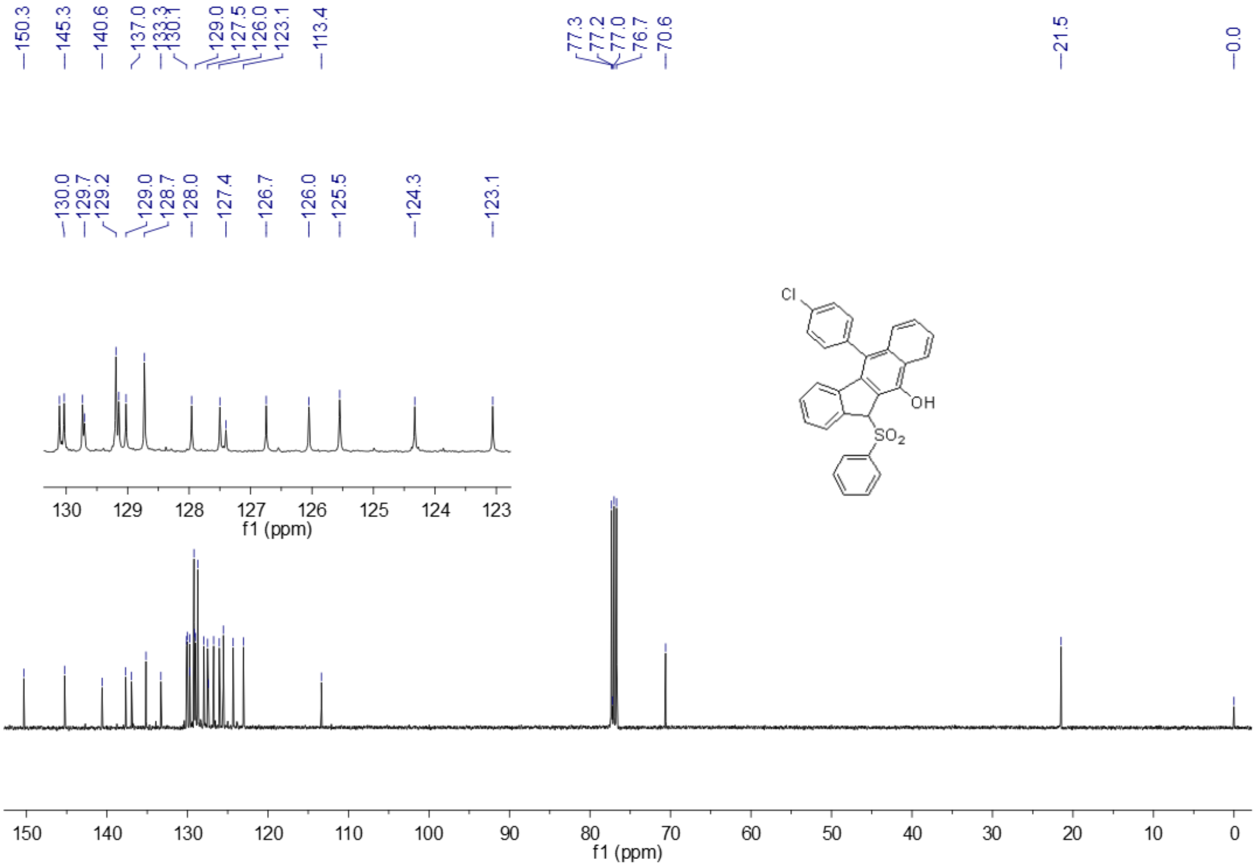

# $^{13}$C NMR Spectrum of Compound 3m

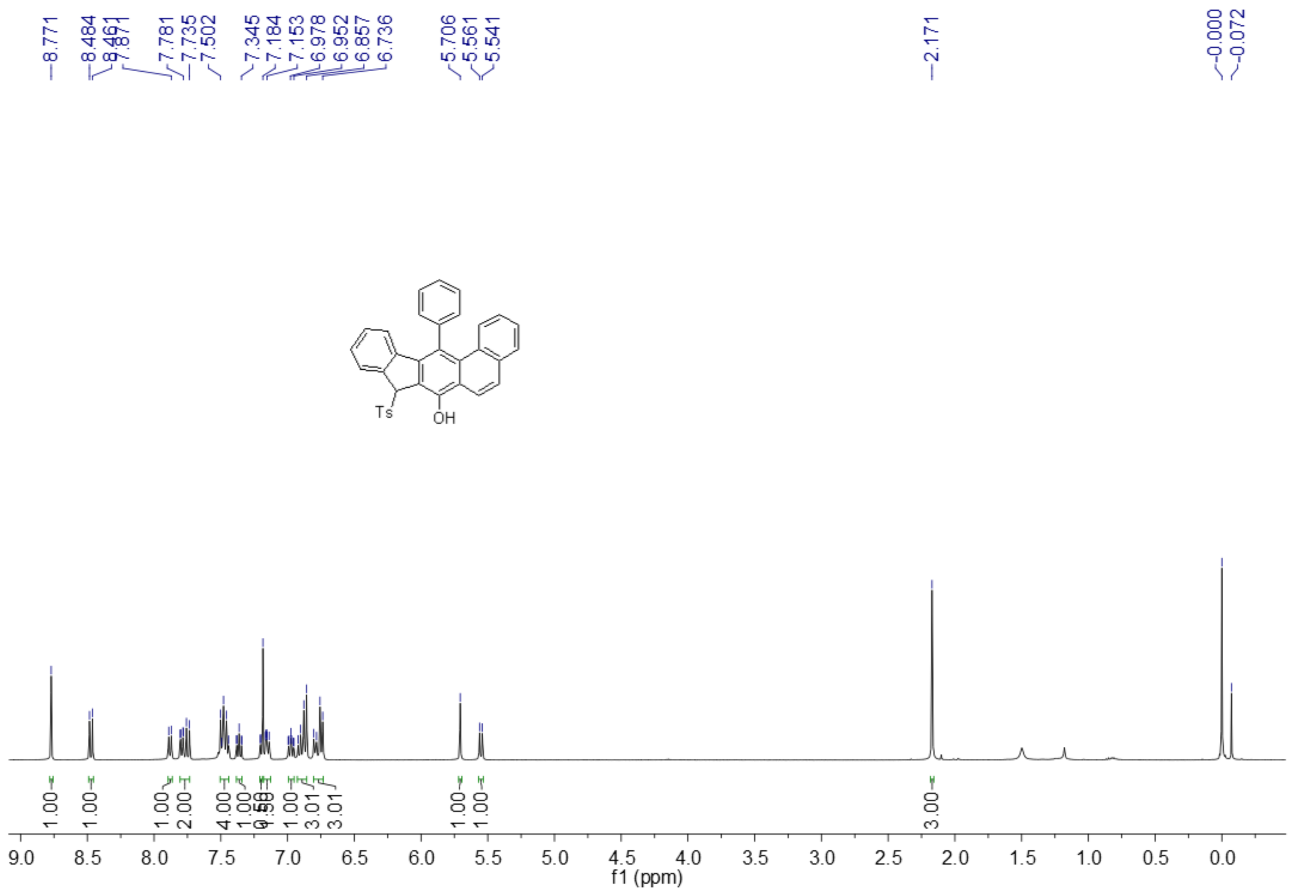


**$^{1}$H NMR Spectrum of Compound 3n**

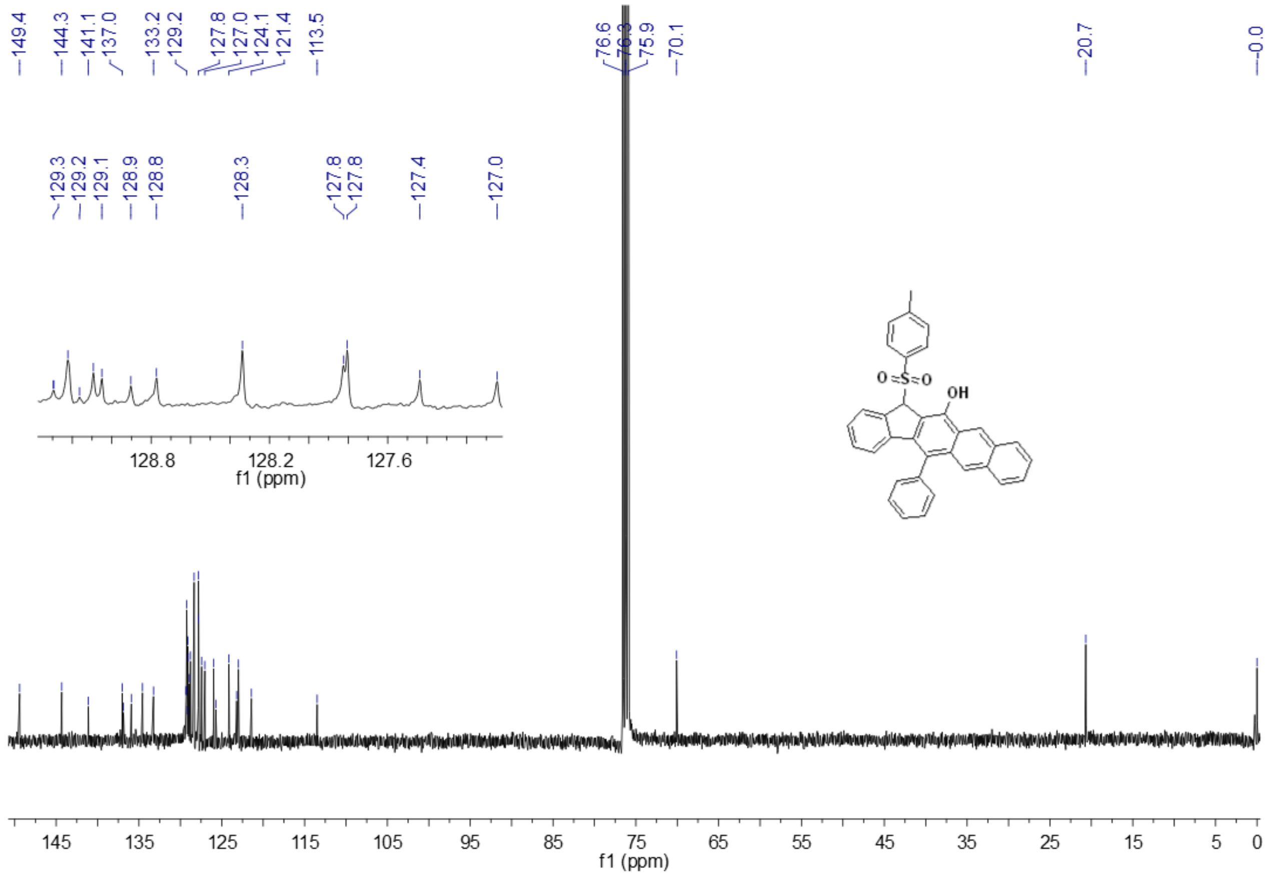


**$^{13}$C NMR Spectrum of Compound 3n**

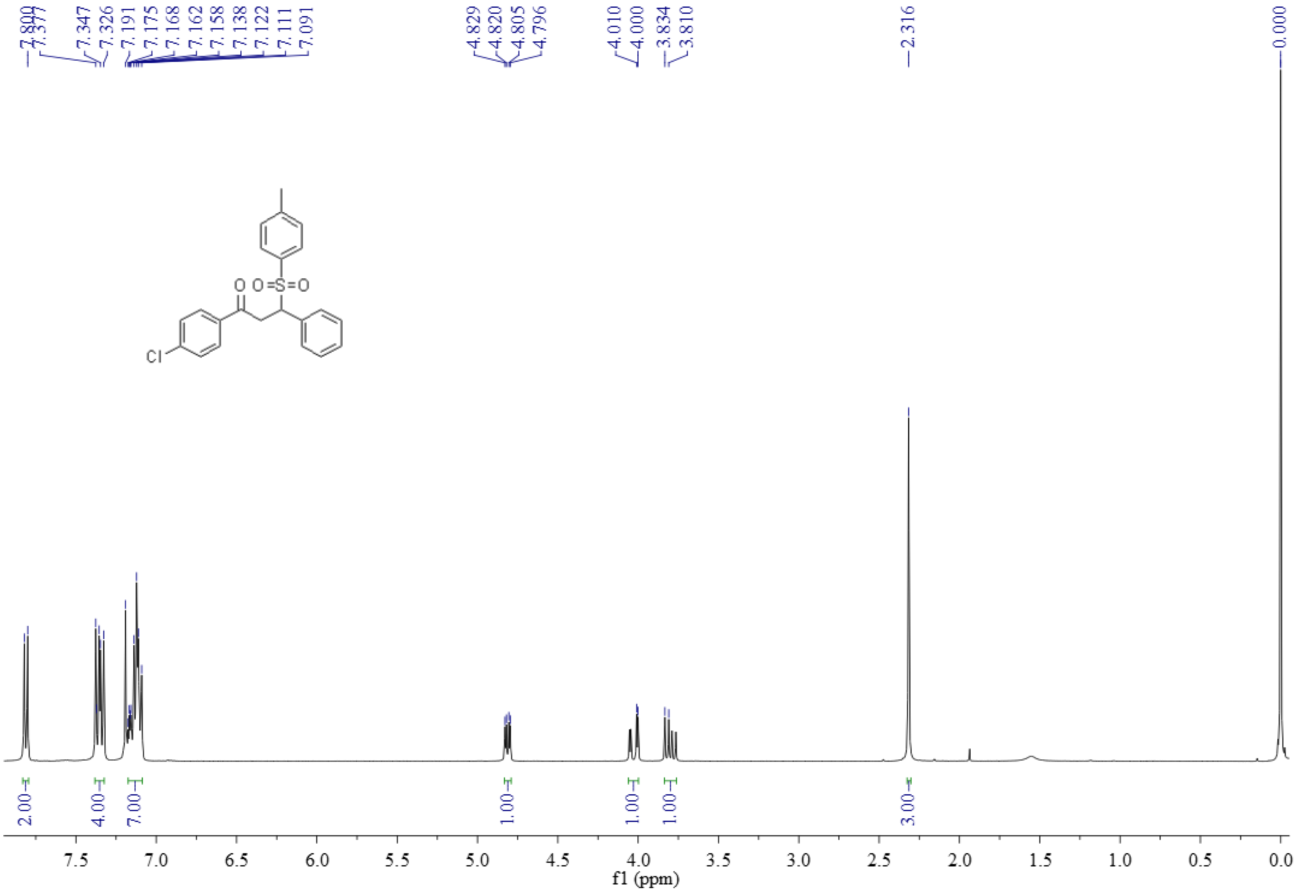


**$^{1}$H NMR Spectrum of Compound 5a**

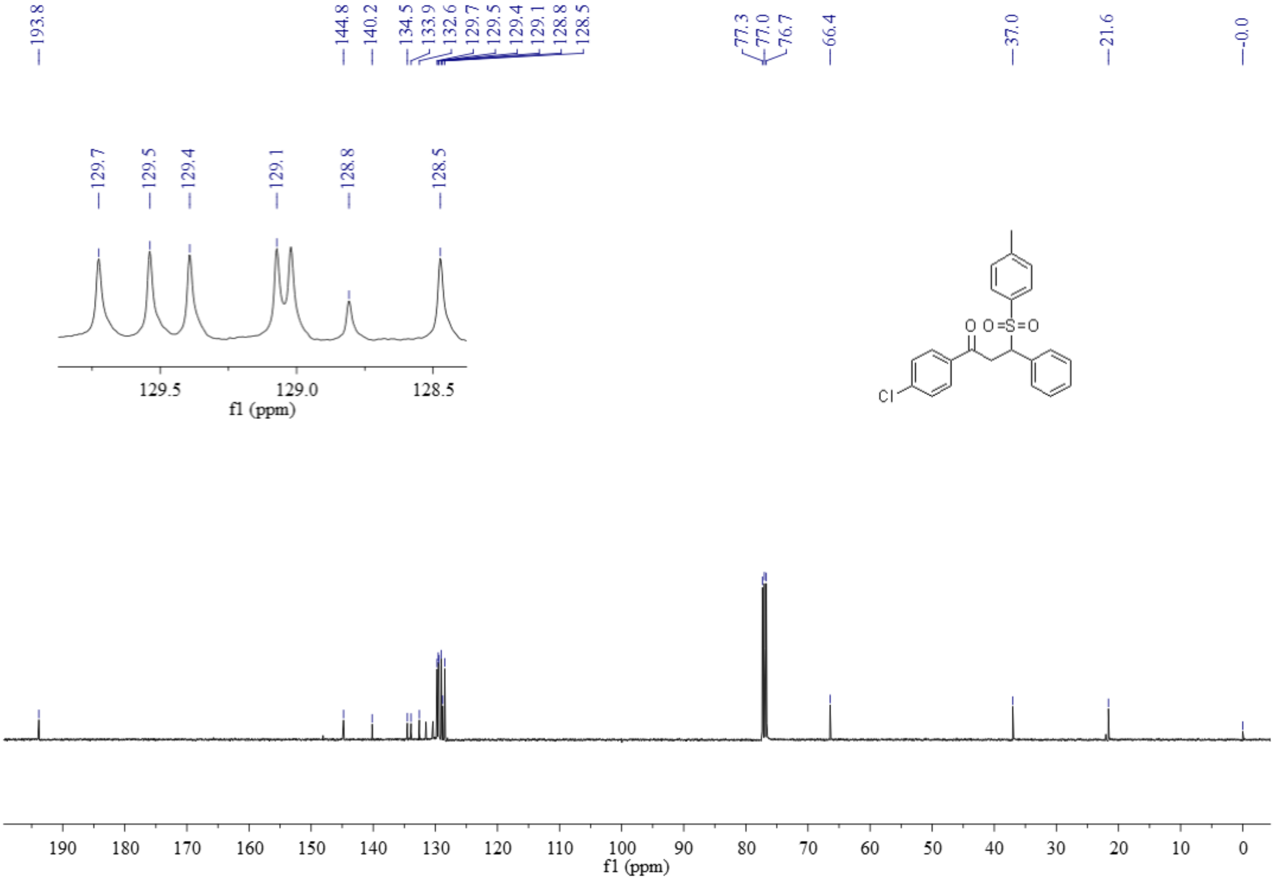


**$^{13}$C NMR Spectrum of Compound 5a**

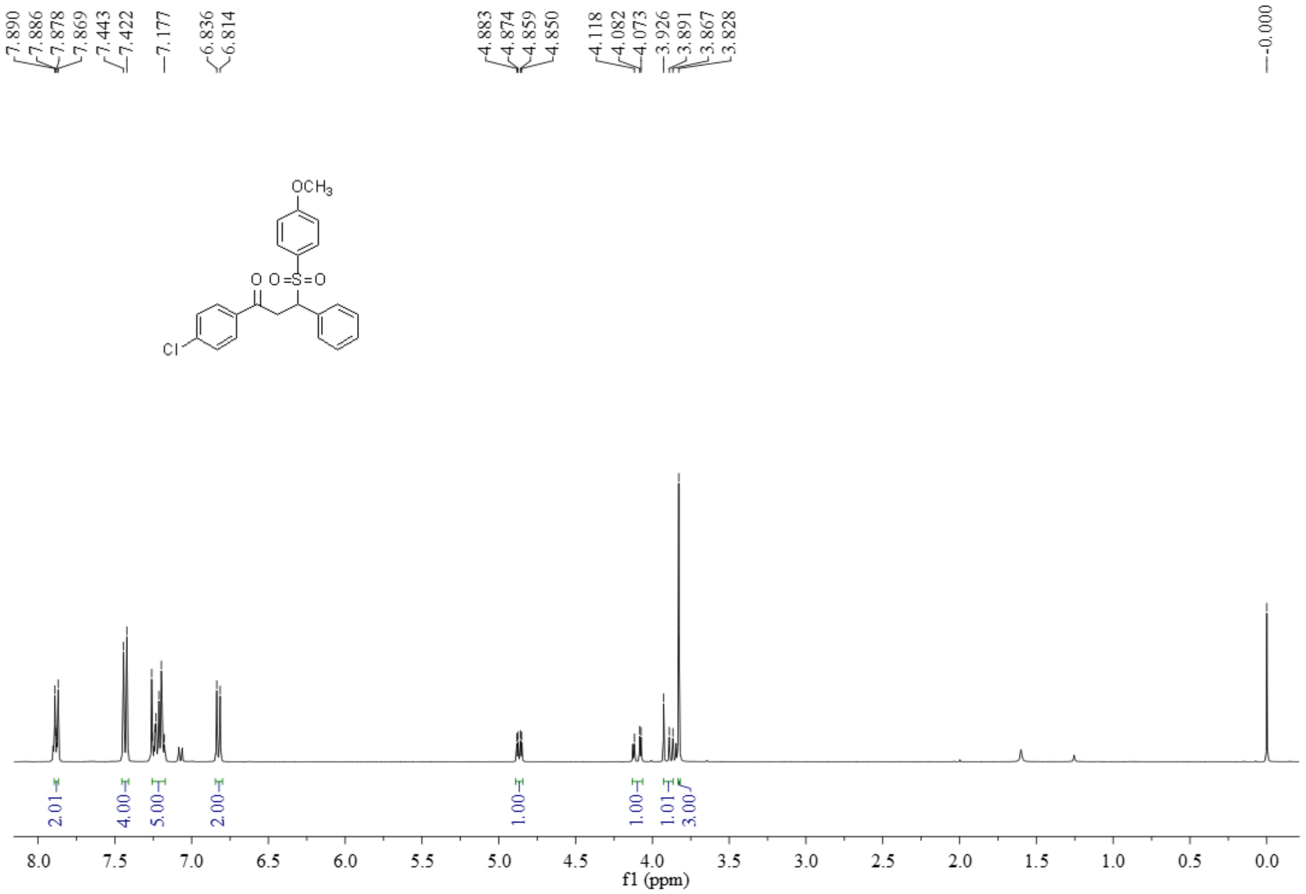


**$^{1}$H NMR Spectrum of Compound 5b**

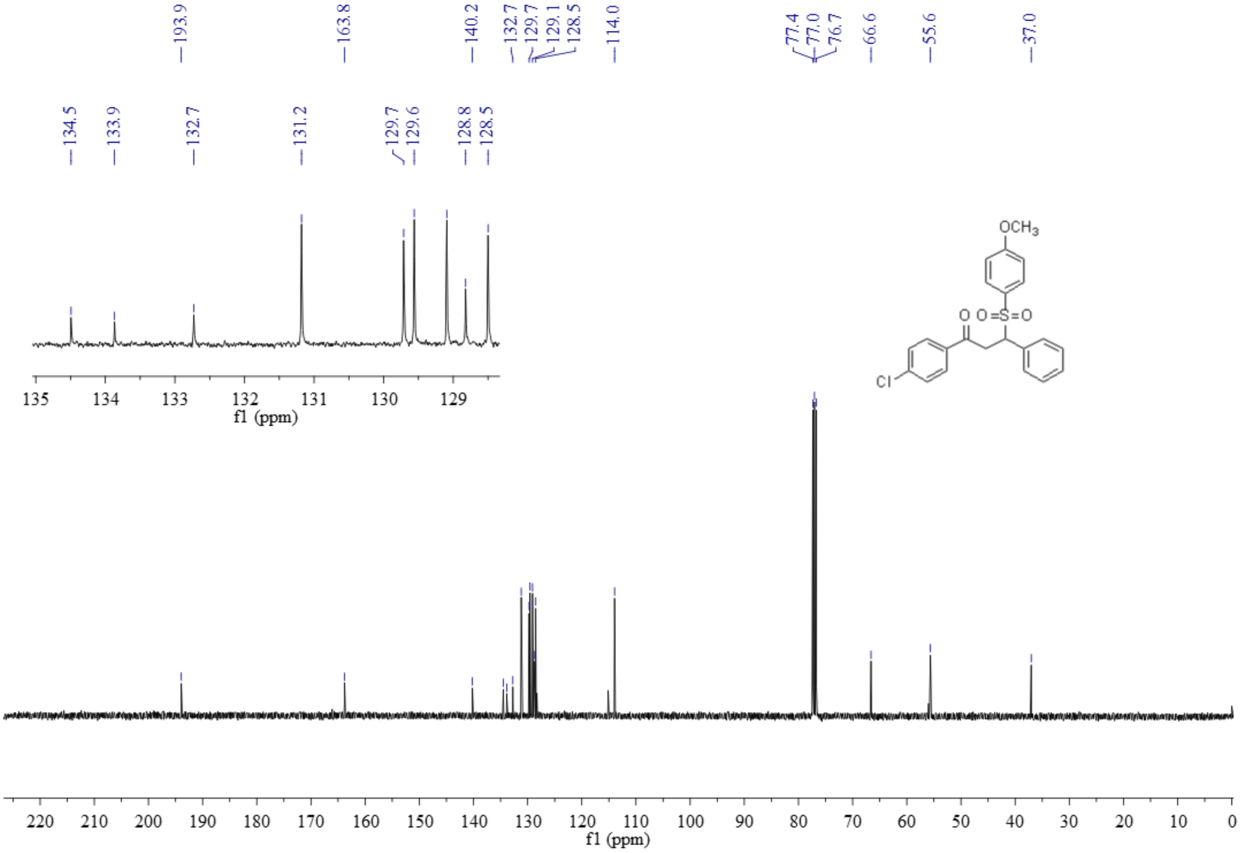


**$^{13}$C NMR Spectrum of Compound 5b**

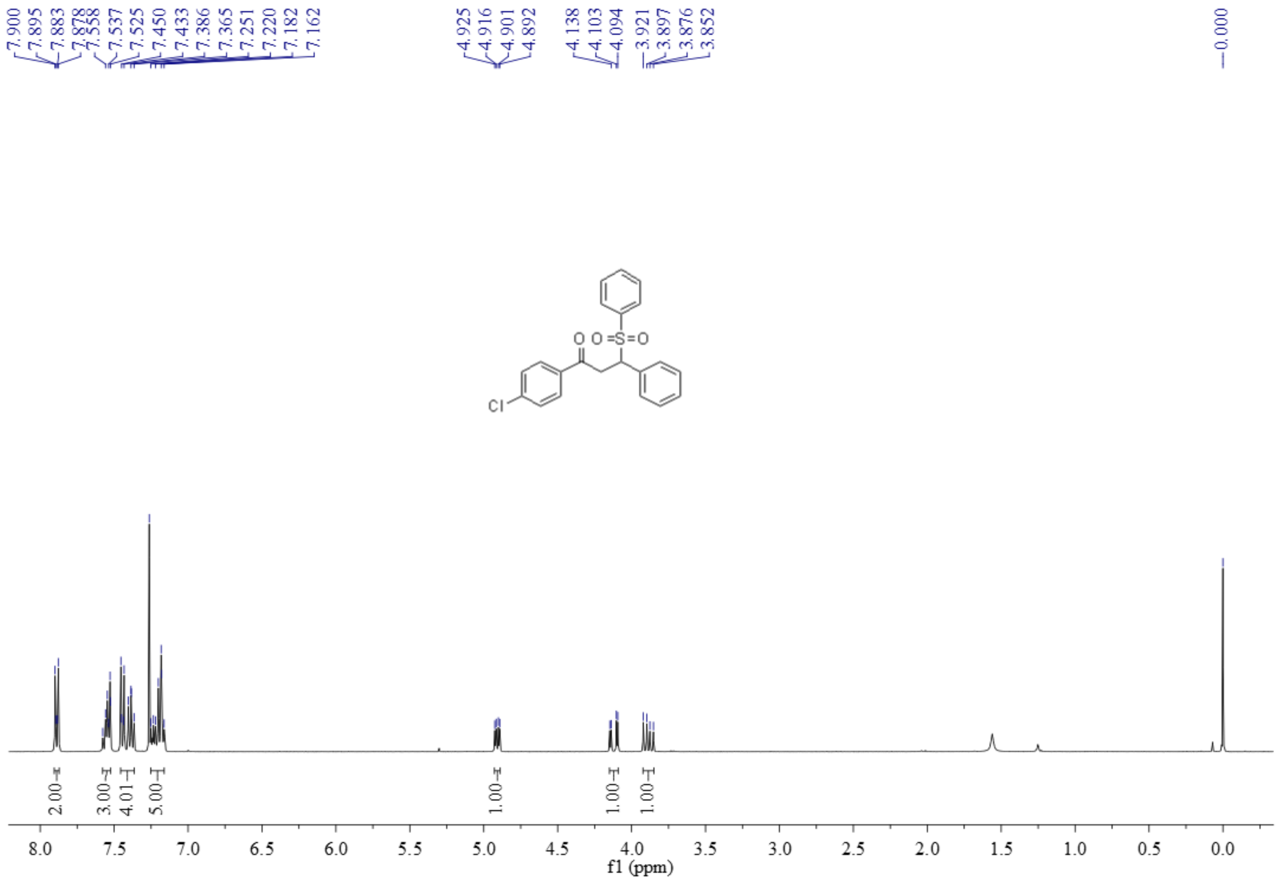

## $^{1}$H NMR Spectrum of Compound 5c

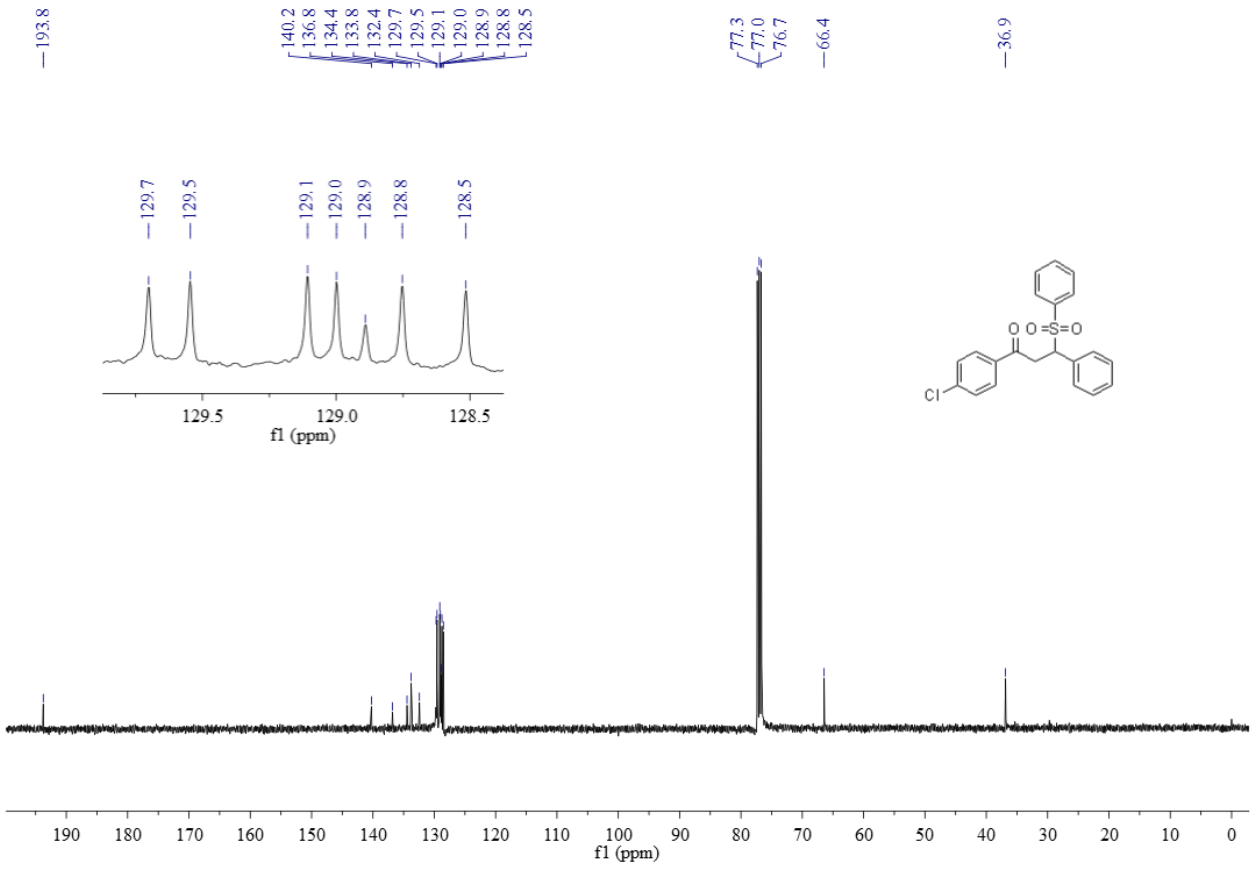

## $^{13}$C NMR Spectrum of Compound 5c

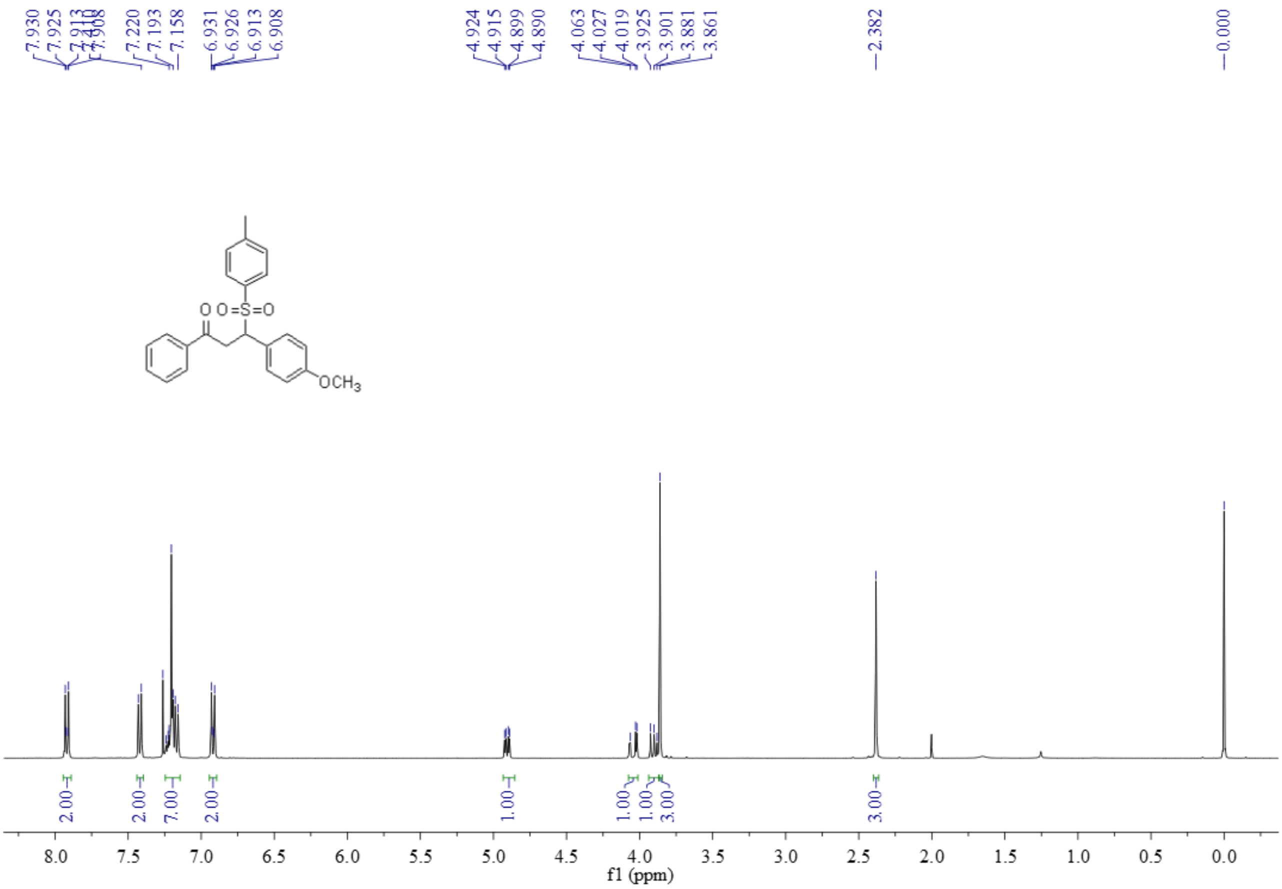


**$^1$H NMR Spectrum of Compound 5d**

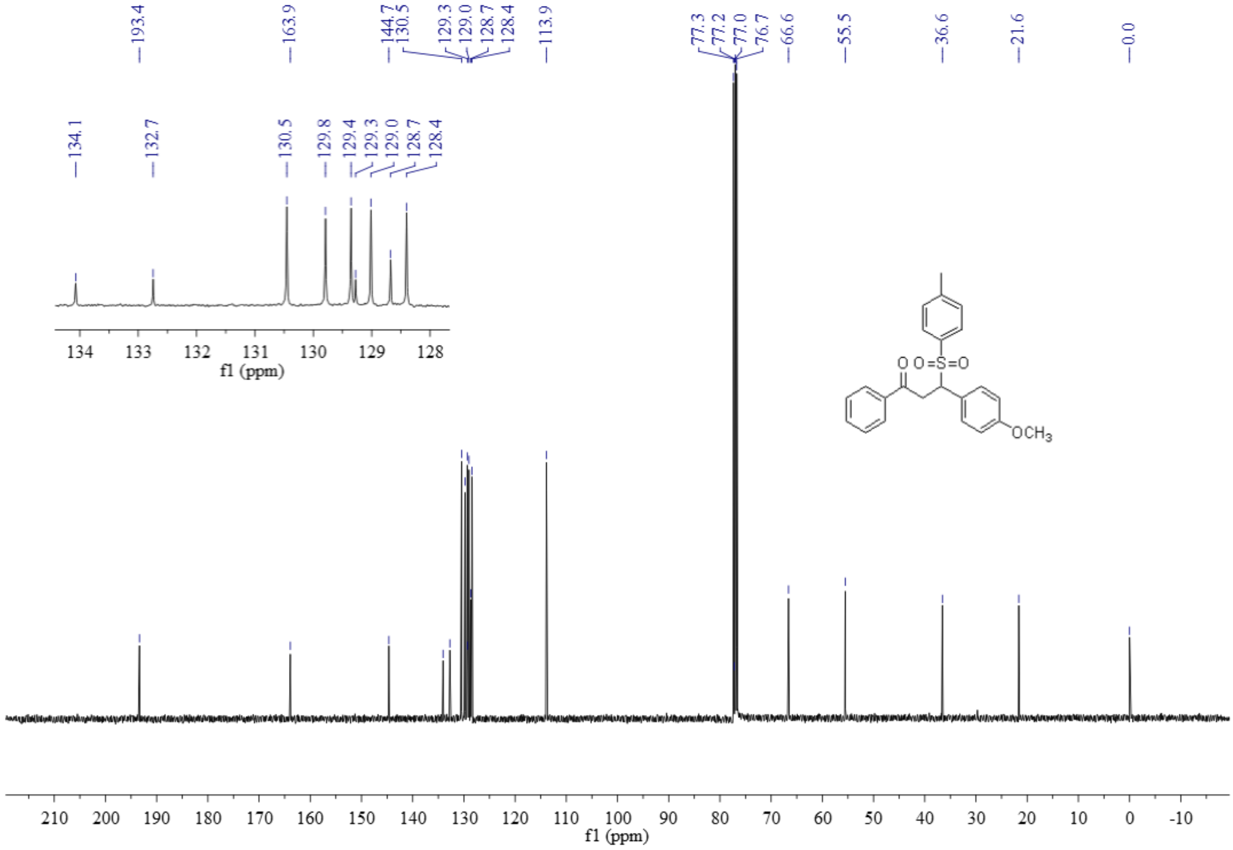


**$^{13}$C NMR Spectrum of Compound 5d**

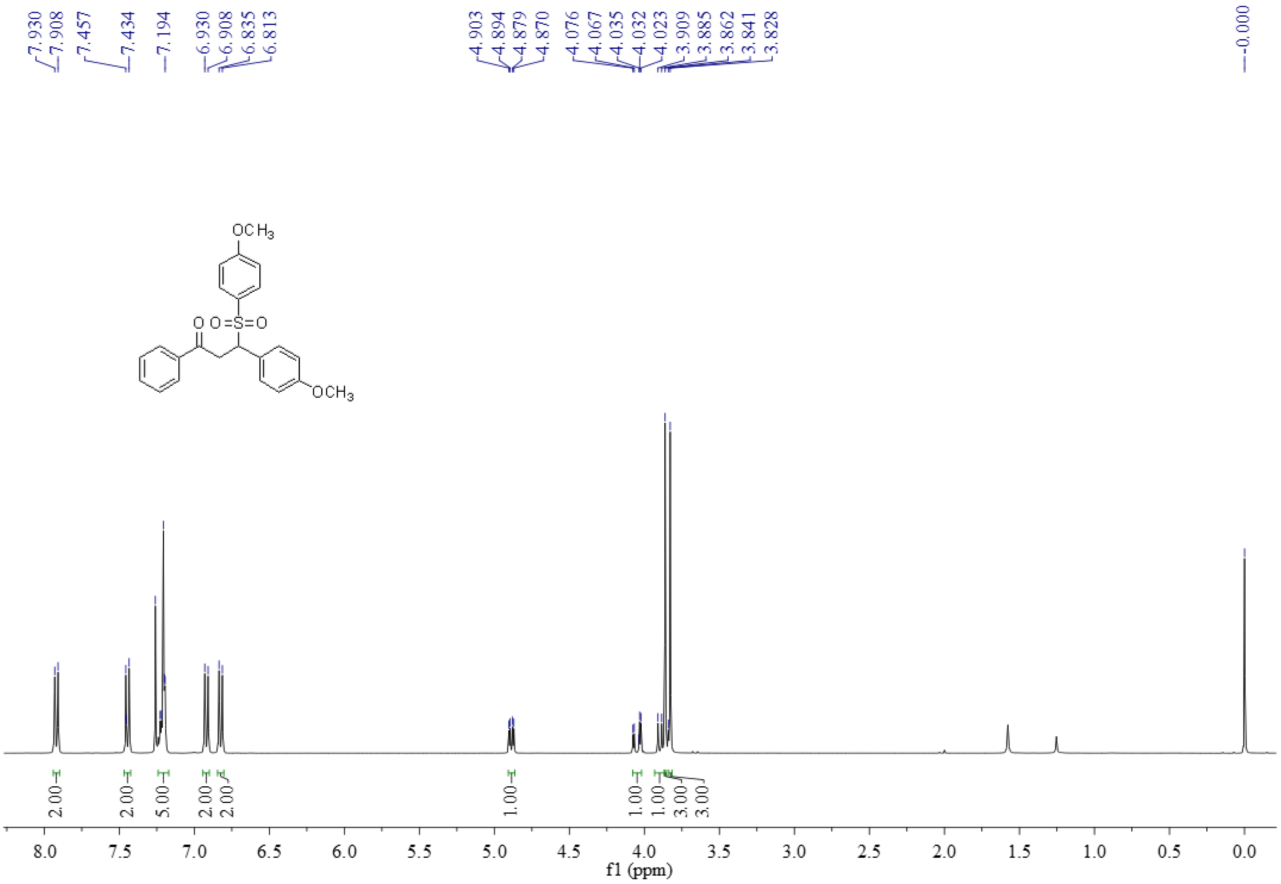

**$^{1}$H NMR Spectrum of Compound 5e**

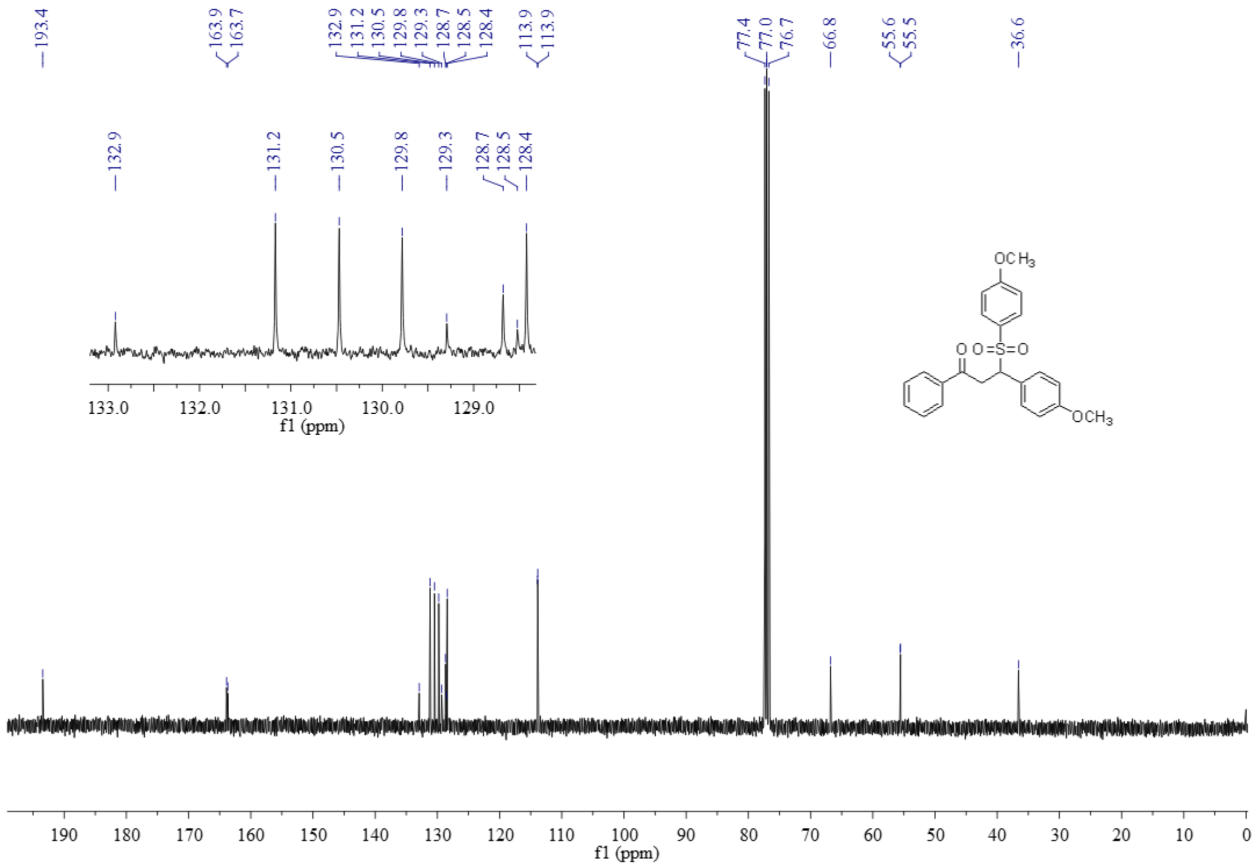

**$^{13}$C NMR Spectrum of Compound 5e**

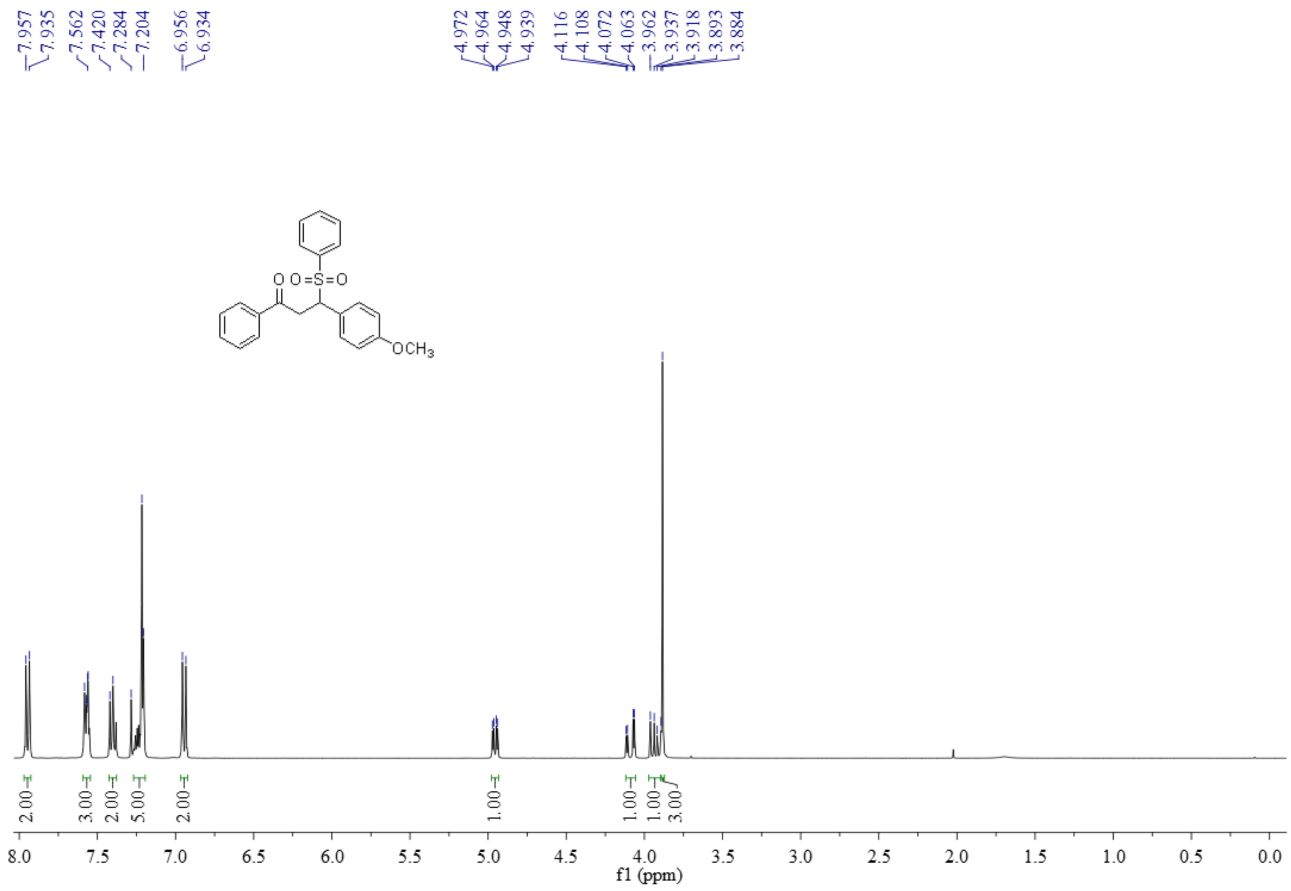

# $^{1}$H NMR Spectrum of Compound 5f

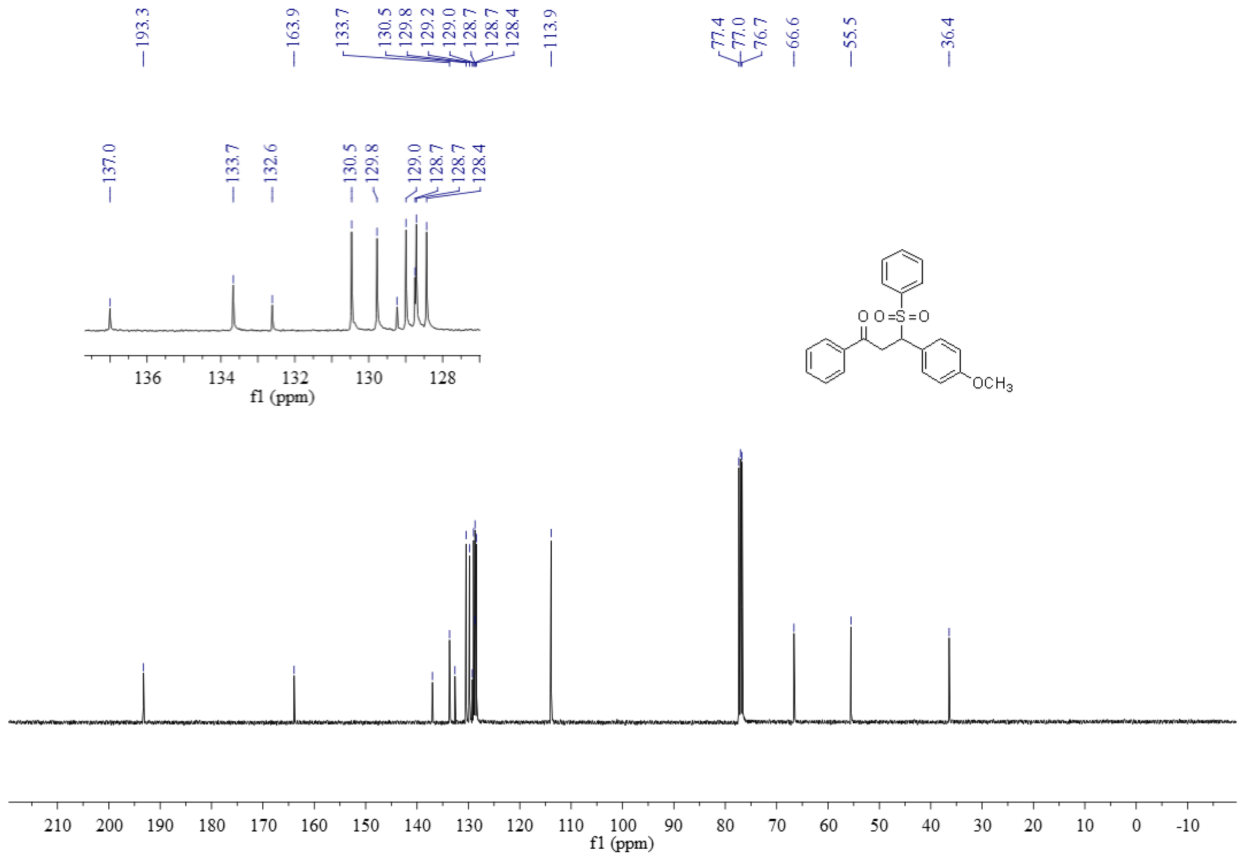

# $^{13}$C NMR Spectrum of Compound 5f

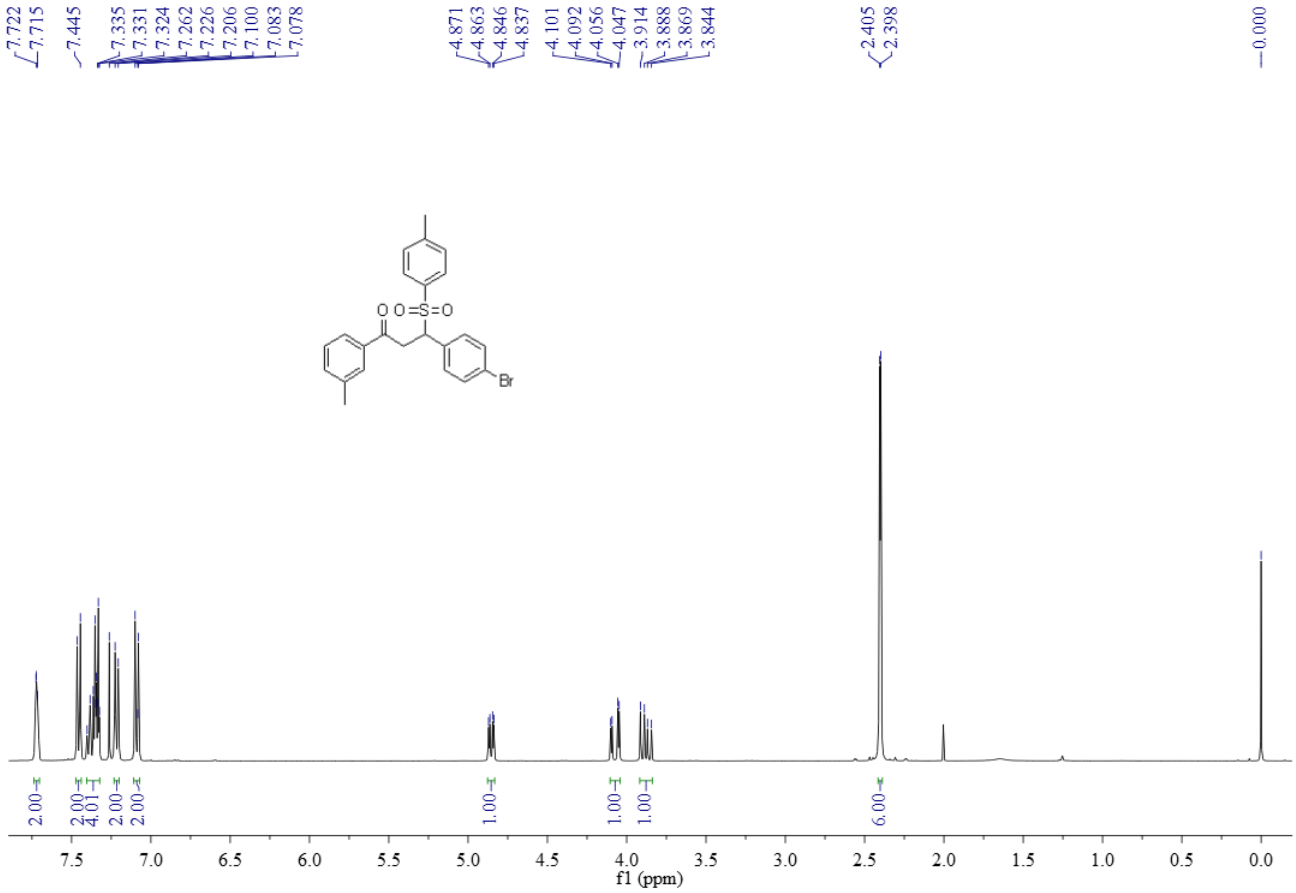

**$^{1}$H NMR Spectrum of Compound 5g**

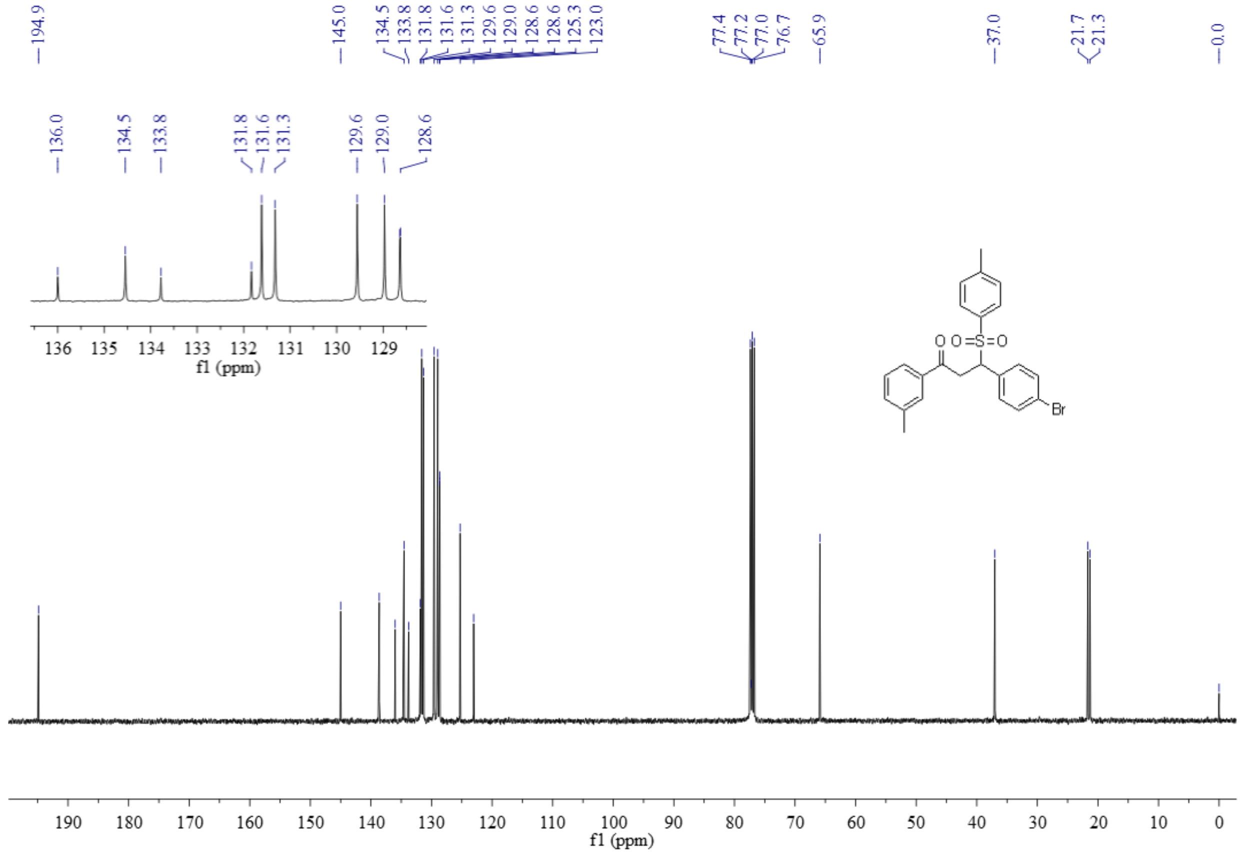

**$^{13}$C NMR Spectrum of Compound 5g**

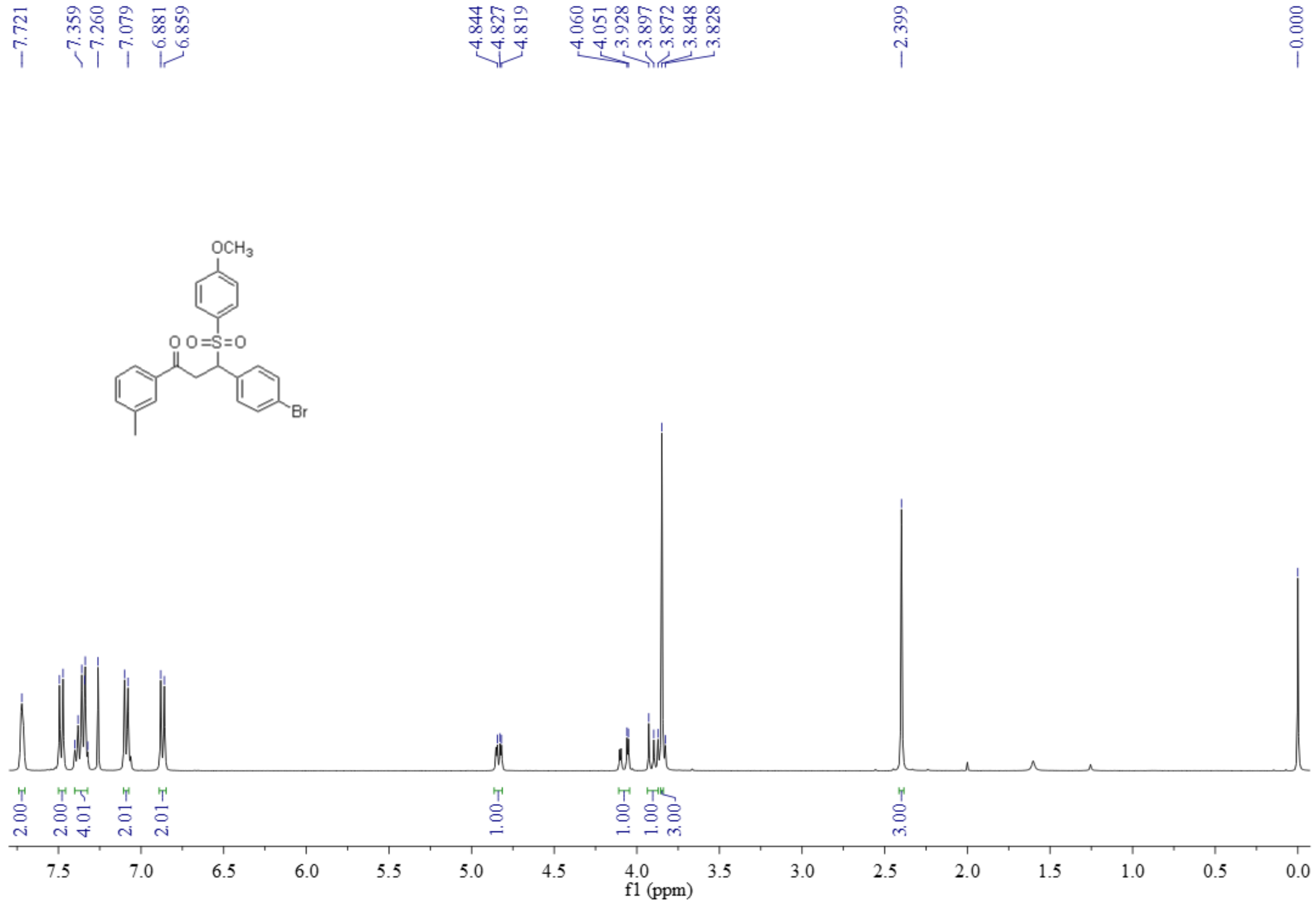

**$^{1}$H NMR Spectrum of Compound 5h**

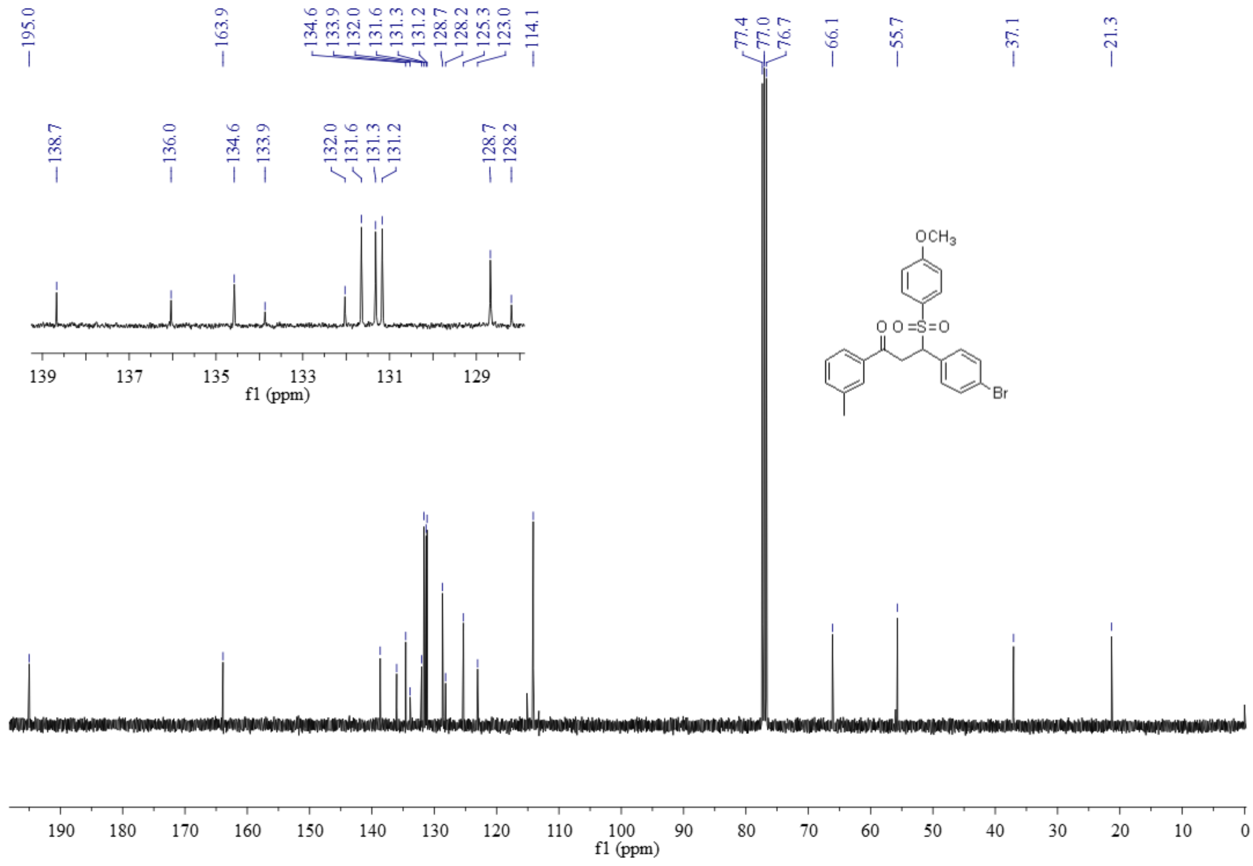

**$^{13}$C NMR Spectrum of Compound 5h**

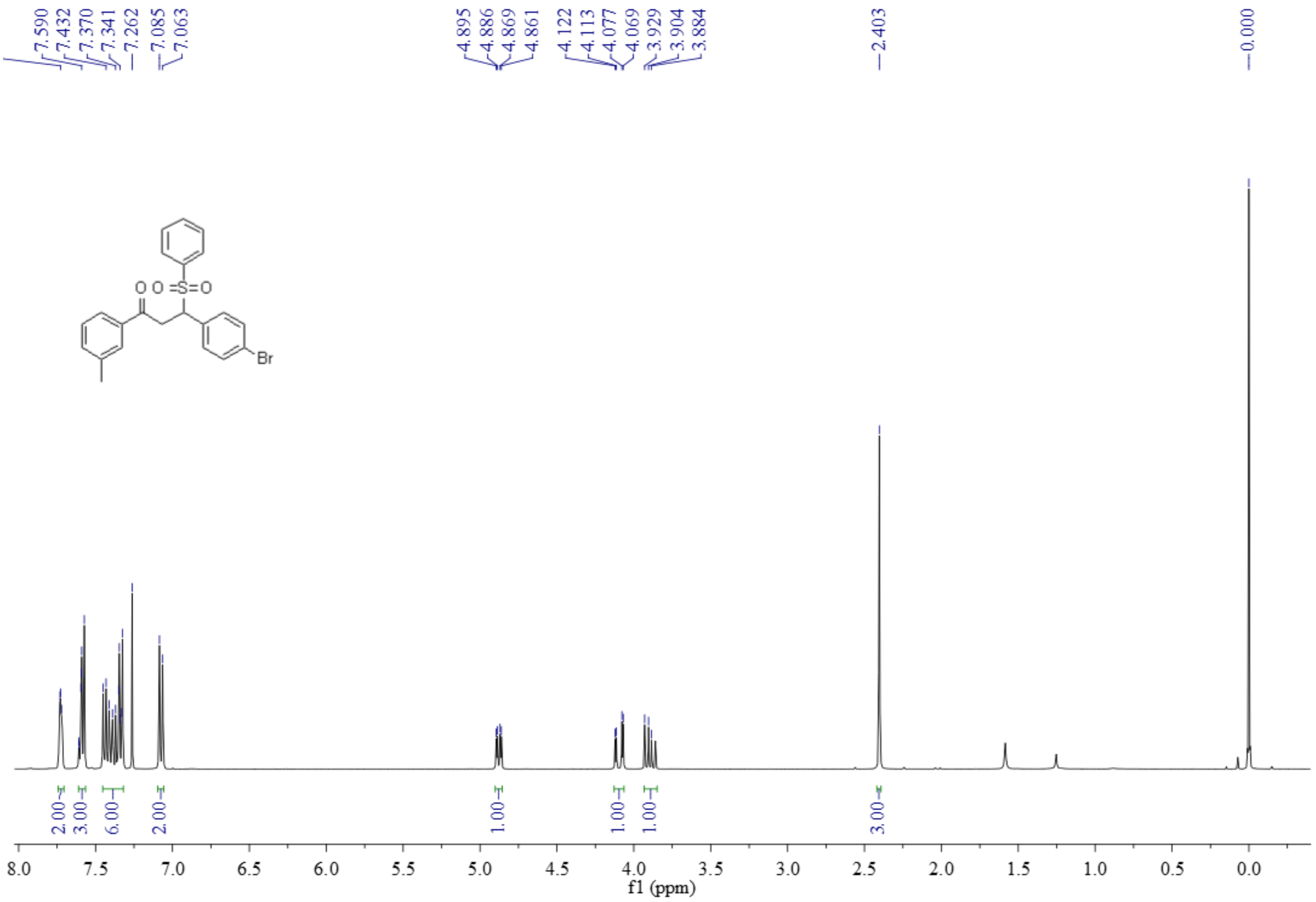


**$^{1}$H NMR Spectrum of Compound 5i**

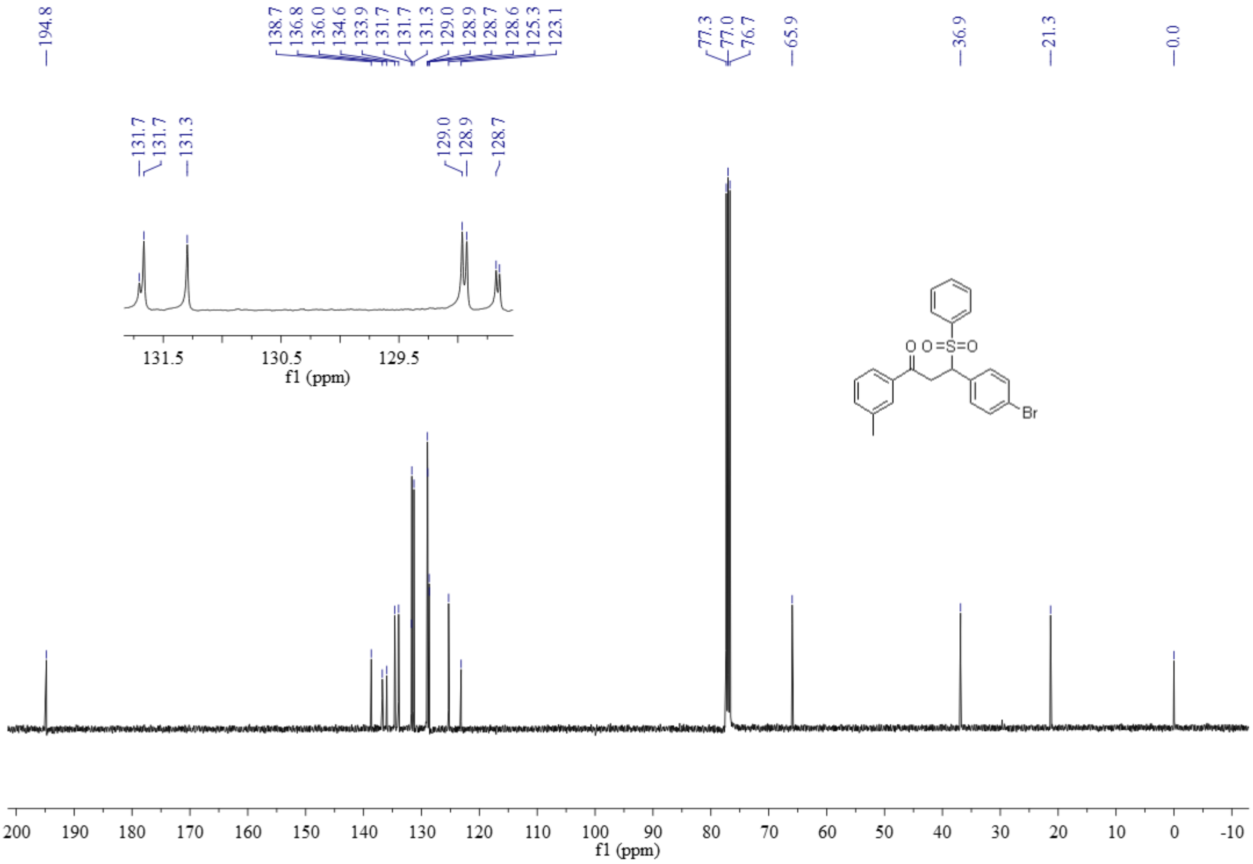


**$^{13}$C NMR Spectrum of Compound 5i**

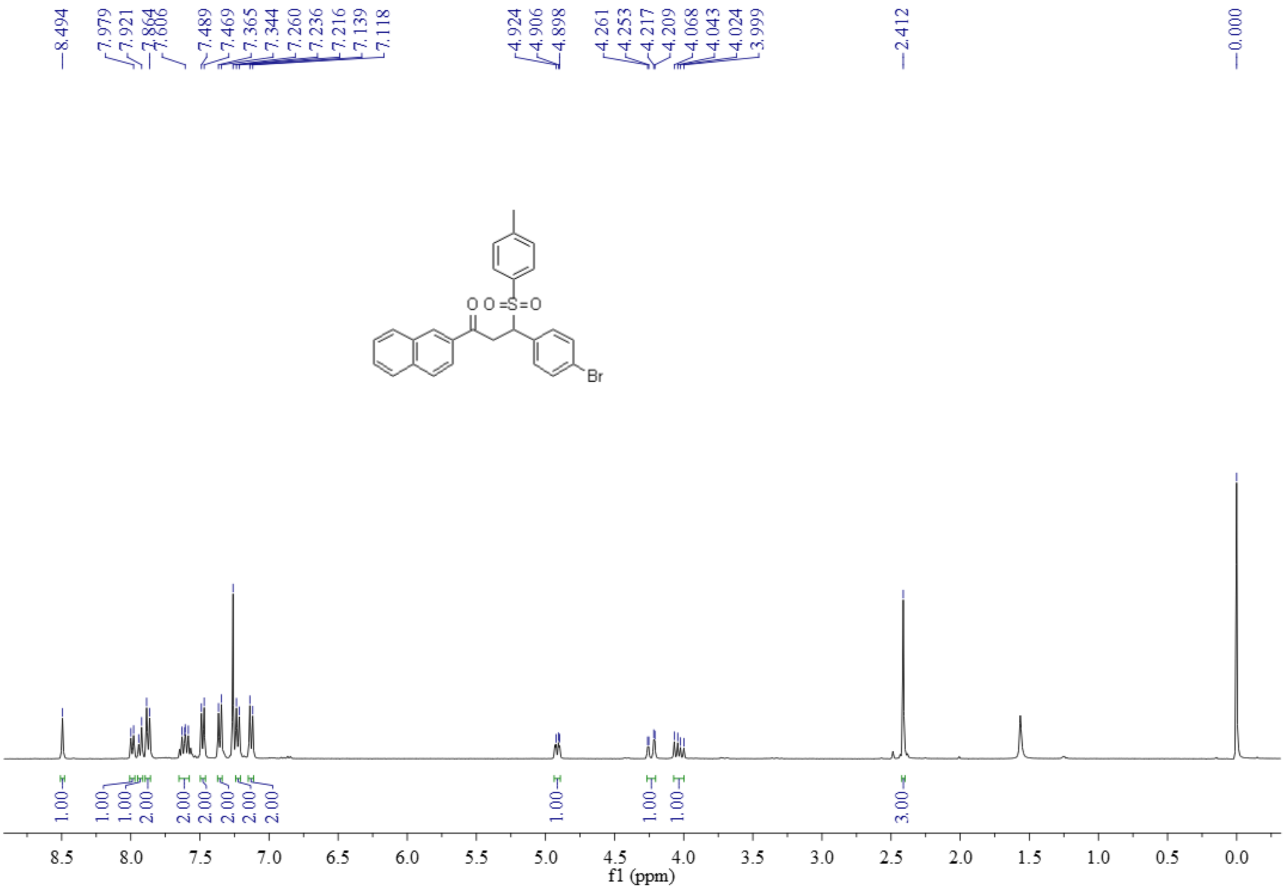


**$^{1}$H NMR Spectrum of Compound 5j**

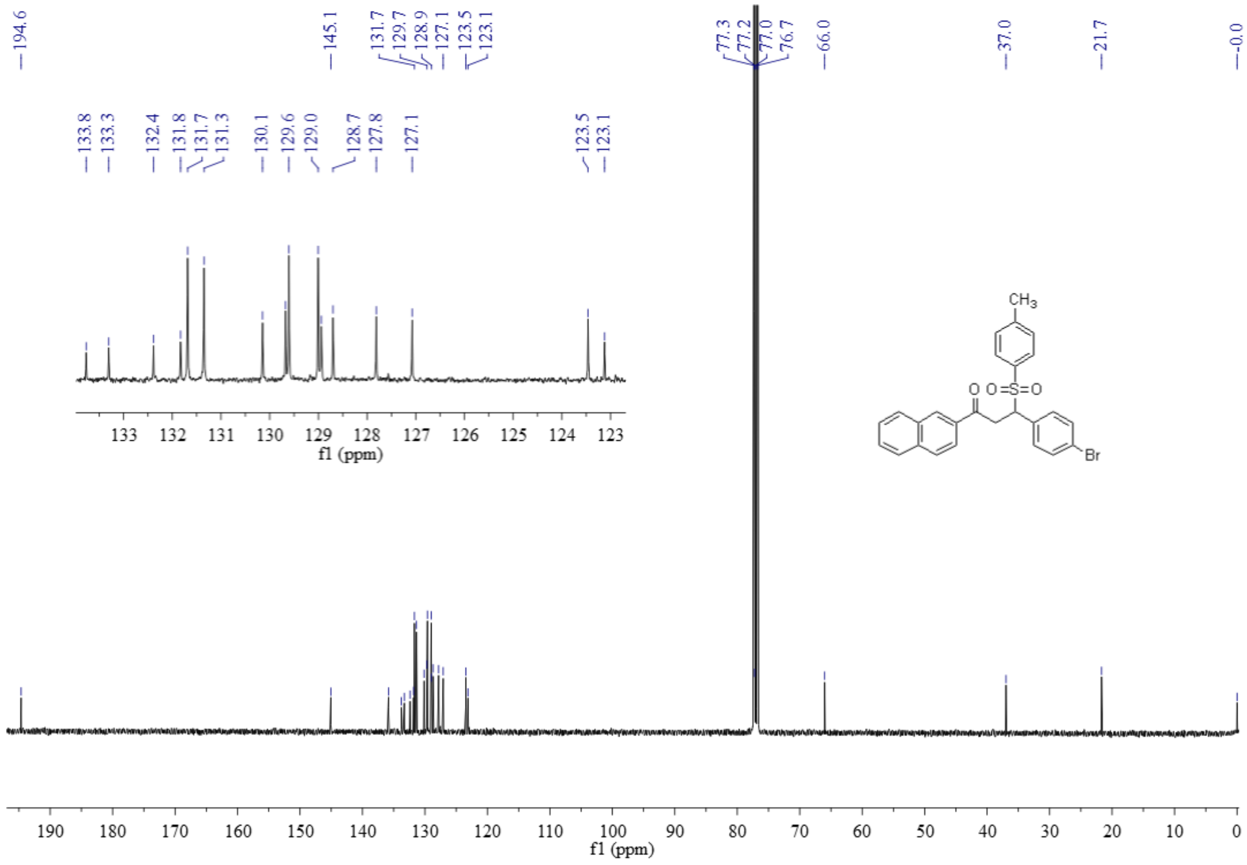


**$^{13}$C NMR Spectrum of Compound 5j**

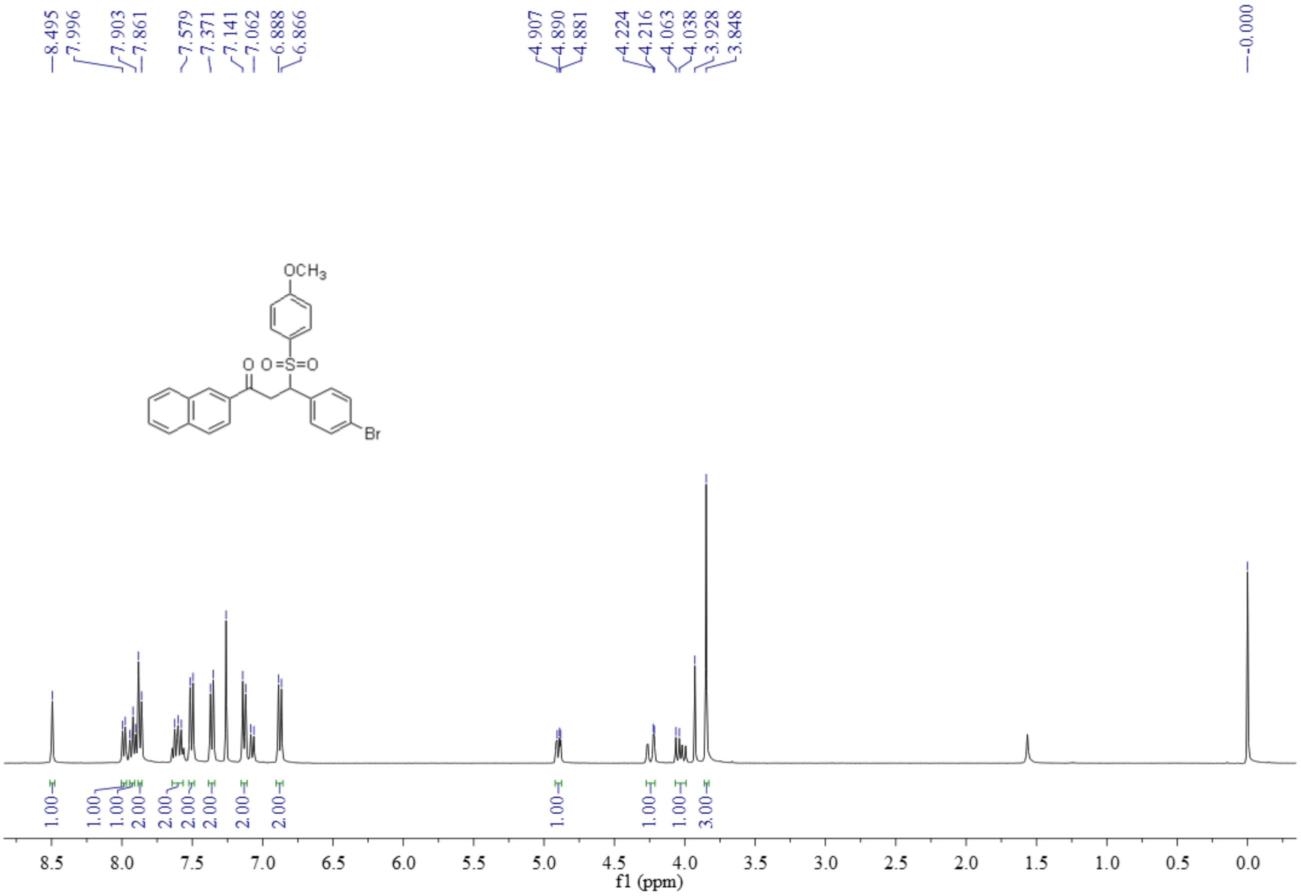


**$^{1}$H NMR Spectrum of Compound 5k**

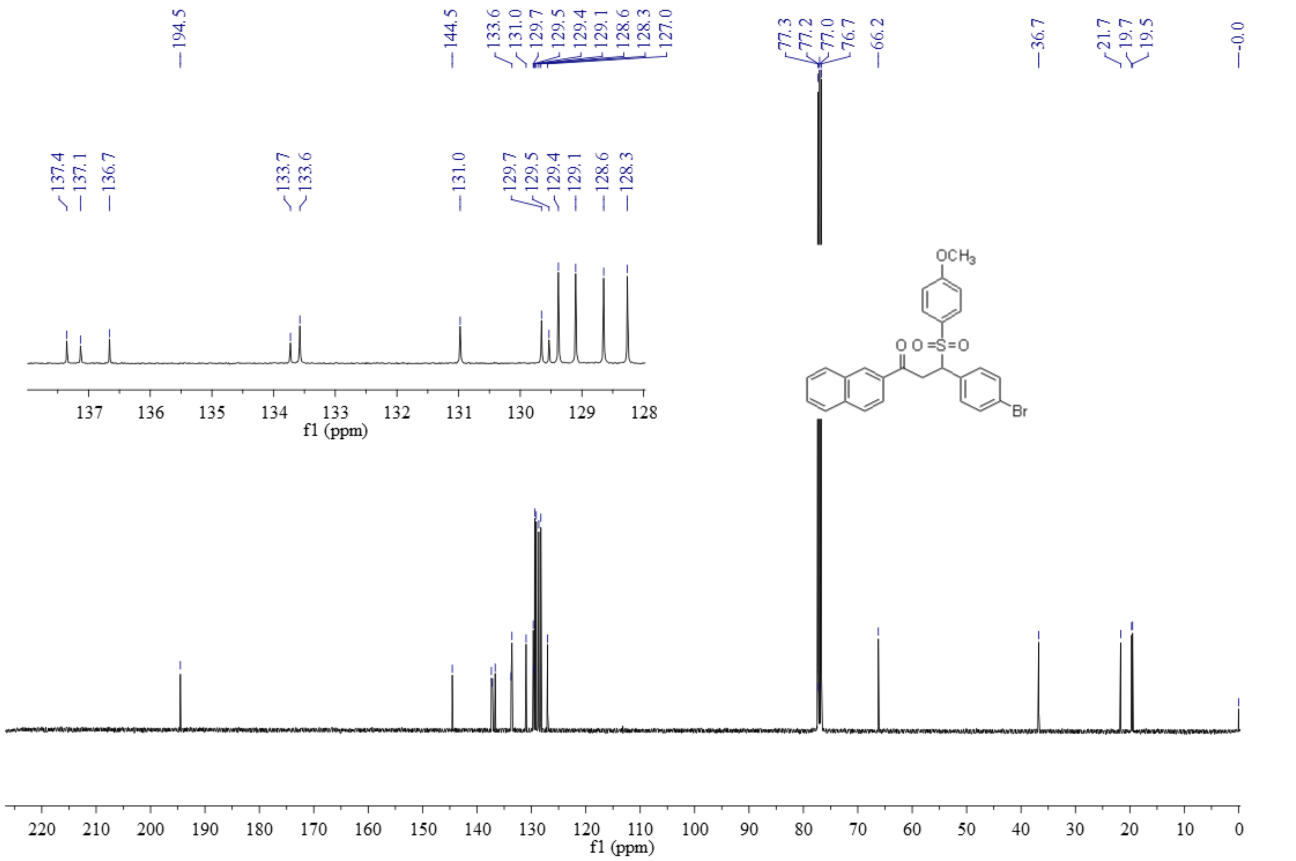


**$^{13}$C NMR Spectrum of Compound 5k**

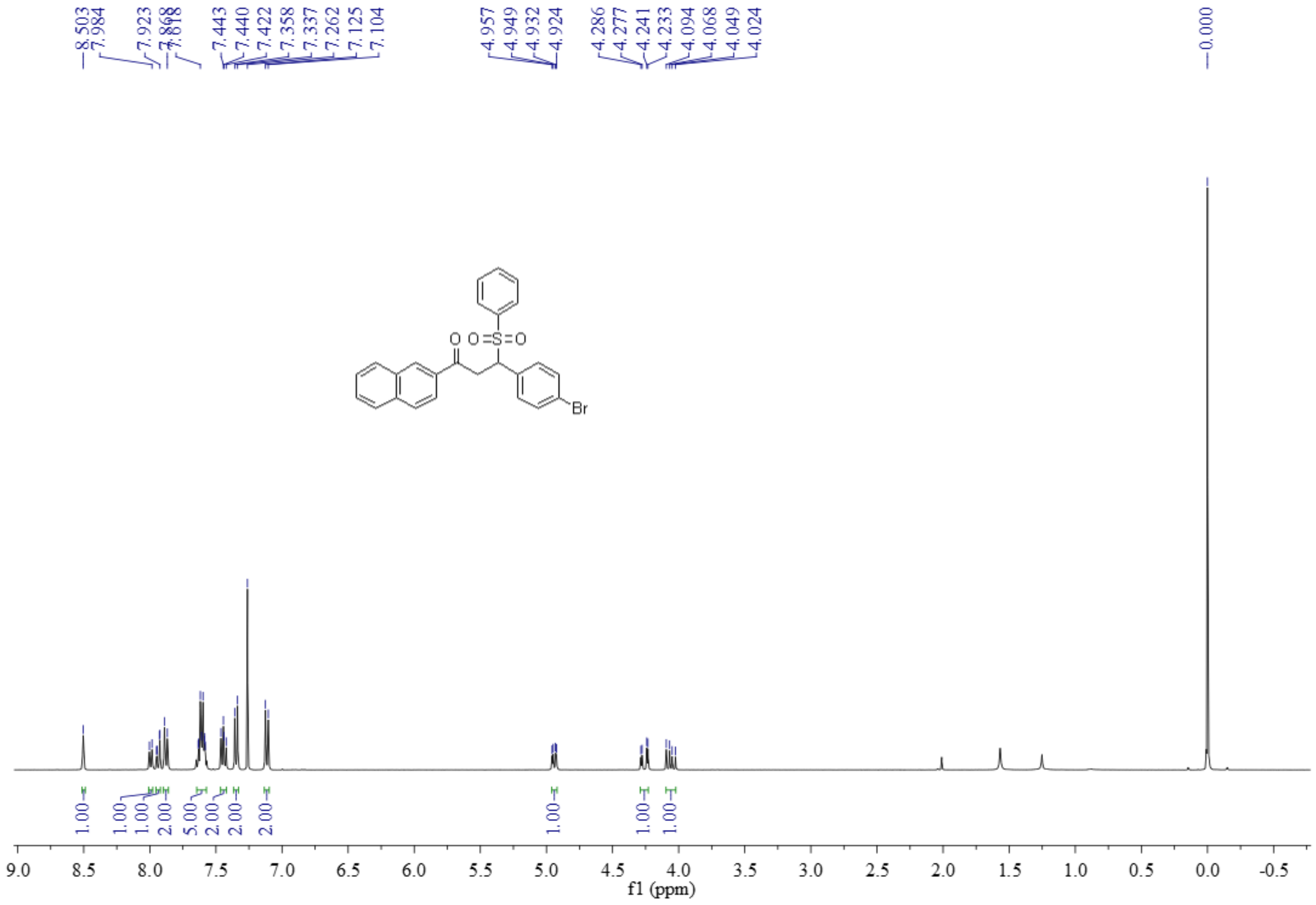

**$^{1}$H NMR Spectrum of Compound 5l**

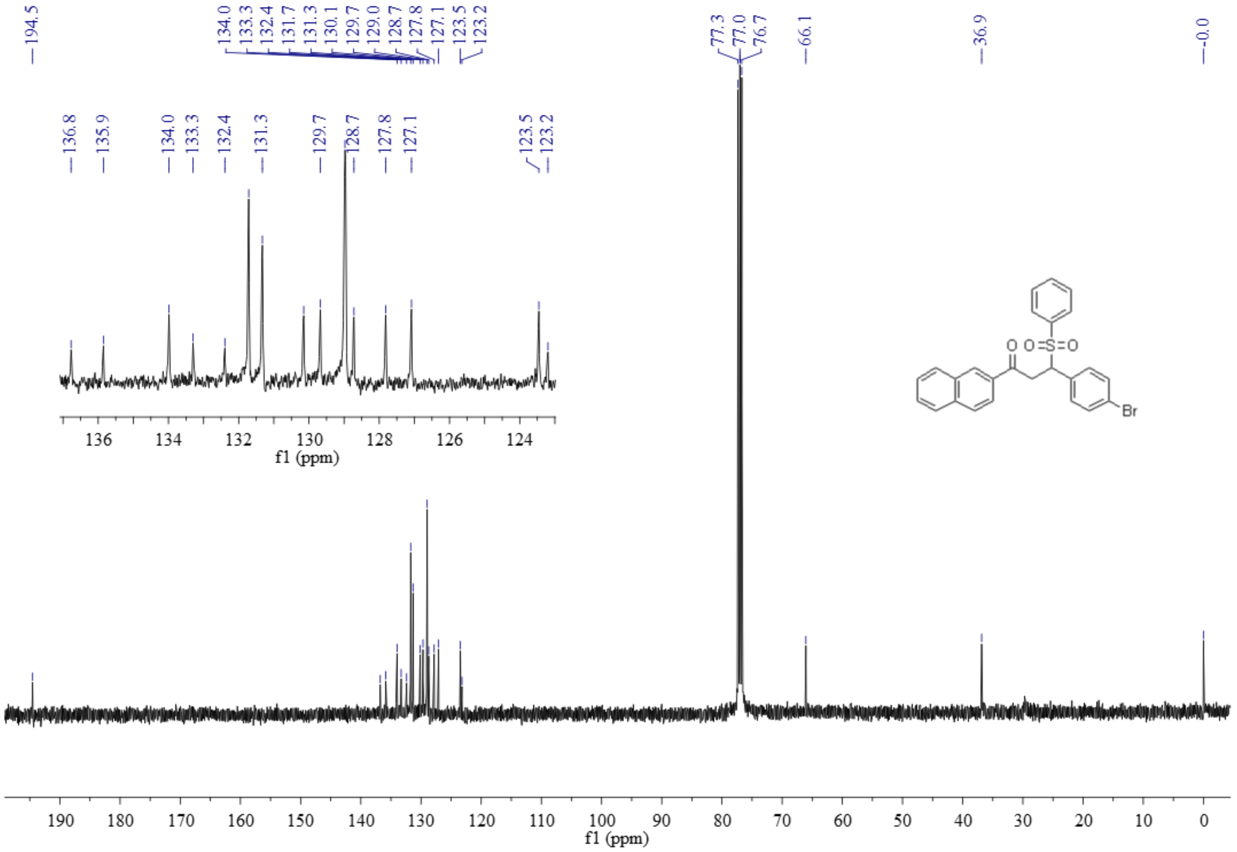

**$^{13}$C NMR Spectrum of Compound 5l**

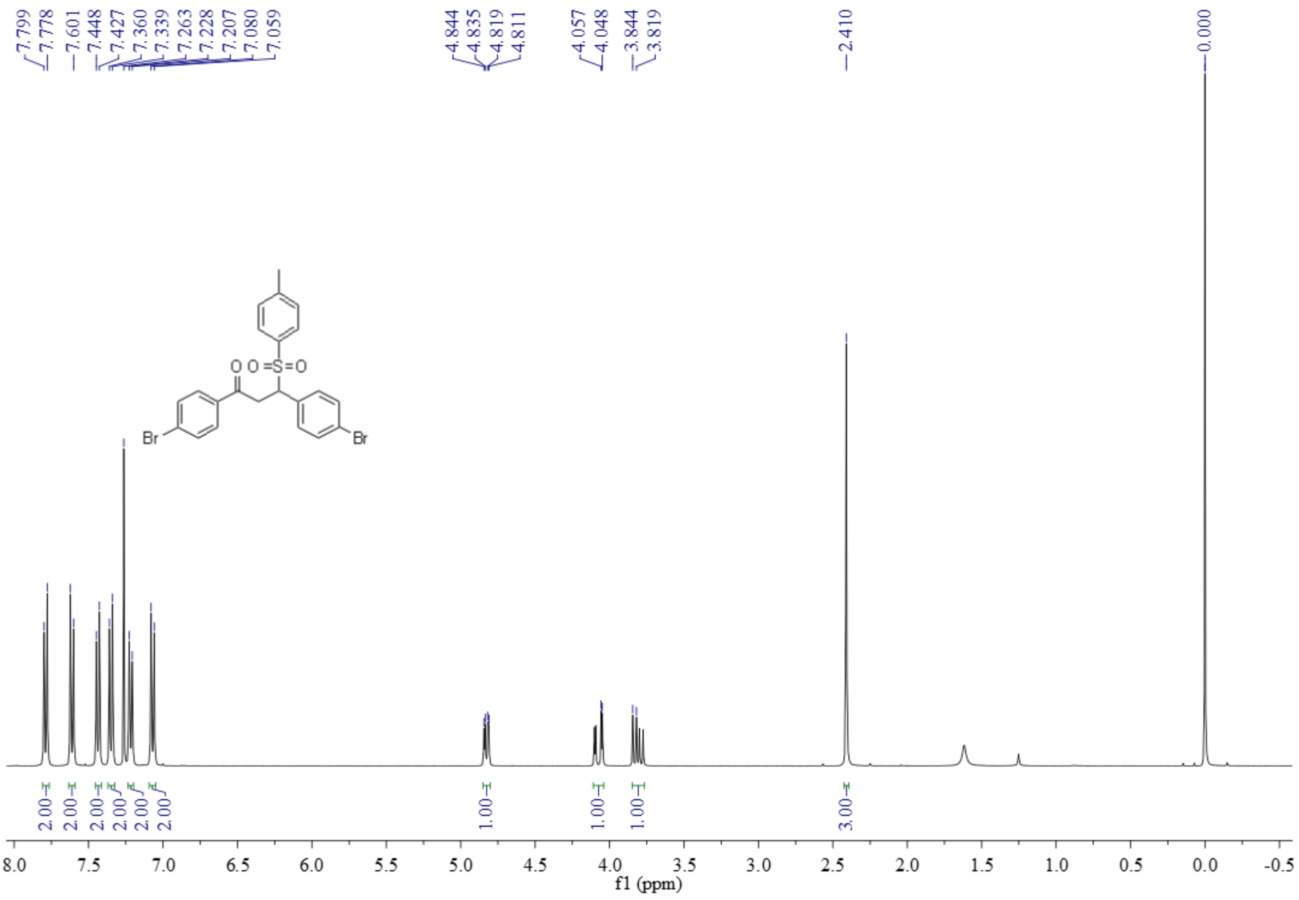

**$^{1}$H NMR Spectrum of Compound 5m**

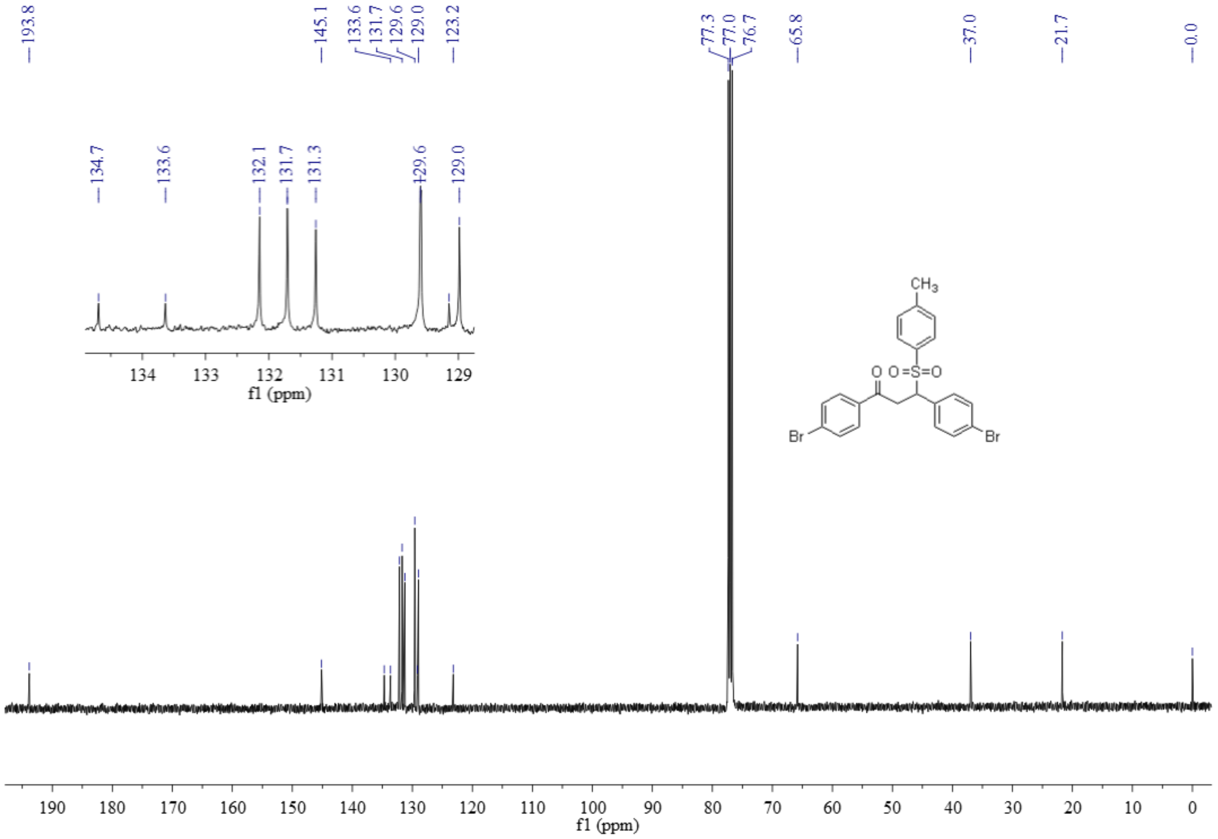

**$^{13}$C NMR Spectrum of Compound 5m**

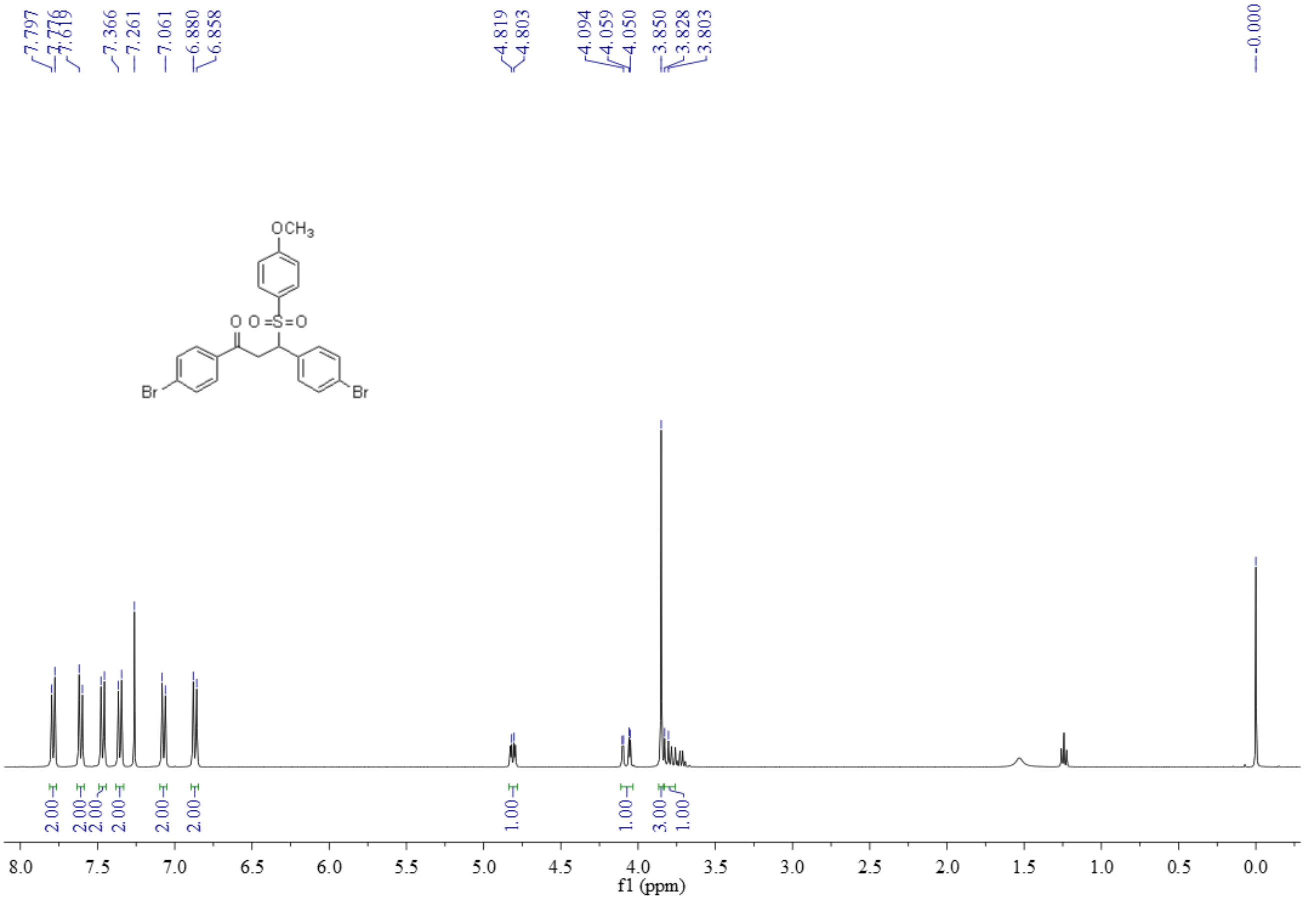


**$^{1}$H NMR Spectrum of Compound 5n**

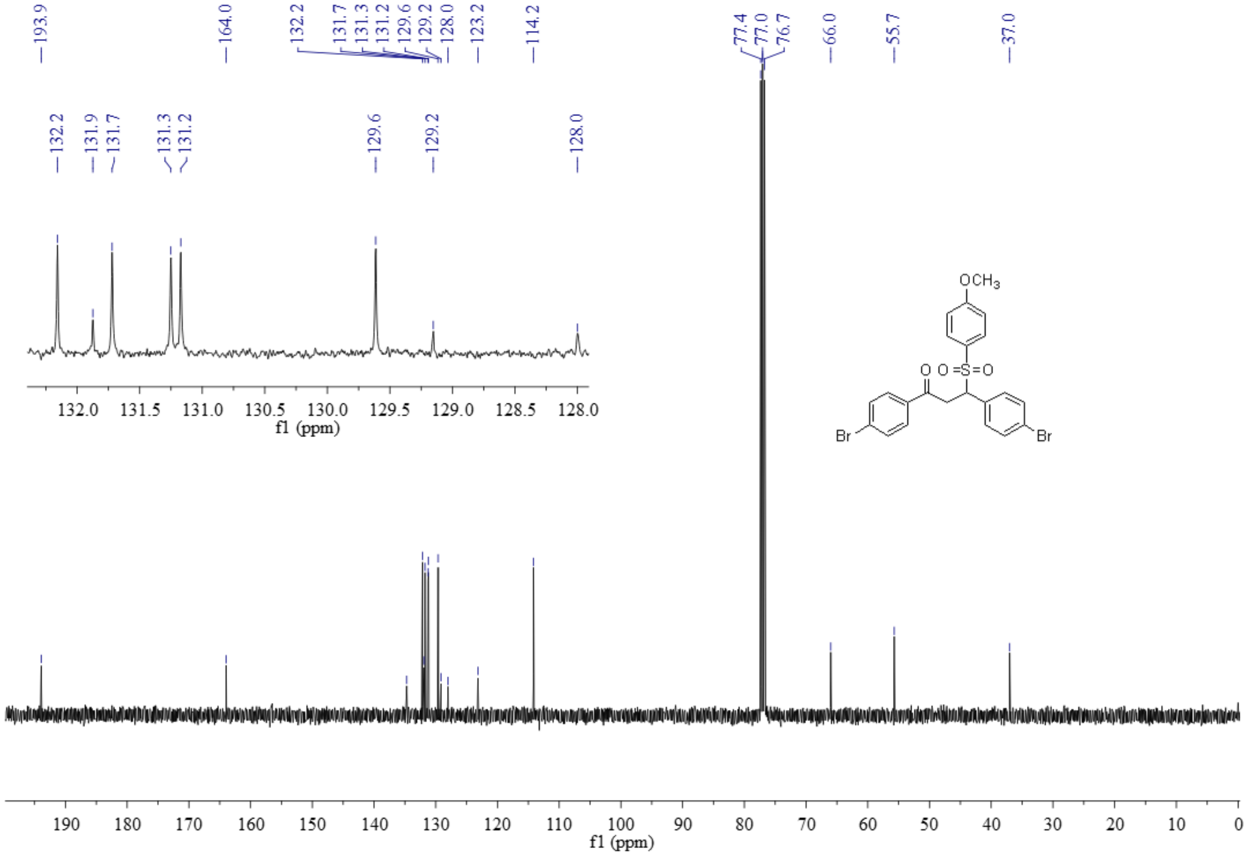


**$^{13}$C NMR Spectrum of Compound 5n**

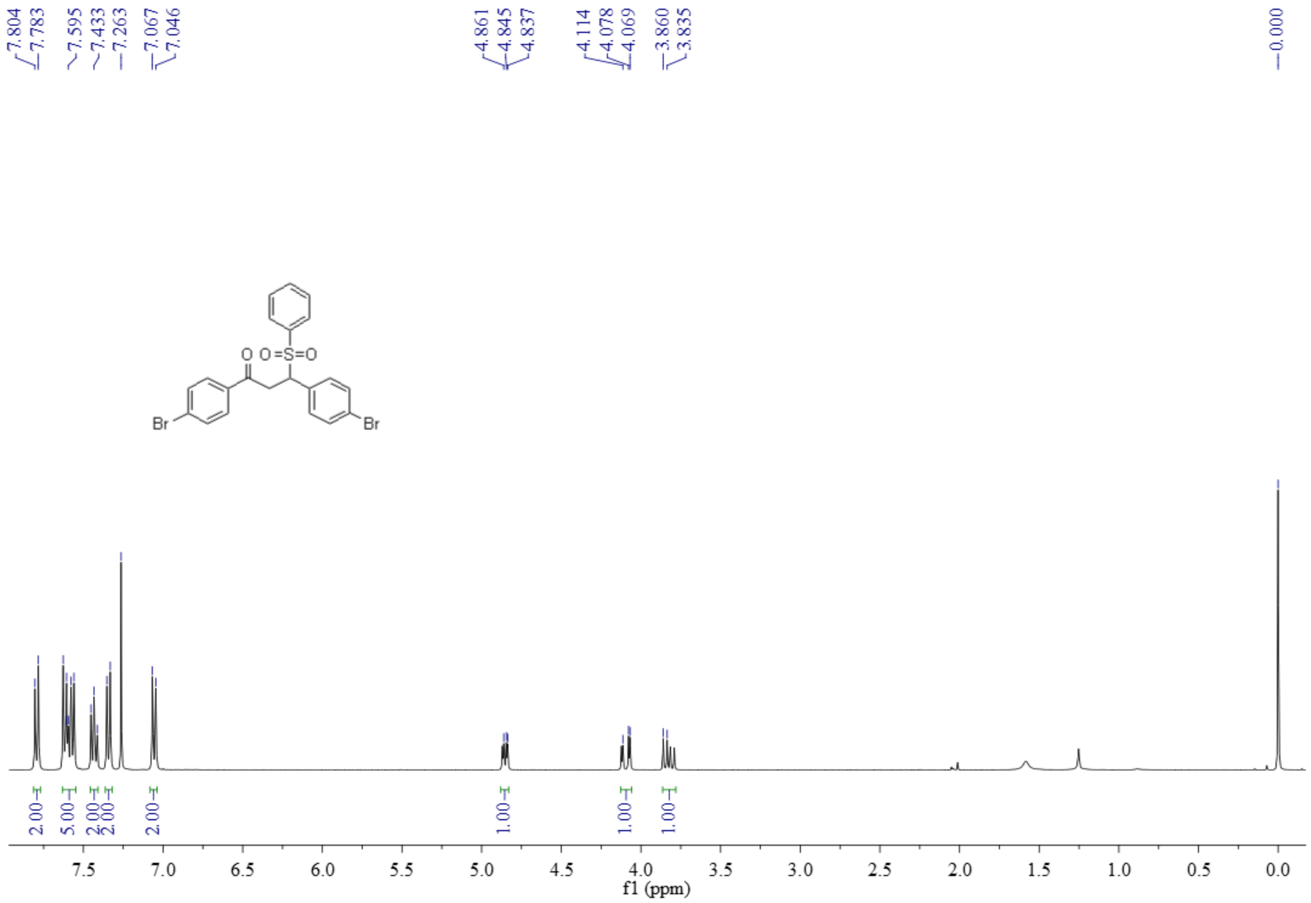


**$^{1}$H NMR Spectrum of Compound 5o**

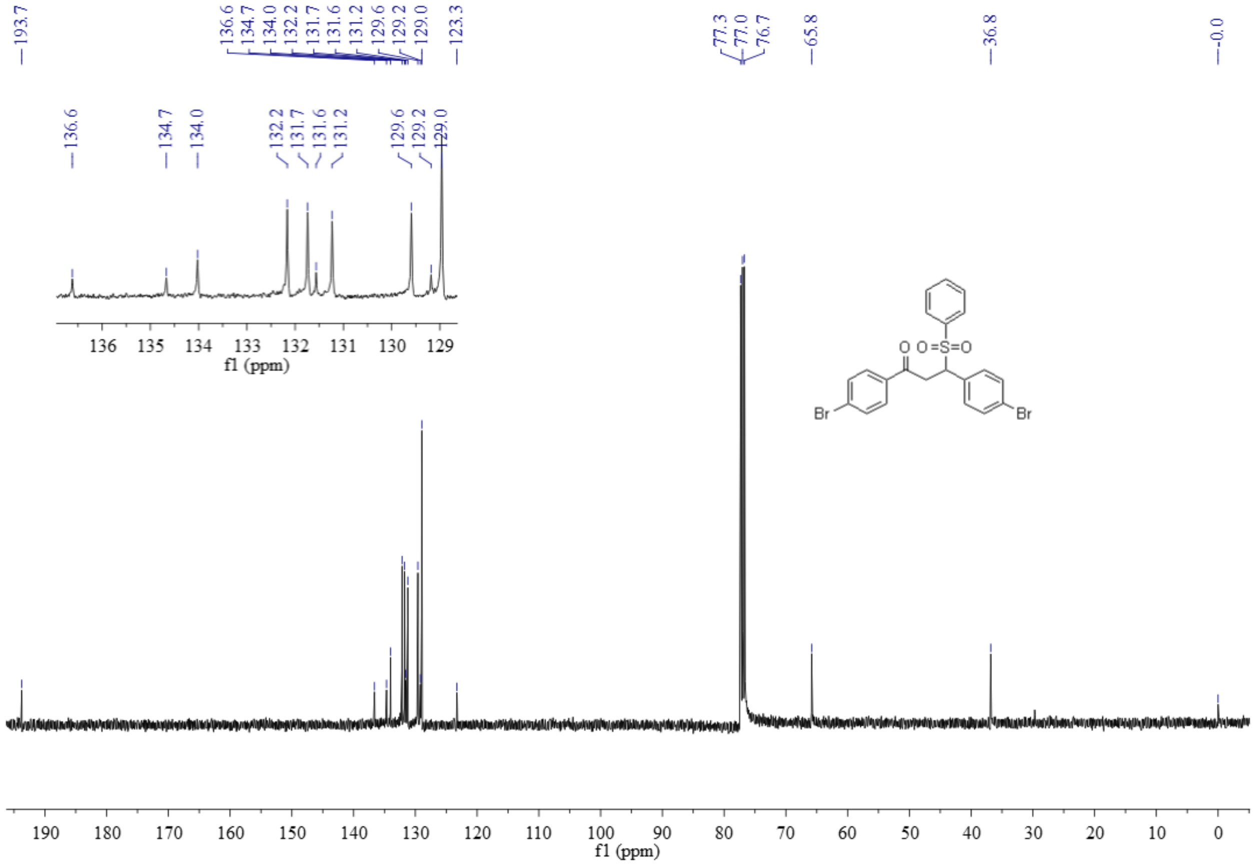


**$^{13}$C NMR Spectrum of Compound 5o**
